# Evolution of myxozoan mitochondrial genomes: insights from myxobolids

**DOI:** 10.1186/s12864-024-10254-w

**Published:** 2024-04-22

**Authors:** Tatiana Orli Milkewitz Sandberg, Dayana Yahalomi, Noam Bracha, Michal Haddas-Sasson, Tal Pupko, Stephen D. Atkinson, Jerri L. Bartholomew, Jin Yong Zhang, Dorothée Huchon

**Affiliations:** 1 School of Zoology, George S. Wise Faculty of Life Sciences, Tel Aviv University, 6997801 Tel Aviv, Israel; 2 The Shmunis School of Biomedicine and Cancer Research, George S. Wise Faculty of Life Sciences, Tel Aviv University, 6997801 Tel Aviv, Israel; 3 Department of Microbiology, Oregon State University, 97331 Corvallis, OR USA; 4 School of Marine Science and Engineering, Qingdao Agricultural University, Qingdao, China; 5 The Steinhardt Museum of Natural History and National Research Center, Tel Aviv University, 6997801 Tel Aviv, Israel

**Keywords:** Cnidaria, MtDNA, Molecular evolution, Organellar genome, Phylogenomics

## Abstract

**Background:**

Myxozoa is a class of cnidarian parasites that encompasses over 2,400 species. Phylogenetic relationships among myxozoans remain highly debated, owing to both a lack of informative morphological characters and a shortage of molecular markers. Mitochondrial (mt) genomes are a common marker in phylogeny and biogeography. However, only five complete myxozoan mt genomes have been sequenced: four belonging to two closely related genera, *Enteromyxum* and *Kudoa*, and one from the genus *Myxobolus*. Interestingly, while cytochrome oxidase genes could be identified in *Enteromyxum* and *Kudoa*, no such genes were found in *Myxobolus squamalis*, and another member of the Myxobolidae (*Henneguya salminicola*) was found to have lost its entire mt genome. To evaluate the utility of mt genomes to reconstruct myxozoan relationships and to understand if the loss of cytochrome oxidase genes is a characteristic of myxobolids, we sequenced the mt genome of five myxozoans (*Myxobolus wulii, M. honghuensis*, *M. shantungensis, Thelohanellus kitauei* and, *Sphaeromyxa zaharoni*) using Illumina and Oxford Nanopore platforms.

**Results:**

Unlike *Enteromyxum*, which possesses a partitioned mt genome, the five mt genomes were encoded on single circular chromosomes. An mt plasmid was found in *M. wulii*, as described previously in *Kudoa iwatai*. In all new myxozoan genomes, five protein-coding genes (*cob, cox1, cox2, nad1*, and *nad5*) and two rRNAs (*rnl* and *rns*) were recognized, but no tRNA. We found that *Myxobolus* and *Thelohanellus* species shared unidentified reading frames, supporting the view that these mt open reading frames are functional. Our phylogenetic reconstructions based on the five conserved mt genes agree with previously published trees based on the 18S rRNA gene.

**Conclusions:**

Our results suggest that the loss of cytochrome oxidase genes is not a characteristic of all myxobolids, the ancestral myxozoan mt genome was likely encoded on a single circular chromosome, and mt plasmids exist in a few lineages. Our findings indicate that myxozoan mt sequences are poor markers for reconstructing myxozoan phylogenetic relationships because of their fast-evolutionary rates and the abundance of repeated elements, which complicates assembly.

**Supplementary Information:**

The online version contains supplementary material available at 10.1186/s12864-024-10254-w.

## Introduction

The class Myxozoa (Grassé, 1970) comprises over 2,400 species of endoparasitic cnidarians. The life cycle of myxozoans involves two hosts, usually a fish and an annelid [1]. Previous phylogenomic analyses of protein coding genes and mitochondrial (mt) genome structure have strongly supported the monophyly of Myxozoa and their position as a sister clade to *Polypodium hydriforme* [2, 3].

Myxozoa consist of two subclasses, the Malacosporea and the Myxosporea [4], encompassing about ten [5] and over 2,300 species, respectively. Morphological classification of Myxozoa has been impeded by the paucity of morphological characters in their spore stages. Consequently, molecular markers have become the gold standard to reconstruct myxozoan phylogenetic relationships [4]. Molecular phylogenies support the separation of Myxosporea into two clades, the freshwater/oligochaete-host clade and the marine/polychaete-host clade. The phylogenetic relationships within these two clades are not well resolved [1, 6, 7].

Hitherto, the majority of the myxozoan phylogenetic studies have been based on the 18S ribosomal RNA (rRNA) gene (or SSU - small ribosomal subunit). Bartošová et al. [8] proposed that the 28S rRNA gene (or LSU - large ribosomal subunit) should be used as an additional reliable marker for inferring myxozoan phylogenetic relationships. However, since both rRNA genes are part of the rRNA cluster, they are not independent markers. It is known also that the different copies of the rRNA cluster do not always evolve under concerted evolution in animals, so that intra-individual variation can occur [9]. Indeed, the internal transcribed spacer (ITS) sequence, lying between the 18S rRNA and 28S rRNA genes, was found to possess two different copies co-existing in one myxozoan spore [10]. Because of these limitations of the rRNA cluster, there is a need for additional markers to reconstruct phylogenetic relationships within Myxozoa.

In Cnidaria, mt genomes can be linear or circular, and encode a conserved set of genes: 13 protein coding, 2 rRNA and 2 tRNA [11–14]. In addition, non-canonical protein-coding genes are also present in specific lineages [15, 16]. Gene order, amino acid substitutions and the general structure of the mt genome (linear versus circular) are useful characteristics to reconstruct phylogenetic relationships within bilaterian classes [3, 17, 18].

Complete mt genome sequences are known for four myxozoan species from the marine clade (*Kudoa septempunctata*, *K. hexapunctata, K. iwatai*, and *Enteromyxum leei*), one from the freshwater clade (*Myxobolus squamalis*) [19–21], and the myxozoan outgroup *P. hydriforme* [3]. These myxozoan mt genomes are extremely fast evolving, and contain only seven canonical mt genes that can be recognized unambiguously (*cob*, *cox1, cox2, nad1, nad5, rnl, rns*), except for *M. squamalis*, which seems to lack *cox1, cox2* and *cob* [20].

Surprisingly, these studies revealed important structural variation among myxozoan mt genomes. While in most myxozoans (e.g., *K. iwatai, K. septempunctata, K. hexapunctata*, and *M. squamalis*) the mt genome is encoded on a single circular chromosome, in *E. leei* the mt genome is partitioned into eight circular chromosomes. *Kudoa iwatai* harbors a non-coding mt plasmid along with the mt genome [21]. In *Henneguya salminicola*, a member of the freshwater lineage, the mt genome has been lost entirely, supporting the hypothesis that cellular respiration does not occur in this species [20]. Since cytochrome oxidase (*cox*) genes are also absent in *M. squamalis*, the only member of the freshwater lineage to have its mt genome sequenced, we question whether other members of this lineage have also lost their *cox* genes.

In spite of these structural differences, a few characteristics of the mt chromosomes are shared among most myxozoans, and their sister clade *P. hydriforme.* First, all tRNA genes are lost; second, a large non-coding region is present, ranging from 36 to 94% of the mt chromosome, with the coding genes clustered together. The mt genome of *M. squamalis* is the only known exception: its mt genome consists of 19% non-coding sequences, scattered among the coding regions.

In this study, we aimed to elucidate the evolutionary dynamics of mt genome structure and gene content among members of the freshwater clade. Our second goal was to evaluate the suitability of mt proteins as phylogenetic markers. Thus, we determined the complete mt genomes sequences of four members of the family Myxobolidae (*Myxobolus wulii, M. honghuensis*, *M. shantungensis*, and *Thelohanellus kitauei*) and one member of the family Sphaeromyxidae (*Sphaeromyxa zaharoni*).

## Results

### Characteristics of the new mt genomes

**Fig. 1:**
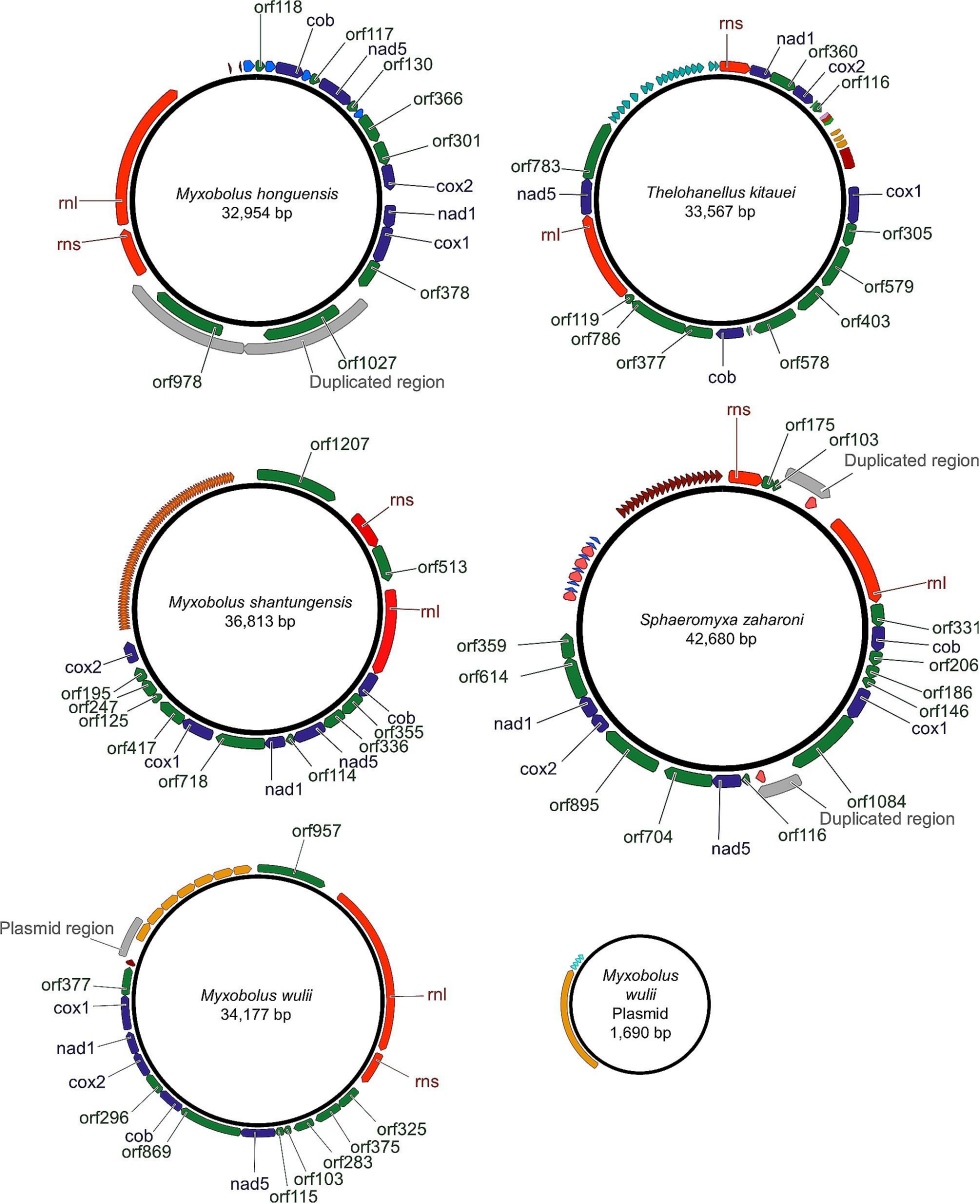
Genome maps of the myxozoan mt genomes obtained in this work The five canonical mt protein coding genes (*cox1*, *cox2, cob, nad1* and *nad5*) are in dark blue, the two rRNA subunits (*rns* and *rnl*) in red, unknown ORFs in green, and duplicated regions in gray. All other colored arrows indicate repeated elements. Repeated elements with the same sequence have the same color

Each of the five newly sequenced mt genomes is encoded on a single circular chromosome (Fig. 1). In addition, *M. wulii* harbors a non-coding mt plasmid of 1,690 base pairs (bp). The genome lengths range from 33.0 to 43.7 kbp with a GC-content of 19 − 34.5% (Fig. 1; Table 1). The GC content is similar in the four myxobolid species (19 − 23.8%) and is higher in *S. zaharoni*. The same pattern of low GC content is evident when only the seven canonical genes are considered (Table 1), suggesting that the GC content variation is not driven by repetitive elements present in non-coding regions. Interestingly, GC content higher than *S. zaharoni* is found in other non-myxobolid species, e.g., *Kudoa hexapunctata* (42.4%) and *K. septempunctata* (43.5%). Of note, such a large range of mt GC content was previously reported in other animal taxa, e.g., insects (22–45% [22]) and helminths (23 − 44% [23, 24]).

**Table 1:** Summary of mt genomic features

	*M. honghuensis*	*M. wulii*	*M. shantungensis*	*T. kitauei*	*S. zaharoni*
Mitochondrial genome size (bp)	32,954	34,177	36,813	33,567	42,680
Plasmid	none	1,690 bp	none	none	none
Canonical mt genes	*cox1, cox2, cob, nd1, nd5, rnl, rns*	*cox1, cox2, cob, nd1, nd5, rnl, rns*	*cox1, cox2, cob, nd1, nd5, rnl, rns*	*cox1, cox2, cob, nd1, nd5, rnl, rns*	*cox1, cox2, cob, nd1, nd5, rnl, rns*
%GC	19.0	19.9	21.0	23.8	34.5
%GC in canonical genes	19.1	21.7	22.0	25.3	37.8
Number of unknown ORFs	8	9	10	10	12
Total length of canonical CDSs (bp)	5,796	5,849	5,724	5,747	5,588
Total length of unknown ORFs (bp)	10,267	11,122	12,709	13,245	14,764
Total length of rRNAs (bp)	7,775	8,413	5,380	4,799	5,545
Total length of non-coding regions	9,116	8,793	13,000	9,776	16,783
Length of largest non-coding region (bp)	3,262	7,144	10,639	5,150	11,160
Fraction (%) of non-coding region out of the total genome size	27.7	25.7	35.3	29.1	39.3
Additional attributes	A tandem duplication led to the generation of two copies of an unknown ORF	The largest non-coding region includes tandem repeats	The largest non-coding region includes tandem repeats	A second non-coding region of 3,321 bp. Both non-coding regions include tandem repeats	The largest non-coding region includes tandem repeats, another non-coding region is duplicated

The *T. kitauei* mt genome that we sequenced in this work is different from scaffold03231 (accession JWZT01002463.1), a scaffold of the whole genome assembly of *T. kitauei* [25]. We have noted previously that this contig harbors mt genes [21]. A comparison between these two sequences (dot-plot and a sliding window similarity plot) is shown in additional file 1. These two sequences are almost identical across most of the alignment. However, unlike scaffold03231, the newly assembled mt genome of *T. kitauei* harbors neither duplicated genes nor pseudogenes. As our mt genome was obtained with a high coverage of both short and long reads (Additional file 2), we suggest that it better reflects the mt genome of *T. kitauei* than scaffold03231.

Out of the 13 canonical metazoan mt protein-coding genes, we identified only five in the mt genomes we sequenced: cytochrome c oxidase subunits I and II (*cox1–2*), cytochrome b (*cob*), NADH dehydrogenase subunits 1 and 5 (*nad1, nad5*). Similarly, we identified the two ribosomal RNA subunits (*rns* and *rnl*) in all sequenced genomes, but could not detect tRNA genes. All genes were predicted to be encoded on the same strand. All these characteristics are similar to those found in the *Kudoa* and *Enteromyxum* mt genomes [19, 21].

The codon usage of the five sequenced mt genomes was computed twice: for the five canonical mt protein coding genes and for all mt open reading frames (ORFs) including the five canonical mt protein coding genes (Additional file 3). In agreement with the observation that these sequences are AT-rich, the most abundant codons (when considering all ORFs) were TTT (Phe; 19.3, 13.6, 19.0, 13.2 and 10.0%, in *M. honghuensis, M. shantungensis, M. wulii, T. kitauei* and *S. zaharoni*, respectively), TTA (Leu; 10.6, 11.4, 8.9, 10.4 and 5.8%, respectively) and ATT (Ile; 7.7, 8.2, 8.1, 6.8 and 4.0%, respectively). Correspondingly, C- or G- rich codons showed the lowest frequencies. Remarkably, in *T. kitauei* no CGN arginine codons were identified in the mt canonical genes or in all other ORFs, in contrast to 152 arginine AGR codons that were present (Additional file 3b). This suggests a possible loss of all arginine CGN codons in this species. In *S. zaharoni*, there are no CGN codons in the mt canonical genes and only a single CGN codon (CGT) in *orf331*. This is the sixth codon after the N terminus and there is an ATG codon just after it, hence it is possible that this codon is never translated in *S. zaharoni*. In the other three myxobolid species, CGN codons are rare, thus we hypothesize that the few we detected are annotation errors. These results are in agreement with CGA, CGC and CGG codon losses described in *K. iwatai* [21].

### Unidentified open reading frames

**Fig. 2:**
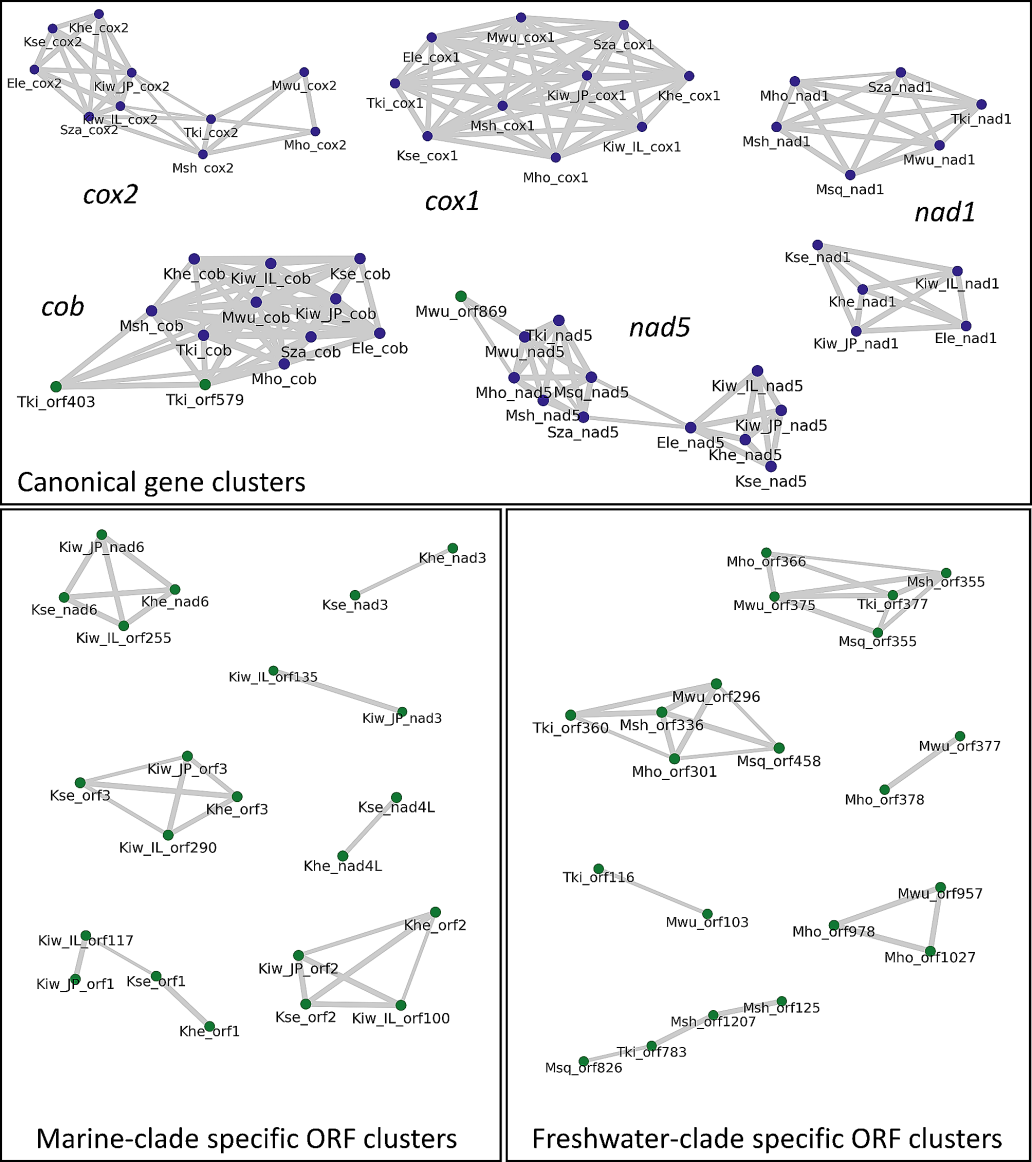
Results of homology-search between all ORFs encoded in myxozoan mt genomes Each node represents an ORF, and each edge connects two ORFs if the E-value, computed using BLASTp, between the two ORFs is < 10^− 5^. The thickness of the edge corresponds to different E-values: the thicker the line, the more significant the sequence similarity. Nodes in blue and green correspond to core mt proteins (*cox1, cox2, nad1, nad5* and *cob*) and unknown ORFs, respectively

In addition to the five canonical mt protein-coding genes mentioned above, in each species we found 8–12 unidentified ORFs longer than 100 amino-acids. These ORFs could not be aligned reliably to any canonical mt proteins, however all of these ORFs were found to be transcribed (Additional file 4). Given that cnidarian mt genomes typically encode 13 protein-coding genes, it is likely that at least some of these ORFs encode canonical mt proteins which could not be identified because of the high evolutionary rate that characterizes myxozoan mt genomes [19, 21]. Of these 8–12 unidentified ORFs, two have significant sequence similarity across the four myxobolid mt genomes sequenced (BLASTp E-value < 10^− 5^) and two were shared by three or fewer species (Fig. 2). Some of these ORFs were also identified in the previously sequenced mt genome of the myxobolid *M. squamalis* (MK087050) [20]. Sequence alignments of these ORF groups confirmed the presence of conserved blocks of amino-acids across myxobolid sequences (examples in Additional file 5). Interestingly, we identified putative transmembrane domains in several of these conserved myxobolid ORFs (Additional file 5).

### Non-coding regions and regions with repeats

Tandem duplication followed by gene loss is suggested to be the main mechanism leading to gene rearrangement within mt genomes [26–29]. Among our sequences, only *M. honghuensis* has a large tandemly duplicated region, which encompasses a single long ORF. All other genes in all other genomes were single copies. However, we found that some adjacent ORFs have partial sequence similarity. For example, in *M. wulii, nad5* and *orf869* share 175 bp in their 5’ regions (see Fig. 2 for BLASTp similarity). Similarly, in *T. kitauei*, the adjacent ORFs *orf403* and *orf479* share ∼ 500 bp with the 5’ end of the *cob* gene (see Fig. 2 for BLASTp similarity). This suggests that these ORFs encompass remnant DNA segments from past duplication events of core genes. A large non-coding region (∼ 1,800 bp) is duplicated in *S. zaharoni*, however, the two copies are apart indicating they did not originate from a tandem duplication event. In addition to duplicated regions, tandemly repeated elements ranging from 7 to 749 bp and repeated 7 to 76 times, are abundant in all mt genomes except *M. honghuensis*, which only contains four repeated elements spread among genes. These repeated elements form the major part of the non-coding regions in these five mt genomes (Fig. 1). Correspondingly, the length of the non-coding regions represented 27.7–39.3% of the total genome length, which is unexpectedly large for animal mt genomes (Table 1).

### Extreme rate of genome rearrangement

Gene order is extremely variable among the novel mt genomes with each species having a different gene order. The species with the most similar gene order are *M. honghuensis*, and *M. wulii*, which share the synteny block *(orf301*/*orf296*)-*cox2*-*nad1*-*cox1*-(*orf378*/*orf377*). Of note, *orf301* (*M. honghuensis*) and *orf296* (*M. wulii*) are orthologous, as are *orf378* (*M. honghuensis*) and *orf377* (*M. wulii*) (Fig. 2). Other cases of shared synteny between the newly sequenced species only involve pairs of genes, and could be the result of random rearrangements. Interestingly, no single gene-pair arrangement is shared between all sequenced myxobolids.

### Phylogenetic reconstruction

**Fig. 3:**
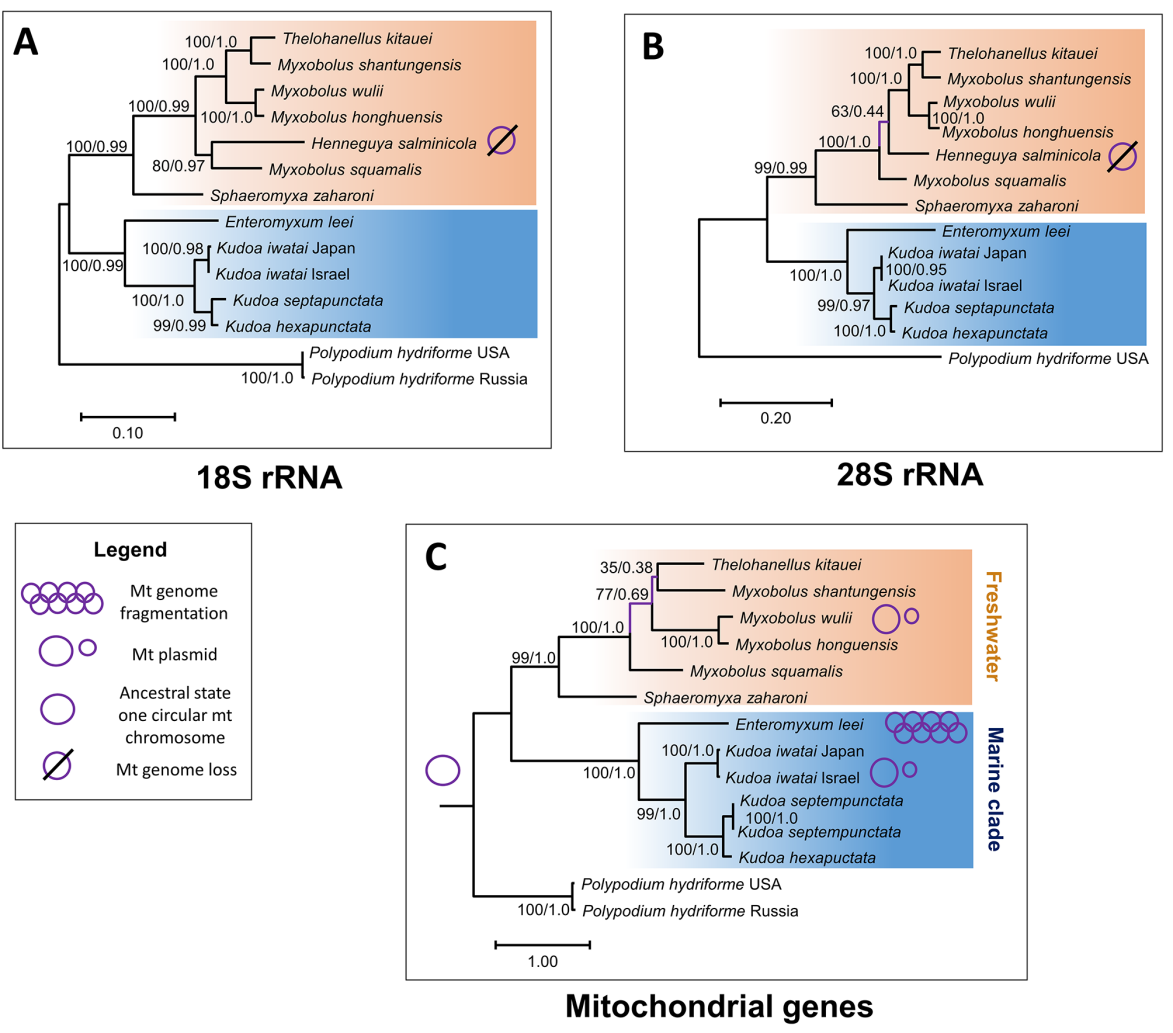
Phylogenetic relationships among Myxozoa Maximum likelihood trees reconstructed from (**A**) 18S rRNA sequences (14 taxa and 1,452 nucleotide positions) (**B**) 28S rRNA sequences (13 taxa and 2,772 nucleotide positions) and (**C**) mt protein-coding sequences (14 taxa and 839 amino-acid sites). The mt gene tree was inferred from the concatenation of the amino-acid sequences of five mt genes (*cox1, cox2, nad1, nad5* and *cob*). Branch lengths are proportional to the number of substitutions. ML bootstrap percentages [BS] / Bayesian probabilities [PP] that quantify branch supports are indicated next to the corresponding branch

The phylogenetic tree reconstructed with 18S rRNA sequences shows the highest branch support (Fig. 3a), and supports division of Myxosporea into a “freshwater” and a “marine” clade. In the marine clade, *E. leei* diverges first, and the monophyly of *Kudoa* is well supported with *K. septempunctata* and *K. hexapunctata* clustering together. Within the freshwater clade, *S. zaharoni* diverges first. Among myxobolids, salmon parasites *M. squamalis* and *H. salminicola* group together and diverge first. Among the remaining taxa, which are all cyprinid parasites, *T. kitauei* groups with *M. shantungensis*, while *M. wulii* and *M. honghuensis* are sister clades. In this tree, most branches have very high support (Maximum likelihood (ML) bootstrap support [BS] = 100, Bayesian posterior probability [PP] = 0.98–100) except for the branch grouping *H. salminicola* and *M. squamalis*, which is moderately supported (BS = 80, PP = 0.97). The ML and Bayesian trees reconstructed using 28S rRNA sequences agree with the 18S rRNA and also show high branch support, except for the relationships between *H. salminicola* and *M. squamalis*, which are not resolved (Fig. 3b). In the 28S rRNA trees, these two species are not grouped together, instead, *M. squamalis* is the first-diverging myxobolid lineage followed by *H. salminicola.* The mt ML and Bayesian trees agree with the rRNA trees regarding the evolutionary relationships within the marine clade (Fig. 3c). However, relationships among myxobolids are poorly resolved (of note *H. salminicola* could not be included in the mt tree because this species lacks a mt genome [20]). Specifically, the position of *M. squamalis* at the base of the myxobolid receives weak support (BS = 77; PP = 0.69) while the sister clade relationship of *T. kitauei* and *M. shantungensis* is not supported (BS < 50, PP < 0.5).

## Discussion

The goal of this work was to determine whether the loss of the mt genome and the resulting loss of aerobic cellular respiration observed in the myxobolid species *H. salminicola* [20] was widespread among myxosporean members of the freshwater clade. They are not: all myxobolid species sequenced in this work possess an mt genome, which encompasses *cox* genes (i.e., genes involved in the formation of complex IV, the complex which converts oxygen to water). This suggests that all these species generate ATP by using oxygen as the electron acceptor, and that the loss of aerobic respiration reported for *H. salminicola* is limited to this taxon, and perhaps also to *M. squamalis* for which we could not identify the *cox1* and *cox2* genes. Since *H. salminicola* and *M. squamalis* have been previously assigned to different myxobolid lineages (i.e., myxobolid subclades II and IV, respectively [30]), it is unclear if the loss of *cox1* and *cox2* genes occurred once or multiple times. According to Liu et al. [30], at least eight different subclades can be identified within Myxobolidae. Since the species sequenced in this work belong to distant lineages among the myxobolid subclade VIII, future sequencing efforts should target myxobolid species from clades I-VII to help elucidate the evolutionary steps that led to the loss of aerobic respiration in members of this group.

Each of the five freshwater myxozoans sequenced in this work has a circular mt chromosome. This supports the hypothesis that the ancestral myxozoan mt genome was a single circular chromosome. In *M. wulii*, as in *K. iwatai* [21], our DNA coverage analyses revealed the presence of a non-coding plasmid (i.e., non-coding minicircle). The possibility that these regions with high coverage represent nuclear mt copies (i.e., Numt) rather than non-coding plasmids is unlikely since: (1) their coverage is higher than the coverage of the nuclear and the mt genomes together, and (2) we validated their presence by PCR using primers that are outward facing on the mt chromosome but inward facing on the plasmid DNA (results not shown). While non-coding mt plasmids are uncommon in Metazoa, next-generation sequencing technologies may lead to the discovery of additional cases.

Recombination between repeated elements is probably the mechanism for the origin of mt minicircles as described, for example, in the nematode *Meloidogyne javanica* [31] and the wasp *Conostigmus* sp. [32]. The fact that these minicircles encompass the origin of replication may explain why they are present with high frequency, since they can replicate independently and faster than the rest of the mt genome because of their small size [32]. It is interesting that the presence of such plasmids could represent the first step toward a partitioned genome such as observed in *E. leei*. Indeed it has been suggested that the coexistence of minicircles along the mtDNA could be the first step toward further fragmentation of mtDNA in lice [33].

We could identify only five canonical protein-coding genes within myxozoan mt genomes [20, 21]. This observation supports the following alternative hypotheses: (1) Myxozoa have lost the remaining protein-coding genes [19]. Under this hypothesis, mt protein complexes may be functional without essential proteins, e.g., the cytochrome oxidase complex without COXIII. It is also possible, although unlikely, that another protein complements COXIII. (2) These missing genes were transferred to the nuclear genome. However, no support for this hypothesis was found, as we searched for the missing proteins in myxozoan nuclear genome and transcriptome assemblies without success (data not shown). (3) These genes are present, but cannot be recognized due to the extreme rate of sequence evolution of these genomes [15]. Based on the third hypothesis, some of the unannotated ORFs present in myxozoan mt genomes may be the ‘missing’ canonical protein-coding genes. In support of this hypothesis, the genes that could be identified are among the longest and most conserved among the 13 canonical mt protein coding genes [34, 35]. However, it is possible that the unannotated ORFs may serve other yet undiscovered functions.

Several observations support the hypothesis that the unannotated ORFs are functional. First, we showed that these regions are transcribed. However, since mt genomes are usually transcribed on large polycistronic transcripts that encompass most of the mt genome, the evidence of transcription does not prove functionality. As a case in point, repetitive elements were also found to be transcribed (Additional file 4). Second, they are shared between myxobolid genomes, with common amino-acid blocks (Additional file 5), which suggests that they evolved under purifying selection (by comparison, no similarity could be found among mt non-coding regions of myxbolids, which harbor different repeated elements). Third, the presence of transmembrane domains in some of these shared ORFs (Additional file 5) supports the view that they could be genes that encode membrane proteins involved in the electron transport chain. Further studies are needed to better characterize the function of these myxobolid mt ORFs, e.g., proteomics work to validate the presence of these proteins. In particular, microscopic labeling methods could validate their cellular localization to the mitochondria [36].

The presence of tRNA genes was noted in the first published mt genomes of Myxozoa. Specifically, Takeushi et al. [19], annotated four and one tRNA genes in the mt genomes of *K. hexapunctata* and *K. septempunctata*, respectively, but not in the other two *Kudoa* species they studied. Lavrov and Pett [15], however, suggested that these tRNAs are annotation errors. In agreement with Lavrov and Pett [15], no mt tRNAs have been identified in the mt genomes of *E. leei* and *M. squamalis* [20, 21], nor in any of the species studied here. We thus conclude that tRNA genes are absent from myxozoan mt genomes. Since tRNAs are also absent from the mt genome of *P. hydriforme* [3], the loss of mt tRNA genes is likely an ancestral characteristic in Endocnidozoa (i.e., the clade encompassing Myxozoa and *P. hydriforme*).

The sequenced mt genomes analyzed in this work include non-coding regions harboring tandem repeats. This characteristic was found also in the sister clade of Myxozoa, *P. hydriforme* [3]. While the mt genome of most animals is very compact and contains only short noncoding regions, long-read sequencing enabled the discovery of several similar cases of mt tandem repeats in both parasitic and non-parasitic animals, e.g., parasites: the parasitoid wasp *Nasonia vitripennis* [37], the cestode *Echinococcus granulosus* [38], the fluke *Shistosoma haematobium* [39]; free-living species: beetles of genus Dynastes [40], the stink bug *Erthesina fullo* [41], and frogs of genus *Breviceps* [42]. Further research is needed to elucidate how these regions are formed and whether there are any selective forces preventing them from being deleted.

While the amplification of 18S rRNA sequence is straightforward in Myxozoa using universal primers [43–45], the sequencing of complete mt genome in this group is complex and hampered by the presence of numerous repetitive elements, which required the use of different sequencing approaches (short and long reads). The fast rate of evolution of the mt encoded proteins, and the case of mt-genome loss hamper their utility as informative phylogenetic markers. Indeed, a few relationships within Myxobolidae were not resolved in our mt tree. However, the fast rate of evolution of mt genomes may be an advantage for developing mt-based markers at the species level, for example to identify cryptic species or to confirm the phylogeographic origin of invasive myxozoans, e.g., *Tetracapsuloides bryosalmonae*, the causative agent of proliferative kidney disease [46, 47].

Prior to this study, the mt genomes of *T. kitauei* and *M. honghuensis* could not be assembled successfully [25, 48]. To understand this discrepancy, we first checked whether mt reads were present in the published raw sequence data: we mapped their reads to our present mt genome assembly. We found that these older data have high mt coverage, which is higher than the observed nuclear coverage, as expected. These results suggest that differences in coverage between mt and nuclear genomes may bias some assembly programs [49]. We emphasize the importance of alternative sequencing approaches and assembly tools when reconstructing mt genomes, and we found programs such as Novoplasty [50] and MitoHifi [51] useful.

## Conclusions

Our study supports the hypothesis that the ancestral myxozoan mt genome was encoded on a single circular chromosome. We show that five canonical mt genes and two rRNAs are shared among marine and freshwater myxozoans, suggesting that these genes also characterized the ancestral myxosporean mt genome. We speculate that the unidentified ORFs are translated to genuine proteins. Finally, the loss of the mt genome and aerobic respiration described in *H. salminicola* is not widespread among myxobolids.

## Materials and methods

### Sample collection

*Sphaeromyxa zaharoni* plasmodia were obtained by Prof. Arik Diamant from lion fish (*Pterois miles*) from the Red Sea (Eilat, Israel) on January 2016 (permit number 2014/40517). *Myxobolus wulii* and *M. honghuensis* plasmodia were collected from gibel carp (*Carassius auratus gibelio*) from cultured ponds in Yancheng city (Jiangsu province, East China) in June 2017 and October 2017, respectively. *Thelohanellus kitauei* plasmodia were obtained from common carp (*Cyprinus carpio*) from cultured ponds in Zhijiang country (Hubei province, Central China) in July 2017. *Myxobolus shantungensis* (infecting the gill arch of the common carp *C. carpio*) was collected from the Willamette River (Oregon, USA) by the Department of Fisheries & Wildlife in May 2013.

Myxozoans were identified by their fish host, tissue tropism and myxospore morphology. In addition, we verified that the 18S rRNA sequence of each sample (see below for 18S rRNA sequence annotations) was either identical or almost identical (> 99% identity) to the sequences available in public databases for the same species (i.e., *M. honghuensis* KJ725074.1; *M. shantungensis* KJ725079.1; *M. wulii* HQ613412.1; *S. zaharoni* AY538662.1 and *T. kitauei* KU664643.1). Of note, the first 25 bp of *M. shantungensis* KJ725079.1 were removed from the comparison since they did not align with any 18S rRNA sequence and probably represent sequencing errors.

### Genomic DNA extraction and Illumina sequencing

DNA extractions from cysts were performed with the DNeasy Blood & Tissue Kit (Qiagen) according to manufacturer’s instructions. Illumina sequencing of *T. kitauei, M. wulii* and *M. honghuensis* was performed by the Technion Genome Center (Haifa, Israel). Libraries were build using the DNA NexteraXT/TruSeq sample preparation protocol, and the sequencing of 100 bp paired-ended reads was performed on a HiSeq2500 machine.

Sequencing and library preparations of the *M. shantungensis* sample were performed by the Center for Genome Research and Biocomputing (CGRB) at Oregon State University (Corvalis, OR). The library was built with the PrepX Complete ILMN DNA Library Kit. Paired-end sequencing with 150 bp reads derived from fragments of average length ∼ 350 bp was performed on a HiSeq3000 platform. For *S. zaharoni*, we used published reads (Genbank accession SRX1034914) [2].

### Illumina data assembly and preliminary mt assembly

For all Illumina samples, the removal of adapters was performed using Cutadapt version 2.10 [52]. The quality of the reads was then verified with FastQC version 0.11.8 [53]. *De novo* assembly of the DNA reads was performed with the IDBA-UD assembler (v 1.1.1) [54] with default parameters for all species.

The *cox1* and *nad1* amino-acid sequences of *K. iwatai* (BAR94716.1, BAR94710.1) and *E. leei* (CRX66571.1, CRX66578.1) were used as query in tblastn searches against databases composed of the contigs assembled with IDBA-UD. The *cox1* and *nad1* ORFs were extracted from the best-hit contigs. These ORFs were then used as “seed” sequences for the seed-and-extend algorithm implemented in the script NOVOplasty version 2.7.1 [50]. Of note, all myxozoan mt protein coding genes were originally tested, but the best results were obtained with *cox1* and *nad1*. The selected NOVOplasty parameters were: “mito” mode, read length of 100 (or 150 for *M. shantungensis*). To account for the putative presence of unusual genome architectures, the genome range parameter was set to between 12,000 and 100,000 bp. All other options were kept to default setting. Depending on the sample, 1 to 150 putative mt contigs were assembled by NOVOplasty (Contigs_1 output file). Because some of these contigs overlapped, they were then assembled using the default assembler of Geneious Pro version 11.1.5 (Biomatters) under default parameters. After assembly, the Geneious contigs were elongated by mapping reads to these sequences. Indeed, setting a minimum overlap of 25 bp between a read and the mt contig allowed us to extend the sequence by 75–125 bp, since the reads are 100–150 bp long. Reads mapping were repeated until the sequences could be assembled into a circular sequence except for *S. zaharoni* and *T. kitauei* for which this approach failed. For these two species Oxford Nanopore Technology (ONT) reads were obtained (see below).

For all species, read mapping was unambiguous in gene coding regions. In these regions, the coverage was homogeneous and without any drops (indicating assembly errors) or peaks (indicating the presence of repeats). This was not the case for non-coding regions, in which the mt genome was found to harbor repeated elements (i.e., minisatellites and/or distant repeated regions). These elements were detected by sharp increase in coverage along the mt sequence using the option “Map multiple best matches: To all”. The first step was to identify all types of repeated elements. We next determined their copy number. To identify repeats we used the Repeat Finder plugin version 1.0.1 implemented in Geneious Prime version 2023.2.1 (Biomatters) with the parameters “Minimum repeat length 50; Maximum mismatches 5%; exclude repeats up to 50 bp longer than contained repeat; exclude contained repeat when longer repeat has a frequency of at least 2; Maximum repeats (approximate) to find 200”. Following the approach used by Novosolov et al. [3], the number of repeats was determined by adding repeats until the coverage in the repeated region was similar to the coverage in the coding region (using Geneious Prime with the option “Map multiple best matches: Randomly” and default mapping parameters otherwise). Of note, this approach did not allow determining the exact sequence of each repeat. Instead, each repeat is a consensus of the different repetitive elements of the same type.

These approaches allowed us to obtain the complete circular mt genome sequences for *M. wulii, M. honghuensis* and *M. shantungensis.* As quality control we verified that all contigs assembled by NOVOplasty could be mapped to the circular mt consensus of each species, suggesting that no alternative chromosome topology exists. However, a few regions with low read coverage remained (see below).

### Low-coverage regions in *M. honghuensis* and *M. wulii*

For *M. honghuensis*, low coverage was observed in the mt genome region between 32,150 and 32,900 bp (Additional file 2 A). This region encompasses a palindrome. Unfortunately, we were unable to amplify and sequence this region (four primer combinations were tried) probably because of the secondary structure created by the palindrome. The secondary structure probably explains the lower coverage in this region. The assembly of this region, originally obtained with NOVOplasty was performed again manually by performing several rounds of read mapping with Geneious Prime, starting from the flanking regions. The procedure was performed more than twice using different mapping parameters. All mapping analyses led to the exact same sequence, and no alternative assemblies could be identified.

For *M. wulii*, higher coverage values than the rest of the genome were found in the region between 27,230 and 28,940 bp (Additional file 2 C). While repeated elements flank this region, the center of this region does not appear to be duplicated or to be composed of repeated elements. Following a similar observation in *K. iwatai* [21], we assumed that this region may be part of a plasmid. To test this hypothesis, outward facing primers were designed in the 5’ and 3’ ends of this high coverage region. The PCR products obtained using these primers allowed us to confirm that this region could be circularized into a minicircle of 1,690 bp. Remarkably, this minicircle was found to be entirely non-coding (Fig. 1).

### Genomic DNA extraction and ONT sequencing

For *S. zaharoni*, 1.5 µg of the extracted DNA was used for library preparation following the protocol detailed in the “Ligation sequencing Kit 1D” for the MinION Mkb1 device using SQK-LSK108 kit. The final obtained library concentration was 4.36 ng/µl (measured using a Qubit fluorometer). Sequencing was performed in the MinION Mk 1 R9.4 flow cell for 48 h using MinKNOW software (V18.12.6.0). The ONT sequencing yielded 186,104 reads for *S*. *zaharoni*.

ONT sequencing of *T. kitauei* was performed by HyLabs laboratories (Rehovot, Israel). For library preparation, 400 µg of DNA was used and measured using the Qubit fluorometer. The library was prepared as detailed in the “Rapid Barcoding Sequencing” protocol version RBK_9054_V2_revD_23Jqn2018 using the “Rapid Barcoding kit”. The flow cell MK 1 R9 was connected to a MinION Mkb1 device and sequenced 48 h using MinKNOW software (V3.1.19) for retrieving the sequencing data. The ONT sequencing yielded 375,139 reads for *T. kitauei*.

### ONT data assembly of *T. kitauei* and *S. zaharoni*

To correct and assemble the ONT reads, Canu (version canu-1.8) [55] was executed with parameters: “correctedErrorRate = 0.2 stopOnLowCoverage = 0 corOutCoverage = 1000 contigFilter="2 0 1.0 0.5 0"”. The “genomeSize” parameter was set to 102 M for *S. zaharoni* and 150 M for *T. kitauei*, based on preliminary genome-size estimations. To identify mt contigs within the Canu assemblies, BLASTn searches were conducted using the mt contigs assembled by NOVOplasty as query.

For *S. zaharoni*, one Canu contig (44,476 bp long), which could be circularized, included all the NOVOplasty contigs. Since the ONT sequencing has a high sequencing error rate, this contig was corrected by mapping the Illumina reads using Geneious Prime. The number of repeats present in a repeated region was set so that the coverage of the repeated region matches the coverage of the coding region as described above. After read mapping, this contig could be circularized to form one large chromosome of 43,298 bp.

Among the *T. kitauei* contigs, one contig from the Canu assembly (31,021 bp long) harbored all seven canonical mt genes (*cob, cox1, cox2, nad1, nad5, rnl* and *rns*). This contig could not be circularized as one region with repeats was poorly assembled. To resolve this region, ONT reads overlapping this region were aligned and the resulting alignment manually curated. Next, we corrected the obtained consensus using mapping rounds of Illumina reads. After mapping, the contig could be circularized to form one large chromosome of 33,761 bp.

### Final base-coverage analyses

After completing the assemblies, the sequencing depth was computed by mapping Illumina and ONT reads against each mt sequence with the software Geneious Prime version 2023.2.1. The following parameters were used for mapping the Illumina reads: “Custom Sensitivity; Map Multiple best matches Randomly; Only map paired reads which map nearby; Minimum mapping quality not select; Trim paired read overhangs selected; Minimum support for structural variant discovery 2 reads; include insertion and structural variant not select; Allow Gaps Maximum per Read 5%; Maximum Gap Size 10; Minimum Overlap 90; Minimum Overlap Identity 95%; Word Length 50; Index Word Length 13; Maximum Mismatches Per Read 5%; Maximum Ambiguity 64”. The mapping of the Nanopore reads was performed using default parameters of the sensitivity option: “Medium sensitivity/Fast”. The read mapping analyses confirmed the circularity of all mt chromosomes. The results of the final base coverage analyses using Illumina and ONT reads are presented in additional file 2.

### Genome annotation and sequence analysis

For each mt genome assembled, the first step in the annotation was the identification of ORFs with the “Find ORFs” tool of the program Geneious Prime version 2023.2.1. Parameters were: “Minimal ORF length 300, Genetic code 4 (Mold, Protozoan and Coelenterate Mitochondrial), ORF start codon to use: ATG and alternative initiation codons (GTG, CTG, ATT, TTA, TTG, ATA, ATC)”. After this step, a gene annotation was performed with the MITOS2 webserver [56] (using “translation Table 4”). Each ORF with an annotation was then aligned with orthologous myxozoan sequences. Start and end of protein coding genes and unknown ORFs were chosen based on four criteria: limiting overlap between genes, reducing the length of non-coding regions, maximizing similarity between orthologous myxozoan sequences, and limiting the use of non-standard start codon. Finally, putative transmembrane domains were searched for using the plugin “Predict Transmembrane Regions version 1.0.2” implemented in Geneious Prime. ORFs present on the reverse strand, ORFs enclosed within another ORF or gene, ORFs encompassing entire repeated regions, as well as ORFs enclosed within repeated regions that included more than two repeated elements, were not considered.

Because only five protein-coding genes could be identified using the MITOS2 webserver [56] (last accessed Jan 2023), following [3], we used hidden Markov models implemented in the HMMER3 program version 3.1b2 [57, 58], to broaden our mt gene searches. We were unable to identify additional mt genes following these searches.

The two rRNA genes (*rnl* and *rns*) were first annotated automatically using the MITOS2 webserver [56]. To confirm their location in *T. kitauei* we followed the approach of Takeushi et al. [19] and mapped RNA reads deposited under SRR1103279 [25] (Additional file 4) to the mt genome of this species. Of note, these RNA data seem to be enriched in RNA genes when compared to SRR17152144 & SRR17152863 [59], which were used to evaluate transcription level (see below). Read mapping was performed using Geneious Prime version 2023.2.1 under the “Medium-Low Sensitivity / Fast” mapping sensitivity and default settings for all other parameters. The two regions with high RNA coverage indicated the position of the rRNA genes. For all species, the rRNA boundaries were determined visually based on sequence alignment of the rRNA region identified by MITOS2 with the rRNA genes of *M. squamalis*. The sequences were aligned with MAFFT version v7.490 [56] as implemented in Geneious Prime under the L-INS-i algorithm.

The webservers tRNAscan-SE [60] (last accessed Jan 2023), ARWEN [61] (last accessed Jan 2023) and MITOS2 [56] (last accessed Jan 2023) were used for tRNA identification. The structures detected by these programs were then inspected manually. They were all found to be false positive hits (i.e., they either lacked a canonical secondary structure or overlapped with an annotated protein or rRNA gene).

The final annotation of the repeat regions was done following Novosolov et al. [3]. We used the Repeat Finder plugin version 1.0.1 implemented in Geneious Prime for initial repeat identification (see above). Dotplots were then used for the identification of the boundaries of the different repeated elements. For each type of element identified, a multiple sequence alignment was computed. A consensus sequence was then computed from the resulting alignment. These consensus sequences were used to annotate the mt genomes using the “live annotate and predict” tool of Geneious Prime (with a similarity threshold of 75%).

### Sequence similarity search among myxozoan ORFs

The program blast + version 2.14.0 [62] was used to search for remote homology between each of the proteins encoded within the myxozoan mt genomes. Next, the Python library NetworkX [63] was used to represent the results as a graph (Fig. 2).

### rRNA read mapping to confirm the transcription of myxozoan ORFs

To support the hypothesis that the ORFs identified in the myxobolid genomes are functional, we downloaded the following RNA reads obtained by other groups: SRR17152144 & SRR17152863 (*T. kitauei*) [59]; SRR16897929 (*M. honghuensis*) [48] and SRR17138786 (*M. wulii*) [64]. Read mapping was performed with Geneious Prime version 2023.2.1 under the “Medium-Low Sensitivity / Fast” mapping sensitivity and default settings for all other parameters. Of note, because the RNA samples originated from different specimens than the DNA obtained in this study, regions of low coverage could reflect either a low level of transcription or artifacts stemming from sequence differences between the RNA reads and the DNA, e.g., due to the presence of point mutations.

### Codon usage

Codon usage in the five mt genomes was computed using Geneious Prime version 2023.2.1 on all ORFs, canonical and non-canonical (Additional file 3).

### Nuclear rRNA sequences

Novel complete sequences of the nuclear rRNA cluster (i.e., 18S rRNA, ITS1, 5.8S rRNA, ITS2, 28S rRNA) of each species were assembled from raw Illumina reads, either generated in this study, or published: SRR7754567 [2], SRR7760053 [20], SRR2034739 [2], and SRR1576776 [20] were used to assemble the rRNA cluster of *H. salminicola, M. squamalis, E. leei*, and *K. iwatai*, respectively. Specifically, rRNA sequences were identified in the IDBA-UD assembly. Several rounds of read mapping were performed on the sequences until convergence of the consensus sequence was obtained. Final mapping of Illumina reads used the following parameters: Custom Sensitivity; Trim paired read overhangs; Only map paired reads which map closely nearby. Minimum support for structural variant discovery 2; Allow Gaps Maximum per Read 3%; Maximum Gap Size 3; Minimum Overlap 75; Minimum Overlap Identity 97%; Word Length 18; Index Word Length 13; Maximum Mismatches Per Read 3%; Maximum Ambiguity 4. Other options (e.g., accurate map reads with errors to repeat regions, search more thoroughly for poor matching reads) were not chosen. To save computation time we used a fraction of the reads in the mapping analyses. Specifically, the final coverage was ∼100 - ∼900 depending on species, and we verified manually that coverage was homogeneous along the sequence (without drops, which would have suggested assembly errors).

We also assembled the rRNA cluster of *P. hydriforme*, but in this case we used the ONT reads deposited under SRX14223719, and performed mapping using the “Medium Sensitivity / Fast” setting of Geneious. The mean coverage of the final mapping was 628.8 ± 23.4.

The consensus sequence of the rRNA cluster of *K. hexapunctata* was assembled from sequences AB693042, AB902954, AB902955, AB902956, AB902957, LC200462, LC200463, LC200464, LC200465, LC200466, LC200467, LC200468, LC200469, LC200470, LC200471, LC200472, LC200473, LC200474, LC200475, LC200476, LC200477, LC381991, LC381992, LC381993 [65–68].

Similarly, the consensus sequence of the rRNA cluster of *K. septempunctata* was assembled from sequences AB553293, AB617628, AB617629, AB643791, AB643792, AB643793, AB643794, AB643795, AB643796, AB643797, AB693040, AB731754, AB731755, JQ302299, LC028894, LC037195, LC037196, LC037197, LC037198, LC037199, LC037200, LC037201, LC037202, LC128640, LC128641, LC128642, LC128643, LC616853, LC616854 [19, 65, 69–75]. Finally, the sequence LC066366 [76] was used to represent *K. iwatai* from Japan.

For all consensus sequences, the start and end of the rRNA genes were determined using sequences search against the Rfam database [77] available at https://rfam.org/search#tabview=tab1 (last accessed 29 May 2023).

### Sequence alignments

Three phylogenetic datasets were analyzed: 18S rRNA, 28S rRNA, and mt. The 18S rRNA and the 28S rRNA datasets were based on the genes identified from the rRNA cluster sequences obtained as described above. The mt alignment was based on the amino-acid sequences of the genes *cob*, *cox1*, *cox2*, *nad1*, and *nad5*: *E. leei* (LN868201-7); *K. hexapunctata* (LC009437); *K. iwatai* (LC009438, Japan); *K. iwatai* (LT671462, Israel); *K. septempunctata* (LC009436); *K. septempunctata* (AB731753); *M. squamalis* (MK087050); *P. hydriforme* (MN794187); and from this study: *M. honghuensis*; *M. shantungensis*; *M. wulii*; *S. zaharoni* and *T. kitauei*. Each protein-coding gene was aligned separately (see below) and then the five genes were concatenated in a single super matrix

The amino-acid and nucleotide (for rRNAs) sequences were aligned using MAFFT [56] as implemented in the Guidance 2 webserver version 2.02 [78]. The parameters for the mt alignments were: “100 bootstrap --maxiterate 1000 --localpair”. Unreliable column positions (i.e., positions with a reliability score below 0.93) were excluded, as well as columns that included > 50% gaps. The alignments are available in Additional files 6–8.

### Phylogenetic analyses

Phylogenetic trees were reconstructed for the concatenated mt dataset and for each rRNA dataset using both ML and Bayesian approaches. The mt ML tree was reconstructed using IQ-TREE 2.2.2.6 [79] using model finder plus [80] and 1,000 non-parametric bootstraps (option: -m MFP -b 1000). The evolutionary models were chosen based on the corrected Akaike Information Criterion and were MTZOA + F + I + G4, GTR + F + G4, and the GTR + F + I + R3 for the mt protein-coding genes, the 18S rRNA and the 28S rRNA datasets, respectively. Bayesian phylogenies were inferred with the program Phylobayes-MPI-1.8 [81], under the CAT-GTR mixture model. For each dataset four chains were run in parallel for 95,000 iterations. Burn-in was set to 5,000 trees, and trees were sampled every 10 iterations. Convergence was assessed using the bpcomp and tracecomp programs (from the PhyloBayes package [81]) and visually by plotting the value of each parameter from the “.trace” files as a function of the number of iterations. Specifically, we verified that each parameter reached a plateau before the burnin threshold and that, for each of the three analyses, the maximum discrepancy (max_diff) observed across all bipartitions was below 0.05. Similarly, we verified that for all parameters the discrepancies (rel_diff) were below 0.15 and the minimum effective size (effsize) greater than 300 at the end of the run. Following Novosolov et al. [3] and Chang et al. [2] *P. hydriforme* was used to root the phylogenetic trees. Of note, we do not expect long-branch attraction or saturation to substantially affect the reconstructed tree because: (a) we employed models including CAT-GTR, which are known to minimize the chances of such phenomena [82]; (b) for the mt genes, we analyzed also amino-acid characters, which are less susceptible to saturation.

### Electronic supplementary material

Below is the link to the electronic supplementary material.

Supplementary Material 1

Supplementary Material 2

Supplementary Material 3

Supplementary Material 4

Supplementary Material 5

Supplementary Material 6

Supplementary Material 7

Supplementary Material 8

## Data Availability

The Illumina read data and ONT reads have been deposited in the European Nucleotide Archive under study ID numbers PRJEB53883– PRJEB53887. The mtDNA sequences were deposited under accession numbers OY743230–OY743235. The sequences of the rRNA clusters were deposited under accession numbers OY751525–OY751528 and OY756915-OY756919.

## Abbreviations

bp: base pairs
BS: bootstrap support
*cox1-2*: cytochrome c oxidase subunits I and II
*cob*: cytochrome b
ITS: internal transcribed spacer
ML: maximum likelihood
mt: mitochondrial
*nad1-5*: NADH dehydrogenase subunits 1–5
ONT: Oxford Nanopore Technology
ORF: open reading frame
PCR: polymerase chain reaction
PP: Bayesian posterior probabilities
