# Supplementary material for: Evolution of myxozoan mitochondrial genomes: insights from myxobolids
Source: BMC Genomics. 2024 Apr 22;25:388. doi: 10.1186/s12864-024-10254-w (PMC11034133; doi:10.1186/s12864-024-10254-w)
Supplement: Supplementary file 1 — Supplementary Material 1 [file 12864_2024_10254_MOESM1_ESM.docx]

Thelohanellus kitauei (start position15835)

JWZT01002463 (reversed)

0

10 kb

20 kb

30 kb

40 kb

10 kb

20 kb

30 kb


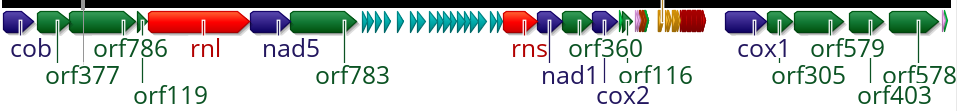


**A**


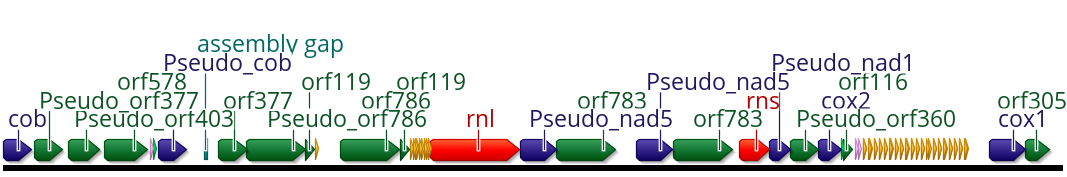


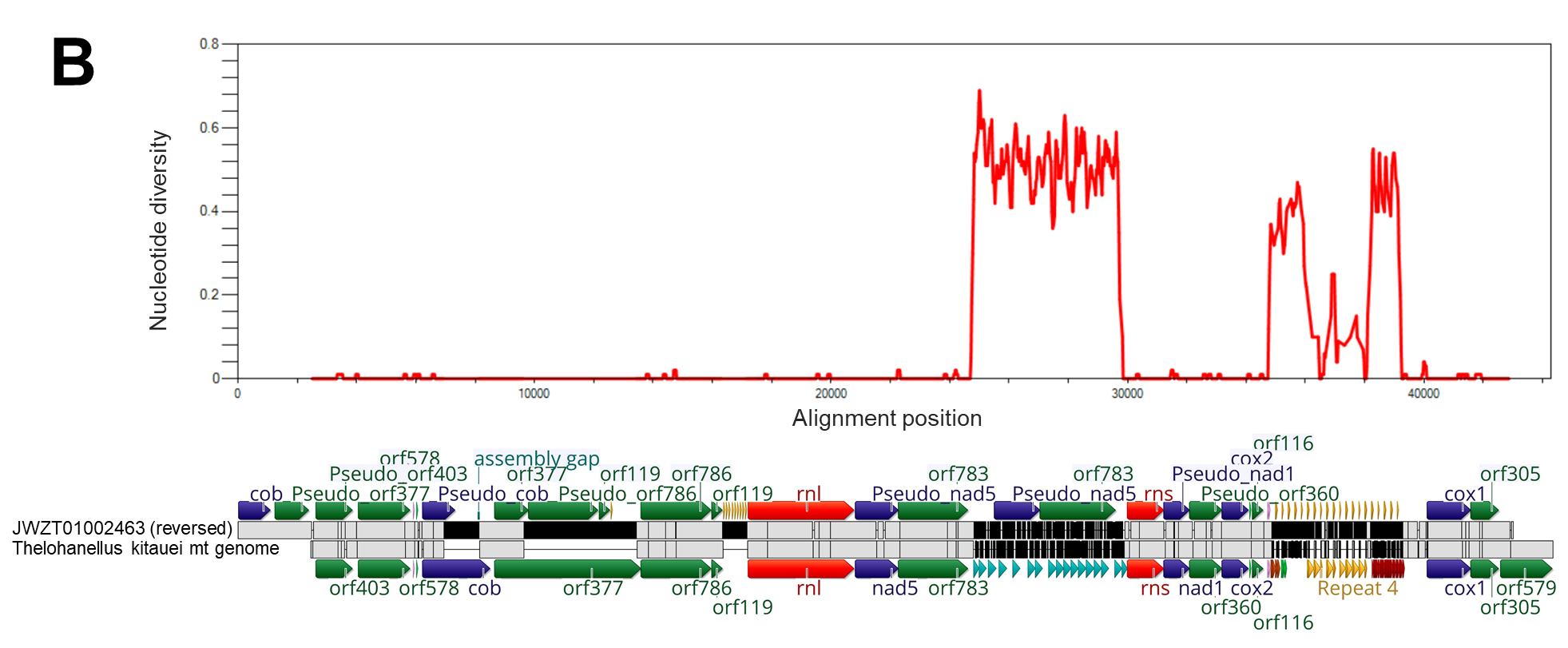


**Additional file 1 Comparison of the mitochondrial sequence of *T. kitauei* with scaffold03231 (accession JWZT01002463.1, X axis).**

A) Dot-plot analysis. Y axis, the mitochondrial sequence of *T. kitauei* (start position set to 15835 of the published sequence); X axis, scaffold03231. Short matches (below 100 bp) on the direct and reverse complement are indicated in blue and green, respectively. Long matches over 100 bp, on the direct strand, are indicated in red. Correspondingly, the high similarity between the direct strands of both sequences is indicated by the red diagonal lines. The annotations of each chromosome are indicated near the corresponding axes. B) Sliding window plot of nucleotide diversity (π) across the alignment of the mitochondrial sequence of *T. kitauei* with scaffold03231. Window length: 100 bp, step size: 25 bp, sites with gaps were assigned π=0. The sequence alignment is provided below the plot, conserved and variable positions are indicated in gray and black, respectively. Protein coding genes, ORFs, and rRNAs are indicated by dark blue, dark green, and red arrows, respectively. All other arrows represent repetitive elements. The prefix *pseudo* indicates cases in which homologous regions in scaffold03231 contain stop codons.
