# Supplementary material for: Evolution of myxozoan mitochondrial genomes: insights from myxobolids
Source: BMC Genomics. 2024 Apr 22;25:388. doi: 10.1186/s12864-024-10254-w (PMC11034133; doi:10.1186/s12864-024-10254-w)
Supplement: Supplementary file 2 — Supplementary Material 2 [file 12864_2024_10254_MOESM2_ESM.docx]

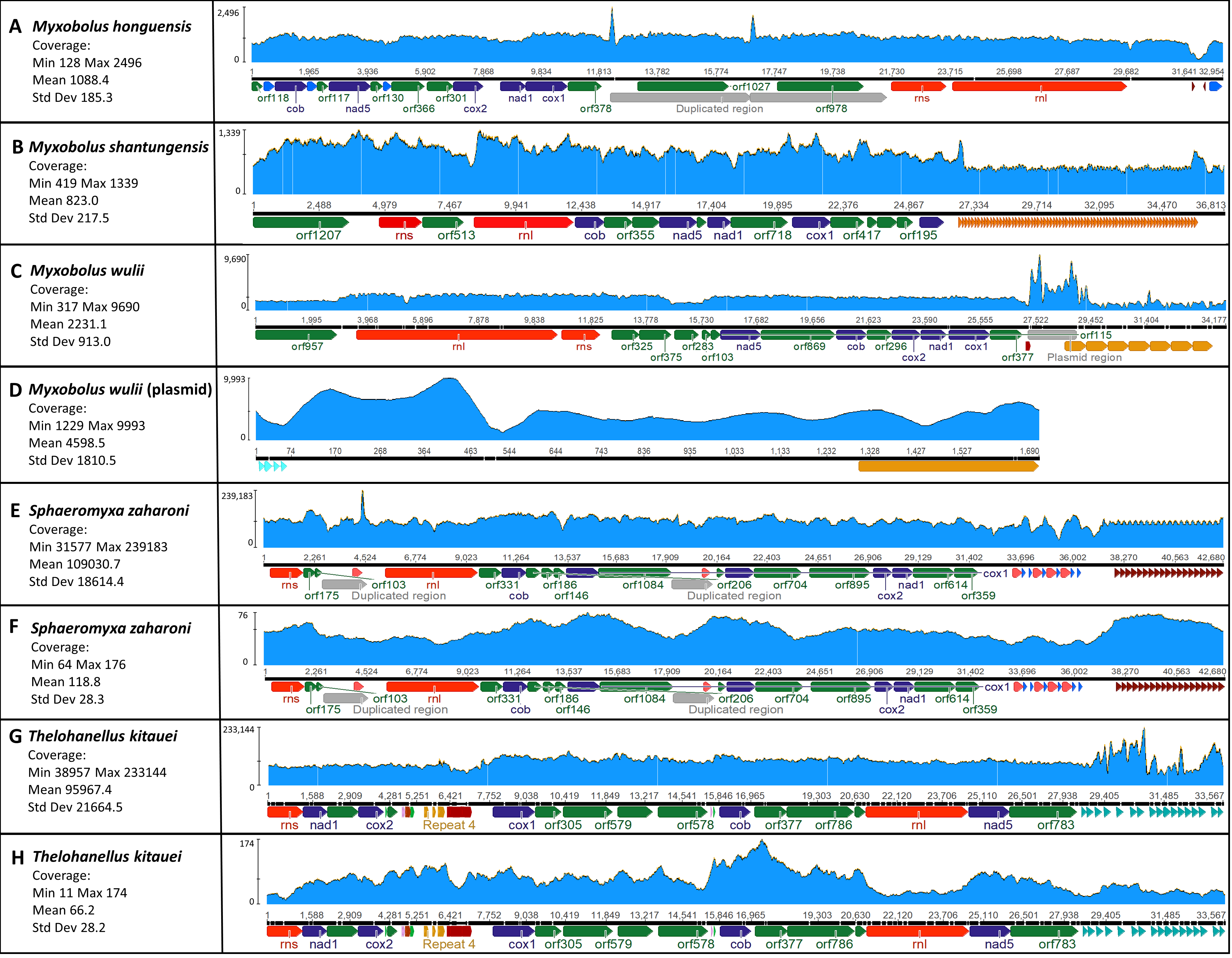


**Additional file 2 – DNA coverage depth of the mt chromosomes.**

Illumina reads (or ONT reads) were mapped to each chromosome individually using Geneious Prime version 2023.2.1 (see Methods). A) *M. honghuensis* Illumina reads; B) *M. shantungensis* Illumina reads; C) *M. wulii* Illumina reads; D) *M. wulii* plasmid Illumina reads; E) *S. zaharoni* Illumina reads; F) *S. zaharoni* ONT reads; G) *T. kitauei* Illumina reads; H) *T. kitauei* ONT reads. Each species sequence is shown schematically below the per-base coverage, indicated in light blue. The sequence annotations follow Figure 1. In dark blue are depicted the five canonical mt protein coding genes (*cox1*, *cox2*, *cob*, *nad1* and *nad5*), in red the two rRNA subunits (*rns* and *rnl*), in green unknown ORF. All other colored arrows indicate repeated elements. Repeated elements the same sequence have the same color. Repeats shorter than 100 base pairs were not annotated. Regions with lower or higher mapping coverage were challenging for assembly (see Methods).
