# Supplementary material for: Evolution of myxozoan mitochondrial genomes: insights from myxobolids
Source: BMC Genomics. 2024 Apr 22;25:388. doi: 10.1186/s12864-024-10254-w (PMC11034133; doi:10.1186/s12864-024-10254-w)
Supplement: Supplementary file 4 — Supplementary Material 4 [file 12864_2024_10254_MOESM4_ESM.docx]

**
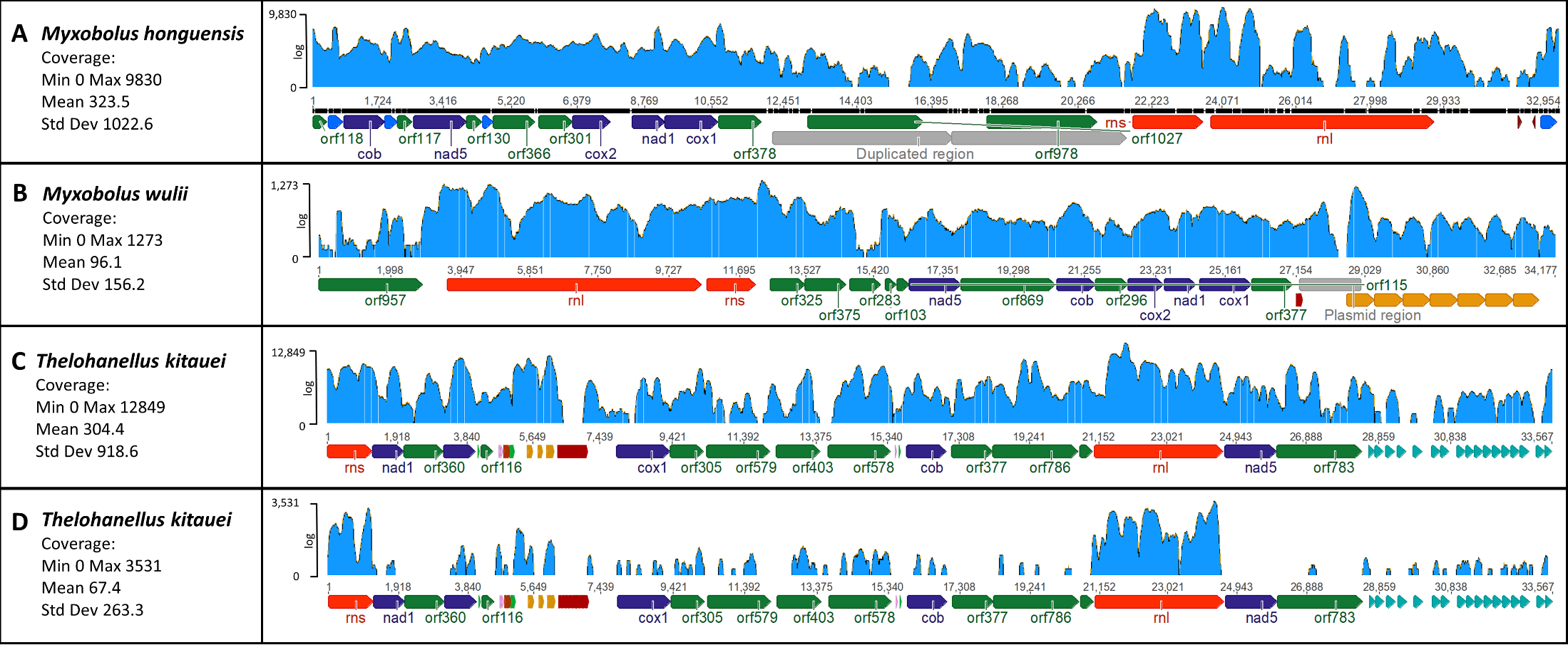
**

**Additional file 4 – RNA coverage depth of the mt chromosomes.**

Illumina reads available in public databases were mapped to each chromosome individually using Geneious Prime version 2023.2.1 (see Methods). A) *M. honghuensis* (SRR16897929); B) *M. wulii* (SRR17138786); C) *T. kitauei* (SRR17152144 & SRR17152863); D) *T. kitauei* (SRR1103279). Each species sequence is shown schematically below the per-base coverage, indicated in light blue. The sequence annotations follow Figure 1. In dark blue are depicted the five canonical mt protein coding genes (*cox1*, *cox2*, *cob*, *nad1* and *nad5*), in red the two rRNA subunits (*rns* and *rnl*), in green unknown ORFs. All other colored arrows indicate repeated elements. Repeated elements the same sequence have the same color. Repeats shorter than 100 base pairs were not annotated.
