# Supplementary material for: Evolution of myxozoan mitochondrial genomes: insights from myxobolids
Source: BMC Genomics. 2024 Apr 22;25:388. doi: 10.1186/s12864-024-10254-w (PMC11034133; doi:10.1186/s12864-024-10254-w)
Supplement: Supplementary file 5 — Supplementary Material 5 [file 12864_2024_10254_MOESM5_ESM.docx]

**
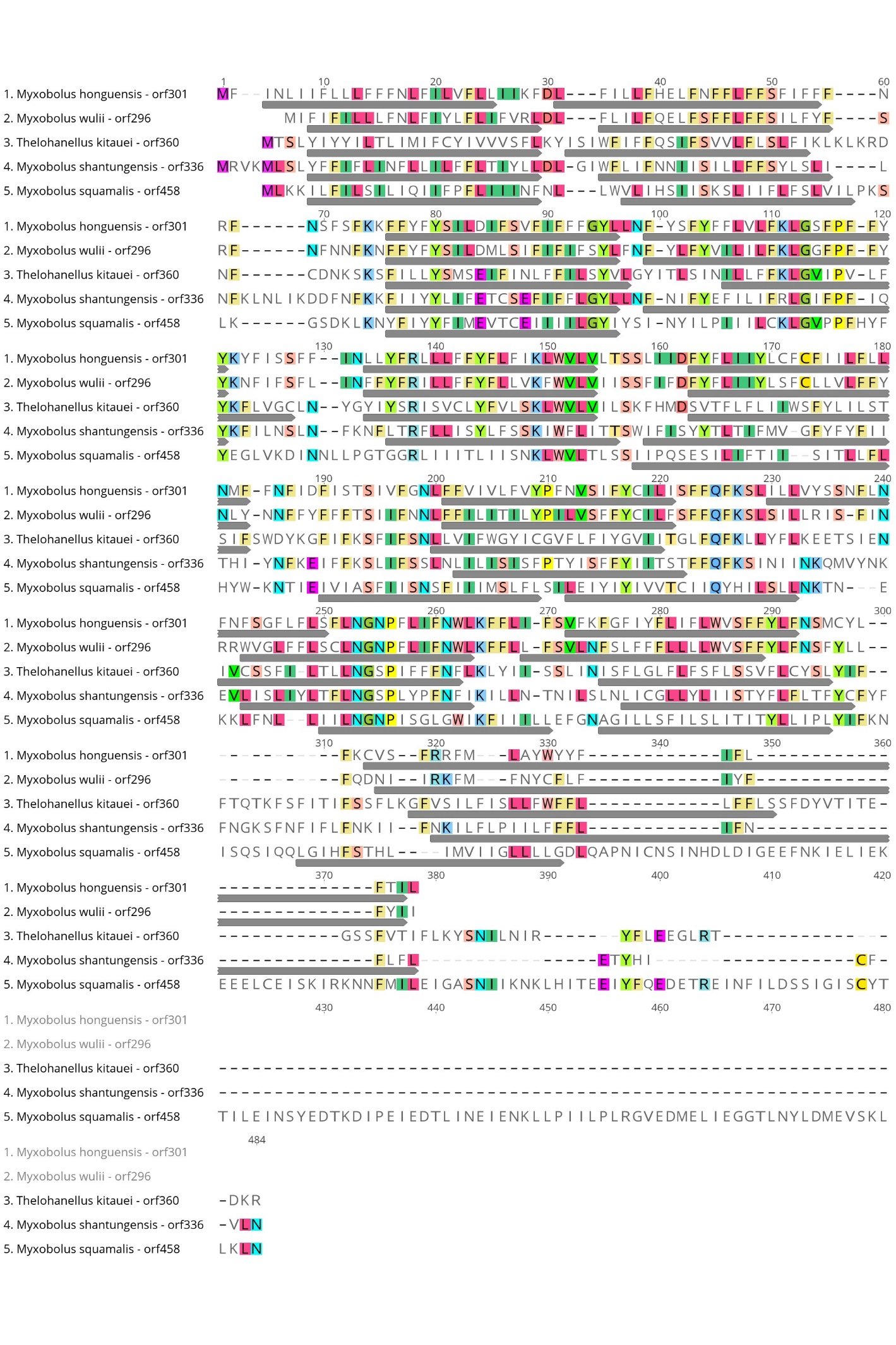
**

**Additional file 5A.**

**
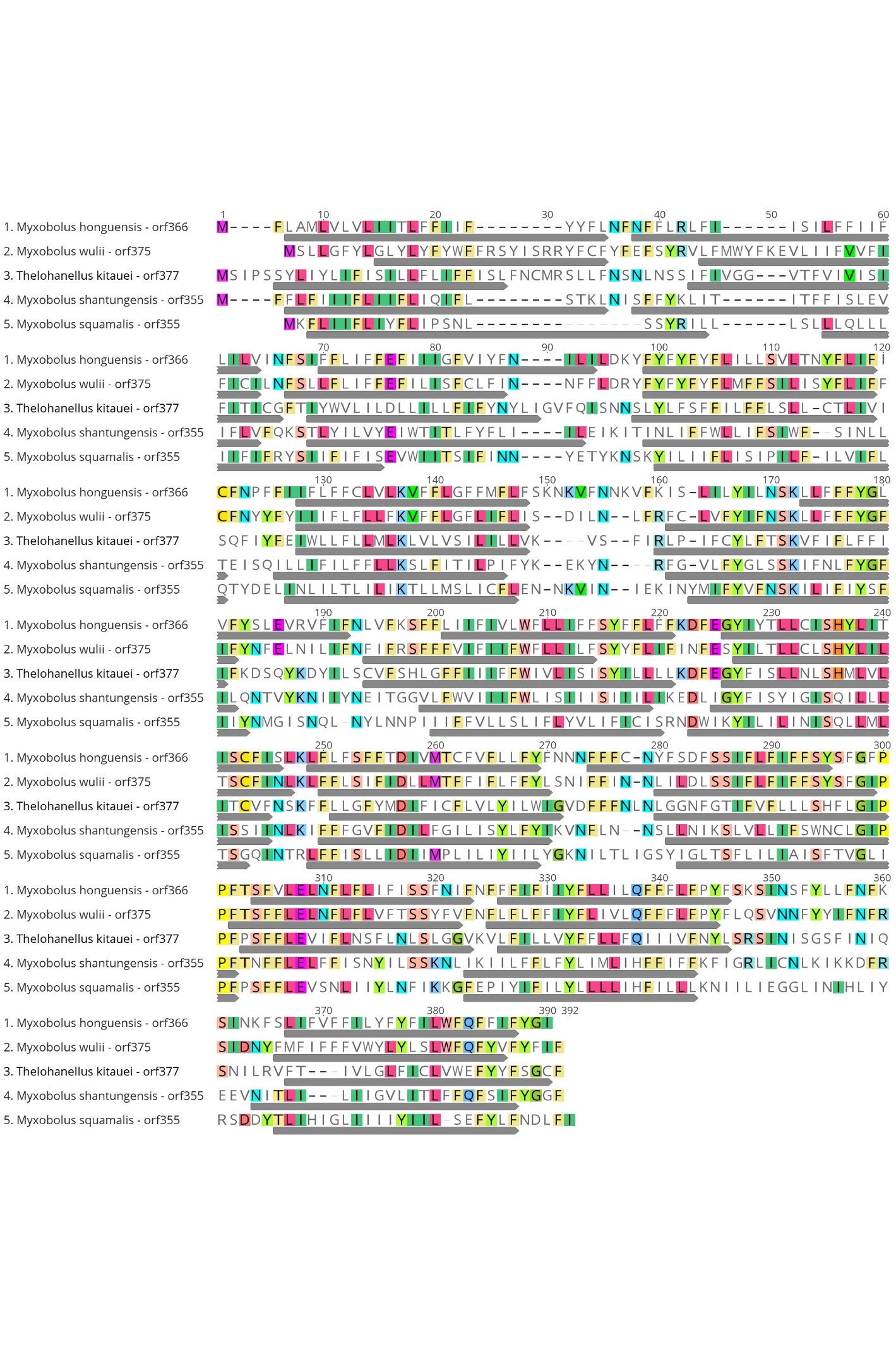
**

**Additional file 5B.**

**Additional file 5 – Alignments showing sequence similarity between unknown ORFs from myxobolid species.**

Amino acids positions shared by two sequences or more are colored. The gray arrows indicated putative transmembrane domains. A) Alignment of *M. honghuensis* orf301, *M. wulii* orf296, *T. kitauei* orf336, *M. shantungensis* orf 336 and *M. squamalis* orf458. B) Alignment of *M. honghuensis* orf366, *M. wulii* orf375, *T. kitauei* orf377, *M. shantungensis* orf 355 and *M. squamalis* orf355.
