# Supplementary material for: Evolution of myxozoan mitochondrial genomes: insights from myxobolids
Source: BMC Genomics. 2024 Apr 22;25:388. doi: 10.1186/s12864-024-10254-w (PMC11034133; doi:10.1186/s12864-024-10254-w)
Supplement: Supplementary file 6 — Supplementary Material 6 [file 12864_2024_10254_MOESM6_ESM.docx]

#NEXUS

[Additional file 6 – 18S rRNA sequence alignment used for phylogenetic reconstructions. Nucleotide sequence alignment, in Nexus format. BMC does not allow text format]

begin data;

dimensions ntax=14 nchar=2201;

format datatype=dna missing=? gap=-;

matrix

Thelohanellus_kitauei AATCTGGTTGATCCTGCCAGTCAG-GCAAGCTATTCTCGAAGA-TTAAGCCATGTATGTGCCAAGTTCAAACG---TTTTACGTGAGACTGCGGACGGCTCAGTATATCAGTGATAATCTGTTTGATTGTCTCTC-CCAT-TGGATAACCGTGGGAAATCTAGAGCTAATACGTGCAGTTCATTGGCTCG--------TCTTCGGACGAGTCAAAGCATTTATTAGACTAAACCATCTACTATG---------------CTCGCATAGTAAGGGGAATCTAGATAACTTTGCTGATCGTATGGCCTAGTGCCGGCGACGTTTCAATTGAGTTTCTGCCCTATCAACT--TGTTGGTAAGGTATTGGCTTACCAAGGTTGCAACGGGTAACGGGGAATCAGGGTTCGATTCCGGAGAGGGAGCCTGAGAAACGGCTACCACATCCAAGGAAGGCAACAGGCGCGCAAATTACCCAATCTAGACAGTAGGAGGTGGTGAAGAGAATTACTAGGTGGTGACTCAATGAGTTACCAGTTTGGAATGAACGTAACTTAAGAAATTCGATGAGAAACAACTGGAGGGCAAGTCCTGGTGCCAGCAGCCGCGGTAATTCCAGCTCCAGTAGTTTGCTTTAAAGTTGTTGCGTTTAAAACGCTCGTAGTTGGATCACGCAGCAGTGCCCAGTAATCTAC----------TATTCGACGTA-----TCACTGAAAACCACTTGT--------------GTGGCCTTTCATGAGCT----------GTCATTAGCAGATACCAACGCTGAGCACTGTTAGTTGCACGTGAGATGAATT-GTTGGCCTTTATTGAGCCGGTATTCTCGTCTTGCGGAGTGTGCCTTGAATAAAACAGAGTGCTTAAAGCAGGTCGT-TG-CCTGAATGTT-ATAGCATGGAACGAACAATCGTGTATATGTGTGTATCCTAGATTGGTGACGA-GCCTTAGGCTTGTTGTT---GATCTGGATGCATACGGCACCCACCT--AAATATGGCTGTTGGTT---CCATATACGGTGATGATTAAAAGGAGCGGTTGGGGGCATCGGTATTTGGCCGCGAGAGGTGAAATTCTTAGACCGGCCAAGGACTAACAAATGCAAAGGCACTTGTCTAGACCGTTTCCATTAATCAAGAACGAAAGTGGGAGGTTCGAAGACGATCAGATACCGTCCTAGTTCC-CACCGTAAACTATGCCGACCTGGGATCAGTTTAGAGATG---TTACAAGCTCTAGATTGGTCCCCCTGGGAAACCTCAAGTTTTTCGGTTACGGGGAGAGTATGGTCGCAAGGCTGAAACTTAAAGGAATTGACGGAAGGGCACCACCAGGGGTGGAGCCTGCGGCTTAATTTGACTCAACACGGGGAAACTTACCTGGTCCGGACATCGATAGGATTAACAGATCAATAGCTCTTTTATGATGCGATGAGTGGTGGTGCATGGCCGTTCTTAGTTCGTGGAGTGATCTGTCGGCCTAATCGCGGTAACGAACGAGACCATAATC-TCCATTTAAGAGATAGAAGCAGACGTCTGTGCG---AGTGGAGTCGCAAGATTTCACCCGTCGGGTGTTGCAGGTTGCATTGTGCGTCGCATTGTCAA----AGTGT--AGGGGCAACCTGAAACTT--------------------------TGGTG--GTGTG-GTGTGCTTTGTTCCCTTCTATTGAGCAGCAATCGGT----CTCGACTGGTTGTTGCCTTATGGAGAGACAACGAGGT--ATATACAAGCTCGAGGAAGAGTGGCTATAACAGGTCAGTGATGCCCTTCGATGTTCAGGGCTGCACGCGCGCTACAATGATAACGACAACGAGT---GTCTGCGTCGAAAGACGTGGGTAA-TCTT--TAATCGTTATCGTGATGGGGATTGACCGTTGTAACTGTCGGTCATGAAAGAGGAATCCCTAGTATGCGTATTTTA-TTAGAATGCGCAGATTAAGTCCCTGCCCTTTGTACACACCGCCCGTCGCTACTACCGAGTGAATGGTGTCATGATGCGCTGGGACTGGACGTTGGATGGGTCCTCGGGCT-----CGTCCGACGCTGGGATCAGCGTAAAATGGCGCAATTTCGAGGAAGTAAAAGTCGTAACAAGGTTTCCGTAGGTGAACCTGCGGAAGGATCATTG

Myxobolus_shantungensis AATCTGGTTGATCCTGCCAGTAGG-GCAAGCTATTCTCGAAGA-TTAAGCCATGTATGTGCCAAGTTCATACG---TTAAACGTGAGACTGCGGACGGCTCAGTATATCAGTGATTATCTGTTTGATTGTCTCTC-CCAT-TGGATAACCGTGGGAAATCTAGAGCTAATACATGTAGTTAATTGGCGTG--------CACTTGTGCACGTCAAAGCATTTATTAGATTAAACCATCTACTACGT------------------TGTAGTAAGACGAATCTAGATAACTTTGCTGATCGCATGGCCTCGTGCCGGCGACGTTTCAATTGAGTTTCTGCCCTATTAACT--TGTTGGTAAGGTATTGGCTTACCAAGGTTGCAATGGGTAACGGGGAATCAGGGTTCGATTCCGGAGAAGGAGCCTGAGAAACGGCTACTACATCCAAGGAAGGCAACAGGCGCGCAAATTACCCAATCTAGACAGTAGGAGGTGGTGAAGAGAATTACTAAGTGGTGGTTCAATGAACTACCAACTTGGAATGGACGTAACTTAAGCAATTCGTTGAGAAACAACTGGAGGGCAAGTCCTGGTGCCAGCAGCCGCGGTAATTCCAGCTCCAGTAGTTTGCTTTAAAGTTGTTGCGTTTAAAACGCTCGTAGTTGGATCATGCAACAGTGTACGGTAATCTAT---------ACACTCGGTGGG-----AATATTCTGAGTGTTCATCGAAAG--------ATGGGGCTCGAAGTCCC----------GCCGATGTTAGATATAAAAGCTGGACACTGTTAATTGCACGTGAGATGGATT-GTTGACCTTCGGTGCGTCGGTAATCCTGTCTTGCGGAGTGTGCCTTGAATAAAACAGAGTGCTTAAAGCAGGTTTA-CG-CCTGAATGTT-ATAGCATGGAACGAACAATAGTGTATTAGTGCATGATTTCGGGCAGCGATCG-ATCTATGGTTTGTTGCT---GTCGGGGAGGTGCACATCACCCACCA--AAATATGGCTGTTGGTT---CCATATACGGTGATGATTAAAAGGAGCGGTTGGGGGCATCGGTATTTGGCCGCGAGAGGTGAAATTCTTGGACCGGCCAAGGACTAACAAATGCAAAGGCACCTGTCTAGACCGTATCCATTAATCAAGAACGAAAGTGGGAGGTTCGAAGACGATCAGATACCGTCCTAGTTCC-CACTGTAAACTATGCCGACCTGGGATCAGTTTAGAGATG---CTACAAGCTCTAAATTGGTCCCCCTGGGAAACCTCAAGTTTTTCGGTTACGGGGAGAGTATGGTCGCAAGGCTGAAACTTAAAGGAATTGACGGAAGGGCACCACCAGGGGTGGAGCCTGCGGCTTAATTTGACTCAACACGGGGAAACTTACCTGGTCCGGACATCGATAGGATACACAGACTGATAGATCTTTGTTGATGCGATGAGTGGTGGTGCATGGCCGTTCTTAGTTCGTGGAGTGATCTGTCAGCTTAATTGCGGTAACGAACGAGACCACAGTC-CTCATTTAAGAAACAGAAAAAGACGGCCTACCA---ATGGACTTGTCCG-------TGGGTGGGTTGTTGTTGGTTGGAGGTGCGATGGTATGGTGAC----GTTGCAGAGGAGCGATCCGATGTGG--------------------------CGGAGCTGTGCG-GTTGAGCCTTTTCCTTTCTGTCAAGCAGTAATCGGT----ATCTGCTGGTTGCTGTCTTATGAGGAGACAACGAGGT--ATAAACAAGCTCGAGGAAGAGTGGCTATAACAGGTCAGTGATGCCCTTCGATGTTCAGGGCTGCACGCGCGCTACAATGATAACGACAACGAGT---GTCCGCGTCGAAAGACGTAGGTAA-TCTT--TAATCGTTATCGTGATGGGGATTGACCGTTGTAATTGTCGGTCATGAAAGAGGAATCCCTAGTATGCATATTTTA-TTAGAATGTGCAGATTGAGTCCCTGCCCTTTGTACACACCGCCCGTCGCTACTACCGAGTGAATGGTGTCATGATGCCCTGGGACTGGACGTCTGGCGGGATTAACCTCT-----TGCAAGATGCTAGAATCAGTGTAAAATGGCGCAATTTCGAGGAAGTAAAAGTCGTAACAAGGTTTCCGTAGGTGAACCTGCGGAAGGATCATTG

Myxobolus_honghuensis AACCTGGTTGATCCTGCCAGTGAA-ACATGCTATTCTCGAAGA-CTAAGCCATGCACGTGCCAAGTTCATACGA-TCTTATCGTGAGACTGCGGACGGCTCAGTATATCAGTGATTATCTGTTTGATTGTCTTGC-CCAT-TGGATAACCGTGGGAAATCTAGAGCTAATACATGCAGTTTATTGGCGTAGT------TGAAAGACTATGTCAAAGCATTTATTAGACTTAACCAACTACTATA-------------CGCAAGTATGGTAAGGCGAATCTAGATAACTTTGCTGATCGTATGGCCCTGTGCCGACGACGTTTCAATTGAGTTTCTGCCCTATCAATT--TGTTGGTAAGGTATTGGCTTACCAAGGTTGCAACGGGTAACGGGGAATCAGGGTTCGATTCCGGAGAGGGAGCCTGAGAAACGGCTACCACATCCAAGGAAGGCAGCAGGCGCGCAAATTACCCAATCTAGACAGTAGGAGGTGGTGAAGAGAAGTACTTAGTGGTGGCCTTAATGG-TCCCAACTAGGAATGAACGTAATTTAAGCAATTCGATGAGTAACTACTGGAGGGCAAGTCCTGGTGCCAGCAGCCGCGGTAATTCCAGCTCCAGTGGCGTGATTTAAAGTTGCTGCGTTTAAAACGCTCGTAGTTGGATCATGCAATAACATGTAGTAACACTGGTTGGT--------------------AAATTTGACGATTCTCTTC-------------TTGATTATTGGATAATT----------ATCGACC-AGTGTGTTCATGCTACATGTTATTATTTGCACACAAGTATGATA-TTTGGGCTTAAGTGATTCGAGTATCATGTCTTGTGGAGTGTGCCTTGAATAAAACAGAGTGCTCAAAGCAGGCGAA-CG-CTTGAATGTT-GTAGCATGGAACGAACAAACGTGTATTTGTGTATATTTGA-ACGGTCGGTGGCAACACTGAC-----------TGTTTGGGTATATGCAGCACCCGCCG--AAATGCGAATGTTGGTTT--TCGTATAAGGTGATGATTAAAAGAAGCGGTTGGGGGCATTGGTATTTGGCCGCGAGAGGTGAAATTCTTGGACCGGCCAAGGACTAACAGATGCGAAGGCGTTTGTCTAGACCGTTTTCATTAATCAAGAACGAAAGTGGGAGGTTCGAAGACGATCAGATACCGTCCTAGTTCC-CACTATAAACTATGCCGACCTGGGATCAGTTTAGTGAT----TAACAAGCTCTAGGTTGGTCCCCCTGGGAAACCTCAAGTTTTTCGGTTACGGGGAGAGTATGGTCGCAAGTCTGAAACTTAAAGGAATTGACGGAAGGGCACCACCAGGGGTGGAACCTGCGGCTTAATTTGACTCAACACGGGGAAACTTACCTGGTCCGGACATCGAAAGGATAGACAGACTGATAGATCTTTCTTGATGCGGTGAGTGGTGGTGCATGGCCGTTCTTAGTTCGTGGAGTGATCTGTCAGGTTAATTCCGGTAACGAACGAGACCACAATC-TTCATTTGAGAAATAGTAGTAGGGAGTTGGCTC---AGTGGTGTTTCGGCAGCTC-TGGGTTGGCTTTCGTAGGTAGAATTATTGAATTTCATAAAAG---TAGCATTCTGGGCTCGCTCAGGGTGTA------------------ATGTTTATGAAA--------GGATATGGTTTTCCCTACTGTTATGCAGTGTTAGGC----AAAACCTTTACGCTGCCTCATGGAGAGACAAC-AGGT--TTATAAAAGCCTGAGGAAGTGTGGCTATAACAGGTCAGTGATGCCCTTCGATGTTCAGGGCTGCACGCGCGTTACAATGATAACAGCAGCGAGT---ATCTGGGTCGAAAGACTTGGGTAA-TCTT--TAATTGTTATCGTGATGGGGATTGACGGTTGTAA--ATCCGTCATGAAATAGGAATCCCTAGTATGTGCAATTTA-TTAAATTGCGCAGATTTAGTCCCTGCCCTTTGTACACACCGCCCGTCGCTACTACCGAGTGAATTATGTCATGATGCTCTGGGACTGGACGCTGAACGGGGGTTAAAGCC-----TGTTCGGTGCTAGAATCAGCGTAAAATGGCGCAATTTCGAGGAAGTAAAAGTCGTAACAAGGTTTCCGTAGGTGAACCTGCGGAAGGATCATTA

Myxobolus_wulii AACCTGGTTGATCCTGCCAGTGAA-ACATGCTATTCTCGAAGA-CTAAGCCATGCACGTGCCAAGTTCATACGA-TCTTATCGTGAGACTGCGGACGGCTCAGTATATCAGTGATTATCTGTTTGATTGTCTTAC-CCAT-TGGATAACCGTGGGAAATCTAGAGCTAATACATGCAGTTTATTGGCGTAGT------CGCAAGATTGCGTCAAAGCATTTATTAGACTTAACCATCTACTGTA-------------CGCAAGTATAGTAAGGCGAATCTAGATAACTTTGCTGATCGTATGGCCCTGTGCCGACGACGTTTCAATTGAGTTTCTGCCCTATCAATT--TGTTGGTAAGGTATTGGCTTACCAAGGTTGCAACGGGTAACGGGGAATCAGGGTTCGATTCCGGAGAGGGAGCCTGAGAAACGGCTACCACATCCAAGGAAGGCAGCAGGCGCGCAAATTACCCAATCTAGACAGTAGGAGGTGGTGAAGAGAAGTACTTAGTGGTGGCC-TAATGG-TCCCAACTAGGAATGAACGTAATTTAAGCAATTCGATGAGTAACTACTGGAGGGCAAGTCCTGGTGCCAGCAGCCGCGGTAATTCCAGCTCCAGTGGCGTGATTTAAAGTTGCTGCGTTTAAAACGCTCGTAGTTGGATCACGCAGTAGCATACAGTTACACAGATTGGT--------------------TTATTTGACGATTTTCTTTCA-----------AAGATTATTGAATTTTG----------GCTGGTC-TGTGTGTTAACGCTGTATGCTGCTATTTGCACACAAGTATGGTA-TTTGGCCTTTAGTGAGTCGAGTATCATGTCTTGTGGGGTGTGCCTTGAATAAAACAGAGTGCTCAAAGCAGGCGAA-CG-CTTGAATGTT-ATAGCATGGAACGAACAAACGTGTATTTGCGTATATTTGATAAGGTCGAGGGCAACTTTGAC-----------CTGTTGGATATATGCAGCACCCGCCA--AAATACGGATGTTGGTTT--TCGTATAAGGTGATGATTAACAGGAGCGGTTGGGGGCATTGGTATTTGGCCGCGAGAGGTGAAATTCTTGGACCGGCCAAGGACTAACAGATGCGAAGGCGTTTGTCTAGACCGTTTCCATTAATCAAGAACGAAAGTGGGAGGTTCGAAGACGATCAGATACCGTCCTAGTTCC-CACTATAAACTATGCCGACCTGGGATCAGTTTAGTGAT----TAACAAGCACTAGGTTGGTCCCCCTGGGAAACCTAAAGTTTTTCGGTTACGGGGAGAGTATGGTCGCAAGTCTGAAACTTAAAGGAATTGACGGAAGGGCACCACCAGGGGTGGAGCCTGCGGCTTAATTTGACTCAACACGGGGAAACTTACCTGGTCCGGACATCGAAAGGATAGACAGACTGATAGATCTTTCTTGATGCGGTGAGTGGTGGTGCATGGCCGTTCTTAGTTCGTGGAGTGATCTGTCAGGTTTATTCCGGTAACGAACGAGACCACTTTC-TCCATTTAAGAAACGGTAGCAGGGAGTTGGCTT---GAAATTGTTTCGGCAGTTT-CGGGTTGATTTTCGCAGATAGAATTGTTAAATTCCATGGAAG----GGTGCTGAGGGGCAACCTGAGGTAT-------------------TTGACTGTGGAA--------GGAGATGATTTTTCCTATCGTTATGCAGTGTAAGGC----AAAACCTTCACGCTGTCTTATGGAGAGACAAC-AGGT--TTATAAAAGCCTGAGGAAGTGTGGCTATAACAGGTCAGTGATGCCCTTCGATGTTCAGGGCTGCACGCGCGCTACAATGATAACAACAACGAGT---GTCTGGGTCGAAAGACTTGGGTAA-TCTT--TAATTGTTATCGTGATGGGGATTGACGATTGTAA--ATTCGTCATGAAATAGGAATCCCTAGTATGTGTAATTTA-TTAAATTGCACAGATTTAGTCCCTGCCCTTTGTACACACCGCCCGTCGCTACTACCGAGTGAATTATGTCATGATGCTCTGGGACTGGACATTGTACGGGTGTCAAAGCC-----TGTTCGATGCTAGAATCAGCGTAAAATGGCGTTATTTCGAGGAAGTAAAAGTCGTAACAAGGTTTCCGTAGGTGAACCTGCGGAAGGATCATTA

Henneguya_salminicola TATCTGGTTGATCCTGCCAGTAAACATACGCTGTTTTCTAAGACTTAAGCCATGCAGGT-GCTACAGTACATACGTGAATTCGTGAGACTGCGGAAGGCTCAGTAAATCAGTTATCATCTATTTGATTGTCTAGC-CCAT-TGGATAACCGTGGGAAATCTAGAGCTAATACATGTAGTTAATTGGTGGTGTAGTATTTATATTGTACCACCAATGCATTTATTAGATGAAGCCAACTGCTTGTT-GGGGGTGCAAATCTCCAACTTGTAAGGTGAGTCTAGATAACTGTGCAGATCGTATGGCCTTGTGCTGACGACGTTTCAATTGAATTTCTGCCCTATCAACT--AGTTGGTAAGGTAGATGCTAACCAAGGTTGTGACGGGTAACGGGGAATCAGGGTTCGATTCCGGAGAGGGAGCTTGAGAATTGGCTACCACATCCAAGGAAGGCAGCAGGCGCGCAAATTACCCAATTCAGACAGTGAGAGGTGGTGACGAGAATTACCAAGTGGTGACCATAAGGT-TGCCAATTTGGAATGAACGTAATTTAAGAAATTCGATGAGTAACAACTGGAGGGCAAGT-CCGGTGCCAGCAGCCGCGGTAATTCCGGCTCCAGTAGCGTATTTTAAAATTGTTGCGCTTAAAACGCTCGTAGTTGGATGACACAGTCATGATCAGTTGATTGTCATGGTTATACATTAAACTTTGACTCTCTACTGTAGATGTCTAA--------------CGACGTATATGGTGTTGAGTTGAAAGTATAACTATGGCAATTGTGTGCTGAATGTGACTAGTTGCACGTGAGGATGATG-GTTGACCTTTAGTGCGTTGACTGTTATGTCTCACGGAGTGTGCCTTGAATAAATCAGAGTGCTCAAAGCAGGCTTTAAG-CTTGAATGTTAATAGCATGGAACGAACAAACGTGTTTATGTATAATATAATTGATTTTGATTT-GTCTATGATAGATTATTG-TGTTTTTGTGTTGTACTGCACCAACCACCAATAACGGACGTTGGTTC--CGTATTGGGGTGATGATTAAAAAGAGCGGTTGGGGGCATTGGTATTTGTCCGCGAGAGGTGAAATTCTTGGACCGGACAAGGACTAACAAATGCGAAGGCATTTGCCTAGACCGTCTCTATTAATCAAGAACGATAGAGGAAGGATCGAAGAGGATCAGATACCCTCGTAGTTTC-CTCCGTAAACTATGCCAACCCGGGATGAGTTTAGAGCTAA-TTATAACGCTCTAGGTTGGTCCCCCTGGGAAACCTGAAGTTTTTCGGTTGCGGGGGGAGTATGGTTGCAAAGCTGAAACTTAAAGGAATTGACGGAAGGGCACCACCAGGAGTGGAGCCTGCGGCTTAATTTGACTCAACACGGGAAAACTTACTCGGTTCGGACATTGACAGGATAAACAGTTTGATAGAACTTTTTTGATACGGTGAATGGTGGTGCATGGCCGTTTTTAGTTCGTGGAGTAATCTGTCAGGCTAACCCCGGTAACGAACGAGATCAGCGTC-TCCATTTGGGGTGTACAAGAAATCTATCGGTAGTGCTGCATTGTTTCGACAGTGT-CACATTTATTGATAGTTAGAGGCTGTGTGGGTGTGCTGTTGCCGATGGTAATGTCAACTCGTTAAAAGGTTG------------------ATATTGCTTAAGGTGGCTGTTTAGCTTGCACTCCCTTGTACCAAACAGTATACAGT----GTAAACTGTATGCTGTCTCATGGAGAGACGGGTGGAT--ATAATCAAACCACAGGACGGCTGGCAAAAACAGGTCTGTTATGCCCTTAGATATCCGAGGCGGCACGCGCGCTACAATGATAACGACAGCGAGT---TTCTAGGTTGAAAGACCTGGGTAA-TCTT-TGAATCGTTATCGTGATGAGGATTGACGGTTGTAATTTTCCGTCATGAAATAGGAATTCCTCGTATGTGTACGTCA-TTAGCGTGTACAGAATAAGTCCCTGCCCTTTGTACACACCGCCCGTCGCTACTACCGAGTGAATGGTGTTATGATGCCTTGGGACTGGACGTTGTTTTGACTAGTAAAATAGTTGAGAGTGACGCTGGGATCAATGTAAAATAGCGCAATTTCGAGGAAGTAAAAGTCGTAACAAGGTTTCCGTAGGTGAACCTGCGGAAGGATCATTG

Myxobolus_squamalis AATCTGGTTGATCCTGCCAGTAAATATACGCTTTTCTCTAAGA-CTAAGCCATGCACGT-TTAAGTTCATACGTAGTAAAACGTGAGACTGCGGACGGCTCAGTAAATCAGTTATCATCTATTTGATTGTCTAC--CCAT-TGGATAACCGTGGGAAATCTAGAGCTAATACATGCAGTTTTGGGATAGCGT----------AAGTTGTCTCACGGCATTTATTGGACAAAACCAACTACCGACGTAGCAGCTTGCTGTTGCGACGCGTAAGGTGAATCTAGATAACTTTGCTGATCGTATGGCCTAGTGCCGGCGACGTTTCAATTGAATTTCTGCCCTATTAACT--TGTTGGTAGTATAGTTGCCTACCAAGGTTGCGACGGGTGACGGGGAATCAGGGTTCGATTCCGGAGAGGGAGCCTGAGAAACGGCTACCACATCCATGGAAGGCAGCAGGCGCGCAAATTACCCAATCCAGACACTGGGAGGTGGTGACGAGAAGTACTAAGTGGTGGCCCTTAGGGTCGCTAGCTTGGAATGGACGTAATTTAAGTAATTCGATGAGTAACAACTGGAGGGCAAGT-CTGGTGCCAGCAGCCGCGGTAATTCCAGCTCCAGTAGCGTATTTTAAAGTTGCTGCGTTTAAAACGCTCGTAGTTGGATCACGCAGTGTAAGTTGGTAAGCTGATTGAATGGTGCTCCAACTGTT-----TTGGTAATAAATTTCTAT--------------TTATTACTAAAACAGTG----------TGCCTTCTTTCAGTTATTCGCCAATTTACACTACTTACGCGTAAGGATGGCA-GTTGACCTTTAGTGCGTCGATTACCGTGTCTTACGGAGTGTGCCTTGAATAAATCAGAGTGCTCAAAGCAGGCTTT-TG-CTTGAATGTTAATAGCATGGAACGAACAATTGTGTAGTAGTATGTTGTGACGCATAGCGATCG-GTCTTTGACTGAATGCTATTGCTGTTGCAGCATACAGCACCAACCACCAATAACGGATGTTGGTTC--CGTATTGGGGTGATGATTAAAAGGAGCGGTTGGGGGCATTGGTATTTGGCCGCGAGAGGTGAAATTCTTAGACCGGCCAAGGACTAACGAATGCGAAGGCATTTGTCTAGACCGCCTCGCTTAATCAAGAACGATAGTGGGAGGTTCGAAGACGATCAGATACCGTCCTAGTTCC-CACTGTAAACTATGCCGACCCGGGATCAGCATGAAGCTC--TATATATGCTTGATGTTGGTCCCCCTGGGAAACCTCAAGTTTTTCGGTTACGGGGAGAGTATGGTCGCAAGGCTGAAACTTAAAGGAATTGACGGAAGGGCACCACCAGGAGTGGAGCCTGCGGCTTAATTTGACTCAACACGGGAAAACTTACCAGGTCCGGACATCAATAGGATAGACAGACTGATAGATCTTTCTTGATATGATGGATAGTGGTGCATGGCCGTTCTTAGTTCGTGGAGTGATCTGTCAGGCTAATCCCGGTAACGAACGAGATCTTATTC-TCCATTTGATGAGCGGAAGCAGATGGTGGCTTG---AAAATTGTCTCGATGAAAT-TCAAGTTACCATCGAAGGCAGTGTTTGCGAGTTTATTGTTGA----AATATAAAGAGTTGCGAGAACGGTCTTAACCCCATTCTGGTAGCAATTTGTACTAAATGTAAA-TTTGTTGGCATTCCCTTCCGTTATACGCTGTTCTACTTACCAAAGTGGAGCAGTGTGTCATGGAGAGACTGTGAGGTATATATCCAAGCTCAATGAAGCTAGGCCATAACAGGTCTGTGATGCCCTAAGATGTCCTGGGCTGCACGCGCGCTACAATGATGGTGACAGCAAGT---TTCTAGGTCGAGAGACCCGGGCAA-TCTT-GTAATCGCCATCGTGATGGGGATTGACCATTGTAAT--TTGGTCATGAAATAGGAATTCCTTGTAGGCACACTTTA-TTAGAGTGTGCCGAACGAGTCCCTGCCCTTTGTACACACCGCCCGTCACTACTACCGAGTGAATCGTGTCATGATGCCTTGGGACCGGACGTATTTGGGGCTGCAAGGCT-----CAAAATATGCTGGGATCGATGTAAAATGGTGCAATTTCGAGGAAGTAAAAGTCGTAACAAGGTTTCCGTAGGTGAACCTGCGGAAGGATCATTG

Sphaeromyxa_zaharoni AATCTGGTTGATCCTGCCAGTAGGCATATGCTCGTTTCAAAGA-TTAAGCCATGCATGT-CGAAGTTCATACCT-AGTAAAAGTGAGACTGCGAAAGGCTCAGTATATCAGTTATAGTTTATTCGATTGTGAAAT-CCACATGGATAACCGTGGAAAATCTAGAGCTAATACATGCGAAAAATGATATATTA------AGCAATTAGTATATTATGCATTTATTAGATTAAACCAATCGGAGCT-------------TTTAGTTCCGTTGTGGTGAGTCTGAATAACTTTGCCGATCGTATGGCCTAGAGCCGGCGACGTTTCGATTAAATTTCTGCCCTATCAATT--TGTTGGTAAGGTAGTGGCTTACCAAGATTATTACGGGTAACGGGGAATCAGGGTTCGATTCCGGAGAGGGAGCCTGAGAAACGGCTACCACATCCAAGGAAGGCAGCAGGCGCGCAAATTATCCAATCCAAATTGTGGGAGATGGTAACGAGAAATACCAGGTCGTTTTCAAATGAA-TTCGACACTGGAATGAATGTAACTTAAAAAATTCAATGAGTAGCAACTGGAGGGCAAGT-CTGGTGCCAGCAGCCGCGGTAATTCCAGCTCCAGTAGTGTATCTTAAAGTTGCTGCGGTTAAAACGCTCGTAGTTGGACAATAGAGCAACTTGGTGCAGGCGGGAAAGGTTGTACTTATTTATTAGG---CAGTTAATATGAGCTTATGCGAGTATTGCTACTAATTTATATGTTACTTTGTCGACCTTGTAGCTA---------------AGTTGCTTGGACTATTTTGTGAGAATAATA-TATGGTCTTAATTGTATCGTATATTGTATCTTGCATAGCGTGCCTTGAATAAATCAGAGTGCTCAAAGCAAGCTAA-CG-CTTGGATGTTAATAGCATGGAACGAATGT-TTTGTGAAAGTATGTGC------------ATTT-GGTTTCGGCCAGGTGT-------------ATATACAGCACCAACCA-CGAAGCAGAACATTGGTAGC-TGTCTTCGGGTGATGATTAAGAGGGGCATTTGGGGGCATTAGTATTTGAACGCGAGAGGTGAAATTCTTGGACCGTTCAAGGACTAACAAGTGCGAAAGCATTTGCCAAGGATGTTCCCTTTAATCAAGAACGAAAGTGAGAGGATCGAAGACGATCAGATACCGTCCTAGTTCT-CACCGTAAACTATGCCAACCCGGGATCAGCTCGGATGTACTTTTTATGGATCCAGGTTGGTCCCCCTGGGAAACCTTGAGTTTTTAGGTTCCGGGGGGAGTATGGTTGCAAGGCTGAAACTTAAAGGAATTGACGGAAGGGCACCACCAGAGGTGGAGCCTGCGGCTTAATTTGACTCAACACGGGAAAACTCACCTGGTCCGGACATCGATAGGATTGACAGACTAATAGATCTTTCATGATACGGTGGTTGGTGGTGCATGGCCGTTCTTAGTTCGTGGAGTGATCTGTCAGGTTGATTCCGGTAACGAACGAGACCACGATC-TCCATTTGGGTAGTCGTAAAACCGATTTGAGTGGTTTTGTGCGCTTCGGCAAACT-TAATTAGGCTTATTGAGCTTTACGATGTATGTG-----------------------------------------------------------------------------------------------------------------------CAGCAATGTGCATACCTATGGAGAGACAGCCGGGT-----TTTAAGACGGAGGAAGCGTGGCAATAACAGGTCTGTGATGCCCTTCGATGTTCAGGGCTGCACGCGCGCTACAATGATAACGACAGCGAGT---TTCTGGACTGAAAAGTTCTGGGAAATCTTTTAAATCGTTATCGTGATGGGGATTGAGCATTGTAATTATTGCTCATGAAATAGGAATCTCTTGTAATTGCAACTCACTTAGGTTGTAATGAATTAGTCCCTGCCCTTTGTACACACCGCCCGTCGCTACTACCGAGTGAATGGTGTCATGATGTATTAGGACTGGACGTGTTGGCATTTTCT----------GCGAATACGCTAGGAGTAAGACAAAATGGCCCTATTTCGAGGAAGTAAAAGTCGTAACAAGGTTTCTGTAGGTGAACCTGCAGAAGGATCATTA

LC066366_Kudoa_Iwatai_Japan ---------------------GGTCATATGCTCGTCTCAAAGA-TTAAGCCATGCAAGT-CTAAGTTCACATCA-TATAAAGATGAAACTGCGAAGCGCTCAGTAAATCAGTTATTGTCCGTTCGGTCATATCAG-CCA--TGGATAACTGTGGTAAATCTAGAGCTAATACATAGCAAATCCC-------------------TGTAATAGGGGAGCATTTATTAGACTCAACCAACTGGCTTC-------------------GGCCATTTGATGAATCATAATAACTGAGCATATCG---AACATTAAGTTGTCGATAGTCCGATCGAATTTCTGCCCTATCAACT--AGTTGGTGAGGTAGTGGCTCACCAAGGTTGTGACGGGTAACGGGGGATCAGGGTTCGATTCCGGAGAGGGAGCCTGAGAAACGGCTACCACATCTAAGGAAGGCAGCAGGCGCGCAAATTACCCAATCCAGACTTTGGGAGGTAGTGACGAGAAATACCGGAGTAGACCATTAATTGGTTCACTATCGGAATGAACGTAATCTAATACCTTCGATGAGTAGCTACTGGAGGGCAAGT-CTGGTGCCAGCAGCCGCGGTAATTCCAGCTCCAGTAGTGTATATCAAAATTGTTGCGGTTAAAACGCTCGTAGTTGGATTACAAAAGCTCTATGATGGCCAAGT------------------------------CTAGGTTTGGTTGTTG----------------------------------------------------------------TGGGGTTTTTTTATCGCGAGAGCCGTA-TGTGGGATTAAAT-TCTCGTGTGCGGTCACTTGCGAAATGTGCCTTGAATAAAGCACAGTGCTCAAAGCAGGTGTA-AG-CTTGAATGTA-ATAGCATGGAACGAATAT-ATTGA----------------------------------------------------------------------------CCTTGTCAA-GGTTGGTTG--TTGGCAGTGGTCTCGATTAAAAGGGACATTTGAGGGCGTTAGTACTTGGTGGCGAGGGGTGAAATCCTTAGACCCATCAAAGACTAACTAATGCGAAAGCATTCGCCAAGAGTGTTTTCATTAATCAAGAACGAAAGTTGGAGGTTCGAAGACGATCAGATACCGTCCTAGTTCCATACAGTAAACTATGCCAACATGGGATTAGCCCGGT------------TAATCCAGGTTGGACCCTCGGTGAAAATT--AGTGTTTCGGTTCCGGGGAGAGTGCTCACGCAAGTGCGAAATTTAAAGAAATTGACGGAATGGCACCACCAGGAGTGGAGCCTGCGGCTTAATTTGACTCAACACGGGGAAACTCACCAGGTCCAGACATTGACAGGATTGACAGACTGAGAGATCTTTCATGATACAATGATTGGTGGTGCATGGCCGTTCTTAGTTGGTGGAGTGATCTGTCAGGTTTATTCCGGTAACGAGCGAGACCACGATC-TTTAATTGATTA------------------------------------------------------------------------------------------------------------------------------------------------------------------CGGTTA----------------------------TAATGTATTAGCCGATCTTAAAGAGACAACCGGA------GTTAAGCCGGGGGAAGCGTGGCAATAACAGGTCTGTGATGCCCTTCGATGTTCTGGGCTGCACGTGCGCTACAATGGCAGTGACAATAAGT---ACCTACTCCGAAAGGGGCGGGGAA-TCTTAAAAATCGCTRTCTTGCTGTGGATTGAGCCTTGTAATAATTGCTCATGAAAGAGGAATTCCTCGTAAGCGCGAGTCA-TCAGCTCGTGTTGAATACGTCTCTGCCCTTTGTACACACCGCCCGTCGCTACTACTGACTGGATGCAATTTTGAAGAGCTAGGACTTGCGGAGCCTT------------------GTAGCTCTGTGGGGATAAGCTTTGAAATAATGCATCTGAAAGAAGTAAAAGTCGTAACAAGGTTTCCGTAGGTGAACCTGCGGAAGGATCATTA

Kudoa_iwatai_Israel TATCTGGTTGATTCTGCCAGTGGTCATATGCTCGTCTCAAAGA-TTAAGCCATGCAAGT-CTAAGTTCACATCA-TATAAAGATGAAACTGCGAAGCGCTCAGTAAATCAGTTATTGTCCGTTCGGTCATATCAG-CCA--TGGATAACTGTGGTAAATCTAGAGCTAATACATAGCAAATCCC-------------------TGTAATAGGGGAGCATTTATTAGACTCAACCAACTGGCTTC-------------------GGCCATTTGATGAATCATAATAACTGAGCATATCG---AACATTAAGTTGTCGATAGTCCGATCGAATTTCTGCCCTATCAACT--AGTTGGTGAGGTAGTGGCTCACCAAGGTTGTGACGGGTAACGGGGGATCAGGGTTCGATTCCGGAGAGGGAGCCTGAGAAACGGCTACCACATCTAAGGAAGGCAGCAGGCGCGCAAATTACCCAATCCAGACTTTGGGAGGTAGTGACGAGAAATACCGGAGTAGACCATTAATTGGTTCACTATCGGAATGAACGTAATCTAATACCTTCGATGAGTAGCTACTGGAGGGCAAGT-CTGGTGCCAGCAGCCGCGGTAATTCCAGCTCCAGTAGTGTATATCAAAATTGTTGCGGTTAAAACGCTCGTAGTTGGATTACAAAAGCTCTATGATGGCCAAGT------------------------------CTAGGTTTGGTTGTTG----------------------------------------------------------------TGGGGTTTTTTTATCGCGAGAGCCGTA-TGTGGGATTAAAT-TCTCGTGTGCGGTCACTTGCGAAATGTGCCTTGAATAAAGCACAGTGCTCAAAGCAGGTGTA-AG-CTTGAATGTA-ATAGCATGGAACGAATAT-ATTGA----------------------------------------------------------------------------CCTTGTCAA-GGTTGGTTG--TTGACAGTGGTCTCGATTAAAAGGGACATTTGAGGGCGTTAGTACTTGGTGGCGAGGGGTGAAATCCTTAGACCCATCAAAGACTAACTAATGCGAAAGCATTCGCCAAGAGTGTTTTCATTAATCAAGAACGAAAGTTGGAGGTTCGAAGACGATCAGATACCGTCCTAGTTCCATACAGTAAACTATGCCAACATGGGATTAGCCCGGT------------TAATCCAGGTTGGACCCTCGGTGAAAATC--AGTGTTTCGGTTCCGGGGAGAGTGCTCACGCAAGTGCGAAATTTAAAGAAATTGACGGAATGGCACCACCAGGAGTGGAGCCTGCGGCTTAATTTGACTCAACACGGGGAAACTCACCAGGTCCAGACATTGACAGGATTGACAGACTGAGAGATCTTTCATGATACAATGATTGGTGGTGCATGGCCGTTCTTAGTTGGTGGAGTGATCTGTCAGGTTTATTCCGGTAACGAGCGAGACCACGATC-TTTAATTGATTA------------------------------------------------------------------------------------------------------------------------------------------------------------------CGGTTA----------------------------TAATGTATTAGCCGATCTTAAAGAGACAACCGGA------GTTAAGCCGGGGGAAGCGTGGCAATAACAGGTCTGTGATGCCCTTCGATGTTCTGGGCTGCACGTGCGCTACAATGGCAGTGACAATAAGT---ACCTACTCCGAAAGGGGCGGGAAA-TCTTAAAAATCGCTGTCTTGCTGTGGATTGAGCCTTGTAATAATTGCTCATGAAAGAGGAATTCCTCGTAAGCGCGAGTCA-TCAGCTCGTGTTGAATACGTCTCTGCCCTTTGTACACACCGCCCGTCGCTACTACTGACTGGATGCAATTTTGAAGAGCTAGGACTTGCGGAGCCTT------------------GTAGCTCTGCGGGGATAAGCTTTGAAATAATGCATCTGAAAGAAGTAAAAGTCGTAACAAGGTTTCCGTAGGTGAACCTGCGGAAGGATCATTA

Kudoa_septapunctata GATCG----------------GGTCATATGCTCGTCTCAAAGA-TTAAGCCATGCAAGT-CTAAGTTCACATCA-TTTAAAGATGAAACTGCGAAGCGCTCAGTAAATCAGTTATTGTCCGTTCGGTCATATCAG-CCA--TGGATAACTGTGGTAAATCTAGAGCTAATACATAGCAAATCTCACCATG-------------TAAATGGTGGGAGCATTTATTAGACTCGACCAACTGGCCTC-------------------GGCCATTTGATGAATCCTAATAACTGAGCATATCG---AACATTAATTTGTCGATAGTCCGATCGAATTTCTGCCCTATCAACT--AGTTGGTGAGGTAGTGGCTCACCAAGGTTGTGACGGGTAACGGGGGATCAGGGTTCGATTCCGGAGAGGGAGCCTGAGAAACGGCTACCACATCTAAGGAAGGCAGCAGGCGCGCAAATTACCCAATCCAGACTTTGGGAGGTAGTGACGAGAAATACCGGAGTGGACCGTAAAATGGTTCACTATCGGAATGAACGTAATTTAATACCTTCGATGAGTAGCTACTGGAGGGCAAGT-CTGGTGCCAGCAGCCGCGGTAATTCCAGCTCCAGTAGTGTATATCAAAATTGTTGCGGTTAAAACGCTCGTAGTTGAATTACAAAAGCTCTTTGGCGGCCAAAT------------------------------CTAGGTTTGGTCGTTG----------------------------------------------------------------TGGGGTTTTTTTATCGCGAGAGCCATA-CGTGGGATTAAAT-TCTTGTGTGTGGTCACTTGCGAGGTGTGCCTTGAATAAAGCACAGTGCTCAAAGCAGGCGAA-CG-CTTGAATGTT-ATAGCATGGAACGATTAT-GTTGA----------------------------------------------------------------------------TCTTGTCGACTGTTGGTTG--TTGACAGTGGTCTCGATTAAAAGGGACATTTGAGGGCGTTAGTACTTGGTGGCGAGGGGTGAAATCCTTTGACCCATCAAAGACTAACTAATGCGAAAGCATTCGCCAAGAGTGTTTTCATTAATCAAGAACGAAAGTTGGAGGTTCGAAGACGATCAGATACCGTCCTAGTTCCATACAGTAAACTATGCCAACATGGGATTAGCCCGGTT-----------TAATCCAGGTTGGGCCCTCAGTGAAAACG-TAGTGTTTCGGCTTTGGGGAGAGTGCTCACGCAAGTGACAAATTTAAAGAAATTGACGGAATGGCACCACCAGGAGTGGAGCCTGCGGCTTAATTTGATTCAACACGGGGAAACTCACCAGGTCCAGACATTGGTAGGATTGACAGACTGAGAGATCTTTCATGATTTGATGATTGGTGGTGCATGGCCGTTCTTAGTTGGTGGAGTGATCTGTCAGGTTTATTCCGGTAACGAGCGAGACCACGATC-TTTAATTGATTA------------------------------------------------------------------------------------------------------------------------------------------------------------------CGGTTA----------------------------AAATGTCTTGACCGATCTTAAAGAGACCACCGGA------TTGAAGCCGGGGGAAGCGTGGCAATAACAGGTCTGTGATGCCCTTCGATGTTCTGGGCTGCACGTGTGCTACAATGATAGTGACAACGAGT---ACCTGCTCTGAGAGGGGTGGGAAA-TCTTAAAAATCGCTATCTTGCTTTGGACTGAGCCTTGTAATAATTGCTCACGAAAGAGGAATTCCTCGTAAGCGCGAGTCA-TCAGCTCGTGTTGAATAAGTCTCTGCCCTTTGTACACACCGCCCGTCGCTACTACCGACTGGATGTTGCTTCGAAATGTTAGGACTTGCGGCGTC--------------------TTGACGCTGCGGGGAATAGTTTTTAGATGTAATTTCTGGAGGAAGTAAAAGTCGTAACAAGGTTTCCGTAGGTGAACCTGCGGAAGGATCATCA

Kudoa_hexapunctata TATCTGGTTGATTCTGCCAGTGGTCATATGCTCGTCTCAAAGA-TTAAGCCATGCAAGT-CTAAGTTCATATCA-TCTAAAGATGAAACTGCGAAGCGCTCAGTAAATCAGTTATTGTCCGTTCGGTCTTATCAG-CCA--TGGATAACTGTGGTAAATCTAGAGCTAATACATAGCAAATCTCGT-----------------ACTTGTGCGGGAGCATTTATTAGACTCAACCAACTGGCATC------------------TCGCCATTTGACGAATCCTAATAACTGAGCATATCG---AACATTAATTTGTCGATAGTCCGATCGAATTTCTGCCCTATCAACT--AGTTGGTGAGGTAGTGGCTCACCAAGGTTGTGACGGGTAACGGGGGATCAGGGTTCGATTCCGGAGAGGGAGCCTGAGAAACGGCTACCACATCTAAGGAAGGCAGCAGGCGCGCAAATTACCCAATCCAGACTTTGGGAGGTAGTGACGAGAAATACCGGAGTAGACCGTTAATTGGTTCACTATCGGAATGAACGTAATTTAATACCTTCGATGAGTAGCTACTGGAGGGCAAGT-CTGGTGCCAGCAGCCGCGGTAATTCCAGCTCCAGTAGTGTATATCAAAATTGTTGCGGTTAAAACGCTCGTAGTTGGATTACAAAAGCTCTTTGGCGGTTAAAT------------------------------CAAGGTTTGATCGCTG----------------------------------------------------------------TGGGGTTTTTTTATCGCGAGAGCCGCA-CGTGGGATTAAAT-TCTTGTGTGTGGTCACTTGCGAGGTGTGCCTTGAATAAAGCACAGTGCTCAAAGCAGGCGTA-AG-CTTGAATGTT-ATAGCATGGAACGATTAT-GTTGA----------------------------------------------------------------------------TCTTGTCGACTGTTGGTTG--TTGGCAGTGGTCTCGATTAAAAGGGACATTTGAGGGCGTTAGTACTTGGTGGCGAGGGGTGAAATCCTTAGACCCATCAAAGACTAACTAATGCGAAAGCATTCGCCAAGAGTGTTTTCATTAATCAAGAACGAAAGTTGGAGGTTCGAAGACGATCAGATACCGTCCTAGTTCCATACAGTAAACTATGCCAACATGGGATTAGCCCGGTT-----------TAATCCAGGTTGGGCCCTCAGTGAAAATA--AGTGTTTCGGTTCTGGGGAGAGTGCGCGCGCAAGTGCTAAATTTAAAGAAATTGACGGAATGGCACCACCAGGAGTGGAGCCTGCGGCTTAATTTGATTCAACACGGGGAAACTCACCAGGTCCAGACATTGGTAGGATTGACAGACTGAGAGATCTTTCATGATTTGATGATTGGTGGTGCATGGCCGTTCTTAGTTGGTGGAGTGATCTGTCAGGTTTATTCCGGTAACGAGCGAGACCACGATC-TTTAATTGATTA------------------------------------------------------------------------------------------------------------------------------------------------------------------CGGTTG----------------------------AATTGTCTTGACCGATCTTAAAGAGACCACCGGA------GTGAAGCCGGGGGAAGCGTGGCAATAACAGGTCTGTGATGCCCTTCGATGTTCTGGGCTGCACGTGTGCTACAATGATAGTGACAACAAGC---TCCTGCCCTGAGAGGGGTGGGTAA-TCTTGAAAATCGCTGTCTTGCTTTGGACTGAGCCTTGTAATAATTGCTCACGAAAGAGGAATTCCTCGTAAGCGCGAGTCA-TCAGCTCGTGTTGAATAAGTCTCTGCCCTTTGTACACACCGCCCGTCGCTACTACCGACTGGATGTTGCTTCGAAAAGTTTGGACCTGCGGCGTCAT------------------TTGGCGCTGCGGGGAAAAGCGTTGAGATGTTTCATCTGGAGGAAGTAAAAGTCGTAACAAGGTTTCCGTAGGTGAACCTGCGGAAGGATCATCA

Enteromyxum_leei AATCTGGTTGACTCTGCCAGTGGTCATATGCTCGTCTCAAAGA-CTAAGCCATGCATGT-CCAAGTTCACATCA---ATTTGATGAAACTGCGAAGCGCTCAGTAAATCAGTTATAGTCTATTCGATGGTATAC--ATAG-TGGATAACTGTGGTAAATCTAGAGCTAATACGTGCTAAAT--------------------------------ATGCATTTACTAGCTAGAA-------------------------------ACCAACAAGTTGAATCTAGGTAA-ATCGCGAATCGTATGGCGC-AAGCCGACGATAATTCGATCGAGTTTCTGCTCTATTAACT--AGTTGGTGAGTTCTTTGCTCACCAAGGTTTTAATGGATAACGGGGAATTAGGGTTCGATTCCGGAGAGGGAGCCTGAGAAACGGCTACCACATCTAAGGAAGGCAGCAGGCGCGCAAATTACCCAATCCAGACATTGGGAGGTAGTGACGAGAAATACCGGAGT-TAACCTTCTAGG-TAAACTATCGGAATGAACATAATTTAATACCTTTGTTGAGTATCTATTGGAGGGCAAGT-CTGGTGCCAGCAGCCGCGGTAATTCCAGCTCCAATAGTGTATATCAATATTGCTGCAGTTAAAAAGCTCGTAGTTGAATTACAAAGGATATTGTCAGAGTATGTTTTATA--------------------TCAATAAAATATTCTCATTC----------------------------------------------------------------AATTTCCTTTTTATCGTAAGAGCTAGT-AATGAAATTCACT-TTTTGTTGCTAGTGACTTGCGGAGTGTGCCTTGAATAAAGCAAAGTGCTCAATACAAGCGTC-CG-CTCGAATGTA-GTAGCATGGTACGAATATCGGTGA----------------------------------------------------------------------------CGCCAATCCGTGTTGGTTT--TGGATTGGTGTCTAGATTAAAAGGGACATTTGAGGGCGTTAGTACTTGGTGGCGAGAGGTGAAATTCTTAGACCCACCAAAGACTCACTAATGCGAAAGCGTTCGCCAAGACTGTTTTCATTAATCAAGAACGAAGGTTGGAGGTTCGAAGACGATCAGATACCGTCGTAGTTCCACACAGTAAACAATGCCAACTTGAATTCAGCGTGTATA---------ATCATATACGTTGGCTTCTCCGGGAAACC--AAGTCTTCGGGCTCTGGGGATAGTATAGTTGCAAGTCTGAAATTTAAAGAGATTGACGGAAGGGCACCACCAGGAGTGGAGCCTGCGGCTTAATTTGACTCAACACGGGGAAACTCACCAGGTCCGGACATCGATAGGATTGACAGAATGACAGATCTTTCATGATACGGTGATTGGTGGTGCATGGCCGTTCTTAGTTGGTGGAGTGATCTGTCAGGTTAATTCCGGTAACGAGCGAGACCACAATC-TCTAATTGATTA------------------------------------------------------------------------------------------------------------------------------------------------------------------CATTCCCA-------------------------CTTATTTCGGGATTGATCTTAGAGAGACTACCAAG------TTCAACTTGGGGGAAGTGTGGCAATAACAGGTCTGTGATGCCCTTCGATGTTCTGGGCTGCACGCGCGCTACAATGGCAGCAGCAAATAGT---TGTTTGCTCGAAAGAGTAGACTAA-TCAT--TAATTGCTGTCGTGATCGGGATTGAGCCTTGTAATTATTGCTCATGAAAGTGGAATTCCTCGTAAGCGCAAGTCA-TCAACTTGTGTTGAATACGTCTCTGCCCTTTGTACACACCGCCCGTCGCTACTACCAATTGAATGATTCTGCGAGATCTCAGGAGAGCTAGTACTTG----------------------TACCGGCTTGATCGAGCTCAAGTAGAATCCTTTAAAGGAAGTAAAAGTCGTAACAAGGTTTCCGTAGGTGAACCTGCGGAAGGATCATTA

Polypodium_hydriforme_USA TACCTGGTTGATCCTGCCAGTAGCGATATGCTCGTCTCAAAGT-TTAAGCCATGCATGT-CTCAGTACACGCCC-CTGTACGGTGAAACTGCGAACGGCTCATTACATCAGTTATCGTGTCCTCGATCGTTCCTTACCACATGGATACCCGTGGTAATTCTAGAGCTAATACATGCGCCCAGTC---CCGAC------CGCTCGCCGGAAGGGATGTATTTATTAGATTTTCAGACCATGACGG------------GGCAACCCGTGCGTTGGTGATTCATGGTAACTGCTCGGATCGCACGGCCTCGCGCCGGCGACGCCTCGTTCCAATTTCTGCCCTATCAACTGACGATGGTACGGTAGTGGCCTACCATGGTCGTAACGGGTGACGGAGAATCAGGGTTCGGTTCCGGAGAGGGAGCCCGAGAAATGGCTACCACTTCCACGGAAGGCAGCAGGCGCACACATTGCCCAATCCCGACACGGGGAGGCAGTGACAAGAAATAACGGTGCGTCGGCTTAGGCC-GTCGCAACCGGAATGAGTACGACCCAAATCCTCTAACGAGGATCCATTGGAGGGCAAGT-CTGGTGCCAGCAGCCGCGGTAATTCCAGCTCCAACAGTGTATGCTAATGTTGCTGCAGTTAAAAAGCTCGTAGTTGGATCGCAGGCGGTCGACGTCGAG------------GTGCGCTACACGCG--------------------------------------------------------------------CTACCTCGCGTCGTCGC------------CTTATCCGCGGAGGCCGCGCGGTGCTCTTGATCGAGCGCCGGCCGGTCTACCCGGGACGTCACCTTGAGGAAACTAGAGTGTTCAAGGCAGGTGTCTCGCCCTGAATACC-GCAGCATGGAATGACACGCGGGAC--------------------------------------------------TGTGCGACGCGCAAGCGTCTCGGTC--CGGTGCGCGCGTTGGTCTGACGGACCGCAGTGACGGTTAAGAGGGACGGTCGGGGGCATTCGTATTTCGTCGTGAGAGGTGAAATTCTTAGACCGACGAAAGACGGACAAGTGCGAAAGCGTTTGCCAAGAACGTTTTCATTAATCAAGAACGAAAGTTAGAGGATCGAAGACGATCAGATACCGTCGTAGTTCT-AACCATAAACGATGCCGACCAGGGATCGGCGGGCCGT----GTTCATCGAGCT-CGTCGGCACCTCCCGGGAAACCTGAGTGTTTGGGTTCCGGGGGGAGTATGGTCGCAAGACTGAAACTTAAAGGAATTGACGGAAGGGCACCACCAGGAGTGGAGCCTGCGGCTTAATTTGACTCAACACGGGGAACCTCACCAGGTCCGGACATGACGAGGATTGACAGACTGACCGCTCTTTCTCGATCTCATGGTTGGTGGTGCATGGCCGTTCTTAGTTGGTGGAGCGATTTGTCAGGTTAATTCCGATAACGAACGAGACCTTGTCCGGCTAGTTGAGCG------------------------CGCGATCCTCGGATCGCGT-------------------------------------------------------------------------------------------------------------------------------------------------------------------CCGCTATCTAGAGGCACTGT-CGGT--GTGCGCAAGCCGAAGCGAGGAAGGCAATAACAGGTCTGTGATGCCCTTCGATGTTCTGGGCCGCACGCGCGCTACAATGACGAGGTCAGCGAGTCCGCCTCCACCGGAAGGTGTCGGGTAA-TCTTGCGAAACGTCGTCGTGATGGGGACAGAGCATTGAAATTATTGCTCTCGAACGAGGAATTCCTAGTAGGCGCGAGTCA-TCAGCTCGCGTCGATTACGTCCCTGCCCTTTGTACACACCGCCCGTCGCTACTACCGATTGGATGGTTTAGTGAGAGCTCGGGACCGGCCCGGGCGGCCCCCTCGCGGGGG--CCGCCCCGCGGTCGGGAACCTGGTCGAACTTGATCATTTAGAGGAAGTAAAAGTCGTAACAAGGTTTCCGTAGGTGAACCTGCGGAAGGATCATTA

EU272630_Polypodium_hydriforme_Russia ---------------------AGCGATATGCTCGTCTCAAAGT-TTAAGCCATGCATGT-CTCAGTACACGCCC-CTGTACGGTGAAACTGCGAACGGCTCATTACATCAGTTATCGTGTCCTCGATCGTTCCTTACCACATGGATACCCGTGGTAATTCTAGAGCTAATACATGCGCCCAGTC---CCGAC------CGCTTGCCGGAAGGGATGTATTTATTAGATTTTCAGACCATGACGG------------GGCAACCCGTGCGTTGGTGATTCATGGTAACTGCTCGGATCGCACGGCCTCGCGCCGGCGACGCCTCGTTCCAATTTCTGCCCTATCAACTGACGATGGTACGGTAGTGGCCTACCATGGTCGTAACGGGTGACGGAGAATCAGGGTTCGGTTCCGGAGAGGGAGCCCGAGAAATGGCTACCACTTCCACGGAAGGCAGCAGGCGCACACATTGCCCAATCCCGACACGGGGAGGCAGTGACAAGAAATAACGGTGCGTCGGCTTAGGCC-GTCGCAACCGGAATGAGTACGACCCAAATCCTCTAACGAGGATCCATTGGAGGGCAAGT-CTGGTGCCAGCAGCCGCGGTAATTCCAGCTCCAACAGTGTATGCTAATGTTGCTGCAGTTAAAAAGCTCGTAGTTGGATCGCAGGCGGTCGACGTCGAG------------GTGCACTACATGCG--------------------------------------------------------------------CTACCTCGCGTCGTCGC------------CTTATCCGCGGAGGCCGCGCGGTGCTCTTGATCGAGCGCCGGCCGGTCTACCCGGGACGTCACCTTGAGGAAACTAGAGTGTTCAAGGCAGGTGTCTCGCCCTGAATACC-GCAGCATGGAATGACACGCGGGAC--------------------------------------------------TGTGCGACGCGCAAGCGTCTCGGTC--CGGTGCGCGCGTTGGTCTGACGGACCGCAGTGACGGTTAAGAGGGACGGTCGGGGGCATTCGTATTTCGTCGTGAGAGGTGAAATTCTTAGACCGACGAAAGACGGACAAGTGCGAAAGCGTTTGCCAAGAACGTTTTCATTAATCAAGAACGAAAGTTAGAGGATCGAAGACGATCAGATACCGTCGTAGTTCT-AACCATAAACGATGCCGACCAGGGATCGGCGGGCCGT----GTTCATCGAGCT-CGTCGGCACCTCCCGGGAAACCTGAGTGTTTGGGTTCCGGGGGGAGTATGGTCGCAAGACTGAAACTTAAAGGAATTGACGGAAGGGCACCACCAGGAGTGGAGCCTGCGGCTTAATTTGACTCAACACGGGGAACCTCACCAGGTCCGGACATGACGAGGATTGACAGACTGACCGCTCTTTCTCGATCTCATGGTTGGTGGTGCATGGCCGTTCTTAGTTGGTGGAGCGATTTGTCAGGTTAATTCCGATAACGAACGAGACCTTATCCGGCTAGTTGAGCG------------------------CGCGATCCTCGGATCGCGT-------------------------------------------------------------------------------------------------------------------------------------------------------------------CCGCTATCTAGAGGCACTGT-CGGT--GTGCGCAAGCCGAAGCGAGGAAGGCAATAACAGGTCTGTGATGCCCTTCGATGTTCTGGGCCGCACGCGCGCTACAATGACGAGGTCAGCGAGTCCGCCTCCACCGGAAGGTGTCGGGTAA-TCTTGCGAAACGTCGTCGTGATGGGGACAGAGCATTGAAATTATTGCTCTCGAACGAGGAATTCCTAGTAGGCGCGAGTCA-TCAGCTCGCGTCGATTACGTCCCTGCCCTTTGTACACACCGCCCGTCGCTACTACCGATTGGATGGTTTAGTGAGAGCTCGGGACCGGCTCGGGTGGCCTCCTCGCGGTGG--CCGCCCCGCGGTCGGGAACCTGGTCGAACTTGATCATTTAGAGGAAGTAAAAGTCGTAACAAGGTTTCCGT-------------------------

;

end;

BEGIN PAUP;

Exclude [Positions with over 0.5 missing data]

44 60 75 136 141 191-198 245-259 348-349 527 587 692-717 738-780 784 798-801 829 906 909 920 945-994 1020 1040-1041 1217 1255-1259 1543 1556-1579 1599-1717 1724-1751 1787-1788 1883-1885 1910 1915 1992 2103-2107;

Exclude [ambiguous positions excluded by Guidance]

61 62 73 74 75 76 77 78 79 80 81 132 133 134 135 137 138 139 140 141 177 178 179 180 181 182 183 184 185 186 187 188 189 190 191 192 199 200 201 202 203 204 205 206 207 208 209 210 211 212 213 214 215 241 242 243 244 245 247 248 249 250 251 257 258 259 260 261 262 263 264 265 266 267 268 269 270 288 289 290 302 303 304 305 306 350 351 352 515 516 517 518 519 520 521 522 523 524 525 526 528 529 588 674 675 676 677 678 679 680 681 682 683 684 685 686 687 688 689 690 691 692 693 694 695 696 697 698 699 700 701 702 703 704 705 706 707 708 709 710 711 712 713 714 718 719 720 721 722 723 724 725 726 727 728 729 730 731 732 733 734 735 736 737 738 739 740 741 750 751 752 753 754 755 756 757 758 759 760 761 762 763 764 765 766 767 768 769 770 771 772 773 774 775 776 777 778 779 780 781 782 783 784 785 786 787 788 789 790 791 792 793 794 795 796 797 799 800 801 802 803 804 805 806 807 808 809 810 811 812 813 814 815 816 817 818 819 820 821 822 823 824 825 826 827 828 830 831 832 833 834 835 836 837 838 839 840 841 842 843 844 845 846 847 906 907 908 918 919 921 938 939 940 941 942 943 944 945 946 947 948 949 950 951 952 953 954 955 956 959 960 961 962 963 964 965 966 967 968 969 970 971 972 973 974 975 976 977 978 979 980 981 982 983 984 985 986 987 988 989 990 991 993 994 995 996 997 998 999 1000 1001 1002 1003 1004 1005 1006 1007 1008 1009 1010 1011 1012 1013 1014 1015 1016 1017 1018 1019 1021 1022 1023 1024 1025 1026 1027 1028 1029 1030 1031 1032 1033 1034 1035 1036 1037 1038 1039 1040 1042 1043 1218 1252 1253 1254 1256 1257 1259 1260 1261 1262 1263 1264 1265 1266 1267 1268 1269 1270 1272 1273 1274 1275 1276 1277 1278 1279 1280 1281 1282 1283 1284 1285 1286 1287 1288 1289 1290 1291 1292 1293 1294 1295 1544 1545 1546 1547 1548 1549 1550 1551 1552 1553 1554 1555 1572 1573 1574 1575 1576 1577 1578 1579 1580 1581 1582 1583 1584 1585 1586 1587 1588 1589 1590 1591 1592 1593 1594 1595 1596 1597 1598 1600 1601 1602 1603 1604 1605 1606 1607 1608 1609 1610 1611 1612 1613 1614 1615 1616 1617 1618 1619 1620 1621 1622 1623 1624 1625 1626 1627 1628 1629 1630 1631 1632 1633 1634 1635 1636 1637 1638 1639 1640 1641 1645 1646 1647 1648 1649 1650 1651 1652 1653 1654 1655 1656 1657 1658 1659 1660 1661 1662 1663 1664 1665 1666 1667 1668 1669 1670 1671 1690 1691 1692 1693 1694 1695 1696 1697 1698 1699 1700 1701 1702 1703 1704 1705 1706 1707 1708 1710 1711 1712 1713 1714 1715 1716 1717 1718 1719 1720 1721 1722 1723 1724 1725 1750 1751 1752 1753 1754 1755 1756 1757 1758 1759 1760 1761 1762 1763 1764 1765 1766 1767 1782 1783 1784 1785 1786 1789 1790 1791 1792 1793 1886 1887 1888 1889 1890 1891 1892 1893 1894 1895 1896 1897 1898 1899 1900 1901 1902 1903 1904 1905 1906 1907 1908 1909 1911 1912 1913 1914 1916 1917 1918 2085 2086 2087 2088 2089 2097 2098 2099 2100 2101 2102 2103 2106 2107 2108 2109 2110 2111;

end;
