# Supplementary material for: Evolution of myxozoan mitochondrial genomes: insights from myxobolids
Source: BMC Genomics. 2024 Apr 22;25:388. doi: 10.1186/s12864-024-10254-w (PMC11034133; doi:10.1186/s12864-024-10254-w)
Supplement: Supplementary file 7 — Supplementary Material 7 [file 12864_2024_10254_MOESM7_ESM.docx]

#NEXUS

[Additional file 7 – 28S rRNA sequence alignment used for phylogenetic reconstructions. Nucleotide sequence alignment, in Nexus format]

begin data;

dimensions ntax=13 nchar=4259;

format datatype=dna missing=? gap=-;

matrix

Kudoa_iwatai_Israel TGACCTCAAATCAGGCAAGACTACCTGCTGAACTTAAGCATATCAGTAAGCAGAGGAAAAGAAAACAACTGTGATTCCCTTAGTAACTGCGAGTGAAGCGGGATAAGCCCATCGTTTAATCTTCTTGG--------ACCTAGTTCAAGACGAAATGTAACGGTATGGATAGGTTCTCTACTAAGAATGTC--TAGTCAAAGTCCTCCGGATTGAGGTGCCATAGAGGGTGACAGCCCCGTATGTGACTAGAT----AAACTTAGGTATGTGACCAAATCCGAGAGTCGGGTTGTTTGGGACTGCAACCTAAAGTAGGTGGTAAACTCCACCCAAGGCTAAATATAGACACGAAACCGATAGCGAACAAGTACTGTGAGGGAAAGTTGAAAAGAACTCTGAAAAGAGAGTTAAAAGTACGTGAAACCGCTAACGGGGAAGCGGAAGGCAGTGTCGA-GATTCTGTGAGTGAGATATTATATGTTTAAGGGTATATGTTGTTGTTGATAATGTGCCA-ATATACATGTATTATCTAGCTTGGCAGAATGCGT--------------CAGATCTATTGGTAT-TAACATGATTAAATTATTTGAT--------TGGCTTGTACGA---CGCCTATA------------TACTTGTATATGGGTCCGTATTTGTAATAGAT----------AATCCGTCATGTGTACTAATATGTATG----TGATGAAGCGTTAGTGATTTATTATCGGTTGAGAGTTTG---------TAGTGCTGTGTATTGATTGCAGTGTAGTTA-GTATGGAGAAACTACA-AAATTTTCAACAACTAAT--GCTAGTATCTGACG-AGATACCTAACCTTCCGACCCGTCTTGAAACACGGACCAAGGAGGCAGAATCTAGAGCAAGTTGGAGGG--------------------TGTA-AAACCCGACGGCGTAGTGAAAGCAATAGATGAGATCTACTAAC-----------------TTTGGTTGGTAGCGCATCATCGGCCAGTCATT---------AT----ACAATGATTGAGTTAGAGCTCTAGGTGCTGGACCCGAAAGATGGTGAACTATGCGTGAGCAGGGTGAAGCCAGAGGAAACTCTGGTGGAAGCTCGAAGCGATTCTGACGTGCAAATCGATCGTTTGACTTGCGTATAGGGGCGATAGACTAATCGAACCATCTAGTAGCTGGTTCCCTTCGAAATATCCCTCAGGATCGCTGAGACAAGTG---ATAAACAGTTTC-ATCGGGTAGAGCGAATGATTAGAGGAATCG-GGGATATAT-TACTCTCGACCTATTCTCAAACTATCAACCGGTGAGAACATTGT---------------TTTTCTTGTTTG-----AAACAATG--------TGTATGAATGTTGTTGTCTCAAGTGGGCCATTTTTGGTAAGCAGAACTGGCGATGCGGGATGAACCGAGAGTCGAGCTAAGGTGCCTAAGGACACGTTGATA-GATACCACAAAAGGAGTCGGTCGATAAAGACAGCAGGGCGGTGGCCCTGAAAGTAGGAATCCGCTAAGGAG-TGTCTAACAACTCACCTGCCGAATCGACCGGCCCTGAAAATGGATGGCGCTGAAGCGTGTCACCGATGCTCGACCTCTACC--ACGAGAGATGT------TGGTAGAGGGTAGAAGGGCGCAGCGGTAGCGATAAAGTACAAAGTGTAAACTTGTATGGAGCTGCCGTTGGTGTAGATCTTGGTGGTAGTAGCAAT-TATAAAAGTGAGTAAAAACCTTTTAGGCCGAAGTGGAGAAGGGTTCCACGTGAACAGCAGTTGGACGTGGGTTAGTCGGTCCTAAGCGGTTGCCTAACGGCTATTTCAATGCTGACTTGGTTATTACGTG--------GTAATTCATGTAAAAAGTGATGAACTGCGTAGTGTGACGT-----GGGTCAGAATTCCGCGAAAGGGAATCAGGTTAATATTCCTGAACCGG--GCGATGGAGACGCTT---------------------TTTGTCAGTATTATTC----GTAAGAGTAGTGCTGCAATTGGCTTAGCGGTAACGCAAACAATCTCGGAGACGTTGATATCTATGGCGGGGAGAATTATCTTTTCTGTATAACAAGTCAA-CTACCTTGGAATCGAGTCATTCGGAGATAAGGTAATAAGCTTGGAAGAGCACCGCAATTTTATGCAGTGTCCGTACTATAGATATCGGCCCTTGAAAATCCGAGGGAGTG-----GTTAAATTTCACGCCCGTCCGTACCGA-ATCCGCATCAGGTCTCCAAGGTGTGCAGCCTCTGGCA-CTGGAACAATGTAGGTAAGGGAAGTCGGCAAAATAGATCCGTAACTTTGGGAAAAGGATTGGCTCTAAGGGCCGGGCCAATCGAGCTAGTATTGCCTAGGCTAGTGGGGGTTGAACGGGGT-CTATCTAGATGCAT-------------GTAAAAGTGTTATT--TGGATGGATCTGGTGAAGC-AAACGCTGGCCCTGGAATACCTGTGTGAACTTGTATTGGCAGATTGATTGTTGGTGTAGCAGTT--TGCTTAATTGTGGATTGTTATGCTGGCAATTCGGGCTGTTGATAGGGTTCTTTACGAGTGGCAAGCATCGGTCAGCTTAGAACTGTTACTAACTAGGGGAATCCGACTGTTTAATTAAAACAAAGCATTGCGATGGCCGGAAA-CGGTGTTGACGCAATGTGATTTCTGCCCAGTGCTCTGAATGTCAAAGTGCGGAAACGCAACCAAGCACGGGTAAACGGCGGGAGTAACTATGACTCTTTGAAGGTAGCCAAATGCCTCGTCATCTAATTAGTGACGCGCATGAATGGATCAACGAGATTCCCACTGTCCCTACCTACCATCCAGCGAAACCACAGTCAAGGGAACGGGCTTGACTGAGACAGCGGGGAAAGAAGACCCTGTTGAGCTTGACTCTAGTTTGATTTTGTGAGAAGACATGAGGGGTGTAG-AATAAGTGGGAGGTTAA--------TATTATTATTAGCCGACAGTGAAATACCACTACTTTCATTGTTTTCACACTAAGTCGGTGGCACGGGAGATACTCGTTT------------------------------------------------------------------------------------CGGCGGGTGGATT----------------------------CGTGGCTATAAGGCA----GACTG------------------------------------------------------------------------ATCTGTTGACC----------------------------------------CATTACCGATGACAGAATCAGATGGAGAGTTTGACTGGGGCGGTACATCCTTAAAAATGCAACGAGGGTGTCCAAAGGCGAGCTCACCGTGGACAGAAACCACGGGTAGAGCAAAAGGGCAAAAGCTCGCTTGATTCCGATTTTCAGTACGAATACGAACCACGAAAGTGTGGCCTATCGATCCCTTGA---TCACCCAGAGTTTGGGGACAGGGGTGTCAGAAAAGTTACCACAGGGATAACTGGCTTGTGGCGGCCAAGCGTCCATAGCGACGTCGCTTTTTGATCCTTCGATGTCGGCTCTTCCTATCATTGGGTAGCAGCATACTCAAAGTGTTGGATTGTTCACCCACCAACAGGGAACGTGAGCTGGGTTTAGACCGTCGTGAGACAGGTTAGTTTTACCCTACTGTCTCTA-----GCACTGTAACAGTAGTCCTGTTTAGTACGAGAGGACCCGCAGGACCGGATAAACGG-TGAAGCATTTGGTTGATAGGCCAGTGATGCAATGCTGTCATCCGCTGGATAATGGCTGAAAGCCTC-TAAGCCAGAAACCAGTCTG----AAACAGTGATTAATTCCAGCCG--TAGCAGTGAACCTATACCGTT------------GAGCGTTATGCTTAATCGGATGATCCAAGCTAA-----CTTGATTGAATGTTTGAGTTGTCAACAA-----------------ATAGCAGTGATAATGCATT--GGCTGGGTTT-TAAGTAACATGTATACGACAT--GTAGTCGTCAGGGTACTGTAAGAGTGCGAGTGGACATGAAGCCACGAGACTCTGAGGTTCATCCCATGACGCAA-AACTTTG

LC066366_Kudoa_iwatai_Japan TGACCTCAAATCAGGCAAGACTACCTGCTGAACTTAAGCATATCAGTAAGCAGAGGAAAAGAAAACAACTGTGATTCCCTTAGTAACTGCGAGTGAAGCGGGATAAGCCCATCGTTTAATCTTCTTGG--------ACCTAGTTCAAGACGAAATGTAACGGTATGGATAGGTTCTCTACTAAGAATGTC--TAGTCAAAGTCCTCCGGATTGAGGTGCCATAGAGGGTGACAGCCCCGTATGTGACTAGAT----AAACTTAGGTATGTGACCAAATCCGAGAGTCGGGTTGTTTGGGACTGCAACCTAAAGTAGGTGGTAAACTCCACCCAAGGCTAAATATAGACACGAAACCGATAGCGAACAAGTACTGTGAGGGAAAGTTGAAAAGAACTCTGAAAAGAGAGTTAAAAGTACGTGAAACCGCTAACGGGGAAGCGGAAGGCAGTGTCGA-GATTCTGTGAGTGAGATGTTATATGTTTATGGGTATATGTTGTTGTTGATAATGTGCCA-ATATACATGTATTATCTAGCTTGGCAGAATGCGT--------------CAGATCTATTGGTAT-TAACATGATTAAATTATTTGAT--------TGGCTTGTACGA---CGCCTATATACTTGTACTTGTACTTGTATATGGGTCCGTATTTGTAATAGAT----------AATCCGTCATGTGTACTAATATGTATG----TGATGAAGCGTTAGTGATTTATTATCGGTTGAGAGTTTG---------TAGTGCTGTGTATTGATTGCAGTGTAGTTA-GTATGGAGAAACTACA-AATTTTTCAACAACTAAT--GCTAGTATCTGACG-AGATACCTAACCTTCCGACCCGTCTTGAAACACGGACCAAGGAGGCAGAATCTAGAGCAAGTTGGAGGG--------------------TGTA-AAACCCGACGGCGTAGTGAAAGCAATAGATGAGATCTACTAAC-----------------TTTGGTTGGTAGCGCATCATCGGCCAGTCATT---------AT----ACAATGATTGAGTTAGAGCTCTAGGTGCTGGACCCGAAAGATGGTGAACTATGCGTGAGCAGGGTGAAGCCAGAGGAAACTCTGGTGGAAGCTCGAAGCGATTCTGACGTGCAAATCGATCGTTTGACTTGCGTATAGGGGCGATAGACTAATCGAACCATCTAGTAGCTGGTTCCCTTCGAAATATCCCTCAGGATCGCTGAGACAAGTG---ATAAACAGTTTC-ATCGGGTAGAGCGAATGATTAGAGGAATCG-GGGATATAT-TACTCTCGACCTATTCTCAAACTATCAACCGGTGAGAACATTGT---------------TTTTCTTGTTTG-----AAACAATG--------TGTATGAATGTTGTTGTCTCAAGTGGGCCATTTTTGGTAAGCAGAACTGGCGATGCGGGATGAACCGAGAGTCGAGCTAAGGTGCCTAAGGACACGTTGATA-GATACCACAAAAGGAGTCGGTCGATAAAGACAGCAGGGCGGTGGCCCTGAAAGTAGGAATCCGCTAAGGAG-TGTCTAACAACTCACCTGCCGAATCGACCGGCCCTGAAAATGGATGGCGCTGAAGCGTGTCACCGATGCTCGACCTCTACT--ACGAGAGATGT------TGGTAGAGGGTAGAAGGGCGCAGCGGTAGCGATAAAGTACAAAGTGTAAACTTGTATGGAGCTGCCGTTGGTGTAGATCTTGGTGGTAGTAGCAAT-TATAAAAGTGAGTAAAAACCTTTTAGGCCGAAGTGGAGAAGGGTTCCACGTGAACAGCAGTTGGACGTGGGTTAGTCGGTCCTAAGCGGTTGCCTAACGGCTATTTCAATGCTGACTTGGTAATTACGTG--------GTGATTCATGTAAAAAGTGATGAACTGCGTAGTGTGACGT-----AGGTCAGAATTCCGCGAAAGGGAATCAGGTTAATATTCCTGAACCGG--GCGATGGAGACGCTT---------------------TTTGTCAGTATTATTC----GTAAGAGTAGTGCTGCAATTGGCTTAGCGGTAACGCAAACAATCTCGGAGACGTTGATATCTATGGCGGGGAGAATTATCTTTTCTGTATAACAAGTCAA-CTACCTTGGAATCGAGTCATTCGGAGATAAGGTAATAAGCTTGGAAGAGCACCGCAATTTTATGCAGTGTCCGTACTATAGATATCGGCCCTTGAAAATCCGAGGGAGTG-----GTTAAATTTCACGCCCGTCCGTACCGA-ATCCGCATCAGGTCTCCAAGGTGTGCAGCCTCTGGCA-CTGGAACAATGTAGGTAAGGGAAGTCGGCAAAATAGATCCGTAACTTTGGGAAAAGGATTGGCTCTAAGGGCCGGGCCAATCGAGCTAGTATTGCCTAGGCTAGTGGGGGTTGAACGGGGT-CTGTCTAGATGCAT-------------GTAAAAGTGTTATT--TGGATGGATCTGGTGAAGC-AAACGCTGGCCCTGGAATACCTGTGTGAACTTGTGTTGGCAGATTGATTGTTGGTGTAGCAGTT--TGCTTAATTGTGGATTGTTATGCTGGCAATTCGGGCTGTTGATAGGGTTCTTTACGAGTGGCAAGCATCGGTCAGCTTAGAACTGTTACTAACTAGGGGAATCCGACTGTTTAATTAAAACAAAGCATTGCGATGGCCGGAAA-CGGTGTTGACGCAATGTGATTT--------------------------------------------------------------------------------------------------------------------------------------------------------------------------------------------------------------------------------------------------------------------------------------------------------------------------------------------------------------------------------------------------------------------------------------------------------------------------------------------------------------------------------------------------------------------------------------------------------------------------------------------------------------------------------------------------------------------------------------------------------------------------------------------------------------------------------------------------------------------------------------------------------------------------------------------------------------------------------------------------------------------------------------------------------------------------------------------------------------------------------------------------------------------------------------------------------------------------------------------------------------------------------------------------------------------------------------------------------------------------------------------------------------------------------------------------------------------------------------------------------------------------------------------------

Kudoa_septapunctata TGACCTCAACTCAGGCAAGACTACCTGCTGAACTTAAGCATATCAGTAAGCAGAGGAAAAGAAAACAACTGTGATTCCCTTAGTAACTGCGAGTGAAGCGGGAAAAGCCCATCGTTTAATCTTCCTGG--------ACCATGGACAGGACGAAATGTAACGGTATGGATAGGTTTTCTACCAACAAAGTC--TGGTCAAAGTCCTCCGGATTGAGGTGCCAAAGAGGGTGACAGCCCCGTGTGTGATCAGACTTGATATGTTGGGTATGTGACCGATTCCGAGAGTCGGGTTGTTTGGGACTGCAACCTAAAGTAGGTGGTAAACTCCACCCAAGGCTAAATATTGACACGAAACCGATAGCGAACAAGTACTGTGAAGGAAAGTTGAAAAGAACTCTGAAAAGAGAGTTAAAAGTACGTGAAACCGCTAACGGGGAAGCGGAAGGCAGTGTCGA-GATTGAGTGAGTGAGTCGGCTCAAACTCATTTC-AAATGAGATTGTGTGTTGAGTGGAATGTGAGTTTGAGTAGGCTAGCTTTGCTTGATGCGT--------------CAGGGCTATTGGCAC-TAAAGAGGTTAAATGGCCAGCAG----TTTTGGCTTTCACAGCCAATGATTTATTG---------------------TTGGCGTGGATGCGGCTGGT----------CATCCGTCTCTTGTGCTGATATGTGTG----TGTGGAAGCGTTGGTGATGTATTGTCGGTTGGTGTCGTG---------TGGTGGTTCGTTCTGGTCACAGTGTGGCTACGTGCGGACAAGTCATGTGGTAGTTCAACAACCAAT--GCTAGATCCTGGCG-TGATACCCAACTTTCCGACCCGTCTTGAAACACGGACCAAGGAGGCAGAATCTATAGCGAGTTGGAGGG--------------------TGGA-AAACCCGACGGCGCAGTGAAAGCGAGAGATGAGAACTGGGGGC-----------------CTCGGCTCTCAGTGCATCATCGGCCAGTCATC---------ATTTG---GGTGATTGAGTTTGAGCTTTAGATGCTGGACCCGAAAGATGGTGAACTATGCGTGAGCAGGGTGAAGCCAGAGGAAACTCTGGTGGAGGCTCGAAGCGATTCTGACGTGCAAATCGATCGTTAGACTTGCGTATAGGGGCGATAGACTAATCGAACCATCTAGTAGCTGGTTCCCTTCGAAATATCCCTCAGGATCGCTGAGACAAGTG---GGAGACAGTTTC-ATCGGGTAGAGCGAATGATTAGAGGAATCG-GGGATATAT-TACTCTCGACCTATTCTCAAACTTCCAACCGGTGAGAGTGCTG----------------TTTGCTTGATTATTTTTGAAATGGC--------ACGAGGAATGTTTTTGTCTCAAGTGGGCCATTTTTGGTAAGCAGAACTGGCGATGCGGGATGAACCGAGAGTTGAGCTAAGGTGCCTAAGGACACGTTGATA-GATACCACAAAAGGAGTCGGTCGATAAAGACAGCAGGACGGTGGCCCTGAAAGTAGGAATCCGCTAAGGAATTGTCTAAAAACTCACCTGCCGAATCGACCGGCCCTGAAAATGGATGGCGCTGAAGCGTGTCACCGATGCTCGACCTCTACC--ACGAGAGATGA------TGGTAGGGGGTAGAAGGGCGCGGCGGTAGCGAAAAAGTACAAAGTGTAAACTTGTATGGAGCTGCCGTCGGTGTAGATCTTGGTGGTAGTAGCAAT-TATAAAAGTG----AAAACCTTTTAGGCCGAAGTGGAGAAGGGTTCCACGTGAACAGCAGTTGGACGTGGGTTAGTCGGTCCTAAGCGGAAGCCTAACGG-TATTTTAAAGCCAACTT---------TTA--------CTTATT------------------TCGCAAGGGGTGATTG-----AGAGTTGGAATCCGCGAAAGGGAATCAGGTTAATATTCCTGAACCGG--GCAATGGAGACGCTGCTCTTTTATTG----------TTTGTTGATTTGATTGA---GTTGGTGAATAATAGAGAGCAGCTTAGCGGTAACGCAAGCAATCTCGGAGACGTTGACATCTATGGCGGGGAGAATTATCTTTTCTGCATAACAGGCAAA-CAACCTTGGAATCGAGTCGTTCGGAGATAAGGTAAAAAGCCTGGAAGAGCACCGCAACTAT-TGCAGTGTCCGCAGTGTAGGTGTCGGCCCTTGAAAATCCGAGGGAGTG-----GATAAATTTCATGCCCGTCCGTACCGA-ATCCGCATCAGGTCTCCTAGGTGAGCAGCCTCTGGCA-CTGGAACAATGTAGGTAAGGGAAGTCGGCAAAATAGATCCGTAACTTTGGGAAAAGGATTGGCTCTAAGGACCGGGCCAATCGAGCTAGTATTGCCTAGGCTAGTGGGGGTTTGACTGGGG-TTGTCTTGTGATGCTCTATCGTCAAAAAGTAGAGTGTTATTG-AGGCACACTTTGGTTATTT-CAACGCTGGCCATGGAATGCCGGCGTGAGTTTTGTTGTGTTGAGTAAATGT-----CAAAAGTT--TGTTCGAT------------------------------ATGGCGAGCTTCTTTACGAGTGGCAAGCATCGGTCAGCTTAGAACTGTTACCAACTTGGGGAATCCGACTGTTTAATTAAAACAAAGCATTGCGATGGCCCTAAT-GGGTGTTGACGCAATGTGATTTCTGCCCAGTGCTCTGAATGTCAAAGTGCGGAAACGCAACCAAGCACGGGTAAACGGCGGGAGTAACTATGACTCTTTAAAGGTAGCCAAATGCCTCGTCATCTAATTAGTGACGCGCATGAATGGATCAACGAGATTCCCACTGTCCCTACCTACCATCCAGCGAAACCACAGTCAAGGGAACGGGCTTGACTGAGACAGCGGGGAAAGAAGACCCTGTTGAGCTTGACTCTAGTTTGATTTTGTGAGAAGACATGAGGGGTGTAG-AATAGGTGGGAGGTTTT------ATTAATTGATGAGACCGTCGGTGAAATACCACTACCTTTATCGTTTTCACACTAAGTCGGTTGCACGGGAAATACTTGTCT-----------------------CTTG---AA---------------------------------------------------GAGGCAGGTGGATT----------------------------CGTGGCTATAAGACA----GACTG------------------------------------------------------------------------ATCTGTTGACC----------------------------------------CATCGCCGATGACAGAATCAGATGGGGAGTTTGACTGGGGCGGTACATCCTTAAAAATGCAACGAGGGTGTCCAAAGGCGAGCTCATCGTGGACAGAAACCACGAGTAGAGCAAAAGGGCAAAAGCTCGCTTGATTCCGATTTTCAGTACGAATACGAACCACGAAAGTGTGGCCTATCGATCCCTTGA---TCGCCCAGAGTTTGGGGACAGGGGTGTCAGAAAAGTTACCACAGGGATAACTGGCTTGTGGCGGCCAAGCGTCCATAGCGACGTCGCTTTTTGATCCTTCGATGTCGGCTCTTCCTATCATTGGGTAGCAGTATACTCAAAGTGTTGGATTGTTCACCCACCAACAGGGAACGTGAGCTGGGTTTAGACCGTCGTGAGACAGGTTAGTTTTACCCTACTGTCTCTTTA---GCGCTGTGACAGTAGTCCTGCTTAGTACGAGAGGACCCGCAGGACCGGATAAACGG-TGAAGCATTTGGCTGATAGGCCAGAGATGCAATGCTGTCATCCGCTGGATAATGGCTGAAAGCCTC-TAAGCCAGAAACCAGTCTG----AAGCAGTGAAAGACACTGGCCG---AGCAGTGAACAAATACGTGT------------------CATTTTTATTTATGAGAGTTGACATGA-------------------TGTTCCATTAATCA-----------------ATGTCATGAGAAATGTGTT-------GAGTT-ATATTTTATTAAAGTGGAAGT--GCTAAGGCCGGGTTAC------------------------------------------------------------------

Kudoa_hexapunctata TGACCTCAACTCAGGCAAGACTACCTGCTGAACTTAAGCATATCAGTAAGCAGAGGAAAAGAAAACAACTGTGATTCCCTTAGTAACTGCGAGTGAAGCGGGAAAAGCCCATCGTTTAATCTTCCCGG--------ACCATGGACGGGACGAAATGTAACGGTATGGATAGGTTCTCTACCAACTATGTC--TGATCAAAGTCCTCCGGATTGAGGTGCCAAAGAGGGTGACAGCCCCGTGTGTGATTGGAT----GGCGTTGGGTATGTGACCGATTCCGAGAGTCGGGTTGTTTGGGACTGCAACCTAAAGCAGGTGGTAAACTCCACCCAAGGCTAAATATTGACACGAAACCGATAGCGAACAAGTACTGTGAAGGAAAGTTGAAAAGAACTCTGAAAAGAGAGTTAAAAGTACGTGAAACCGCTAACGGGGAAGCGGAAGGCAGTGTCGA-GATTGAGTGAGTGAGTTGGTTCAAGTGCGTTTG-GTGTGTCG-TGTTGGCGGTGTGTAATGTGCGCTTGGATCGGCTAGCTTTGCTTGATGCGT--------------CAGGACTATTGGTG--TGCGACGGTTCAATCGTCAGCA--------TGGCTGTCACGG---AACACTCAAAT---------------------GTTTCGTGGTTGCGGCTGGT----------GATCCGCCTCGTGTGCTGATATGTATG----TGATGGAGCGTTGGTGATGTATGGTCGGTTGGTGCTGTG---------CGGCGTTTCGTGCTGGTCACATTGTGGCTACGTGCGGAAAAGTCGTGTGGTAGTTCAACAACCAGT--GCTAGCTCCTGACG-TGATACCTAACTTTCCGACCCGTCTTGAAACACGGACCAAGGAGGCAGAATCTAGAGCGAGTTGGAGGG--------------------TGCA-AAACCCGACGGCGCAGTGAAAGCGAGAGATGAGATCCGGGGGT-----------------CTCGGCCCTCGGAGCATCATCGGCCAGTCACT----------------CTGTGATTGAGTTTGAGCTGTAGATGCTGGACCCGAAAGATGGTGAACTATGCGTGAGCAGGGCGAAGCCAGAGGAAACTCTGGTGGAGGCTCGAAGCGATTCTGACGTGCAAATCGATCGTTTGACTTGCGTATAGGGGCGATAGACTAATCGAACCATCTAGTAGCTGGTTCCCTTCGAAATATCCCTCAGGATCGCTGAGACAAGTG---GGAGACAGTTTC-ATCGGGTAGAGCGAATGATTAGAGGAATCG-GGGATATAT-TACTCTCGACCTATTCTCAAACTTCCAACCGGTGAGAGTGCTG----------------TTTACTTGATTG-----AAACGGGC--------ACGCGGAATGTCTTTGTCTCAAGTGGGCCATTTTTGGTAAGCAGAACTGGCGATGCGGGATGAACCGAGAGTTGAGCTAAGGTGCCTAACGACACGTTGATA-GATACCACAAAAGGAGTCGGTCGATAAAGACAGCAGGGCGGTGGCCCTGAAAGTAGGAATCCGCTAAGGAATTGTCTAAAAACTCACCTGCCGAATCGACCGGCCCTGAAAATGGATGGCGCTGAAGCGTGTGACCGATGCTCGACCTCTACC--ACGAGAGATGT------TGGTAGGGGGTAGAAGGGCGCGGCGGTAGCGACAAAGTACAAAGTGTAAACTTGTATGGAGCTGCCGTCGGTGTAGATCTTGGTGGTAGTAGCAAT-TATAAAAGTG----AAAACCTTTTAGGCCGAAGTGGAGAAGGGTTCCACGTGAACAGCAGTTGGACGTGGGTTAGTCGGTCCTAAGCGGTTGCCTAACGGCTAGTGAAAAGCCAACTT---------ATG--------GTCGCACTT---------------GTGTGGCTAAATATAA-----TAGGTTGGAATCCGCGAAAGGGAATCAGGTTAATATTCCTGAACCGG--GCTATGGAGACGCTGCCTTGTGCGTT----------ATTGCCAATTC---------GTTGGTGGTGGCGTTGGGGTAGCTTAGCGGTAACGCAAACAATCTCGGAGACGTTGACATCTATGGCGGGGAGAATTATCTTTTCTGCATAACAGGTAAA-TCACCTTGGAATCGAGTCATTCGGAGATAAGGTAAAAAGCCTGGAAGAGCACCGCAACTAT-TGCAGTGTCCGCAGTGTAGGTGTCGGCCCTTGAAAATCCGAGGGAGTG-----GATAAATTTCATGCCCGTCCGTACCGA-ATCCGCATCAGGTCTCCTAGGTGAGCAGCCTCTGGCA-CTGGAACAATGTAGGTAAGGGAAGTCGGCAAAATAGATCCGTAACTTTGGGAAAAGGATTGGCTCTAAGGGCCGGGCCAATCGAGCTAGTATTGCCTAGGCTAGTGGGGGCTTGACTGGGT-TTGCTC-------------------------GGGGGCAACTC-TGAGTAGGCTTGGTCTTGC-AAACGCTGGCCATGGAATGCCGGCGTGAATTTCGGTCGGCTGAGCGGCCGC-------AAGGTT--TGTTCAGT-------------------------------TGATGGGGTTCTTTACGAGTGGCAAGCATCGGTCAGCTTAGAACTGTTACCAACTTGGGGAATCCGACTGTTTAATTAAAACAAAGCATTGCGATGGCCGGAAA-CGGTGTTGACGCAATGTGATTTCTGCCCAGTGCTCTGAATGTCAAAGTGCGGAAACGCAACCAAGCACGGGTAAACGGCGGGAGTAACTATGACTCTTTAAAGGTAGCCAAATGCCTCGTCATCTAATTAGTGACGCGCATGAATGGATCAACGAGATTCCCACTGTCCCTACCTACCATCCAGCGAAACCACAGTCAAGGGAACGGGCTTGACTGAGACAGCGGGGAAAGAAGACCCTGTTGAGCTTGACTCTAGTTTGATTTTGTGAGAAGACATGAGGGGTGTAG-AATAGGTGGGAGATTGT------------TTACGCAGTCGACAGTGAAATACCACTACCTTTATCGTTTTCACACTAAGTCGGTTGCACGGGAAATACTTGTCC-----------------------CTTT---GA---------------------------------------------------GGGGCAGGTGGATT----------------------------CGTAGCTATAAGACA----GACTG------------------------------------------------------------------------ATCTGTTGACC----------------------------------------CATCGCCGATGACAGAATCAGATGGGGAGTTTGACTGGGGCGGTACATCCTTAAAAATGCAACGAGGGTGTCCAAAGGCGAGCTCATCGTGGACAGAAACCACGAGTAGAGCAAAAGGGCAAAAGCTCGCTTGATTCCGATTTTCAGTACGAATACGAACCACGAAAGTGTGGCCTATCGATCCCTTGA---TCTCCCAGAGTTTGGGGACAGGGGTGTCAGAAAAGTTACCACAGGGATAACTGGCTTGTGGCGGCCAAGCGTCCATAGCGACGTCGCTTTTTGATCCTTCGATGTCGGCTCTTCCTATCATTGGGTAGCAGTATACTCAAAGTGTTGGATTGTTCACCCACCAACAGGGAACGTGAGCTGGGTTTAGACCGTCGTGAGACAGGTTAGTTTTACCCTACTGTCTCTTTA---GCGCTGTGACAGTAGTCCTGCTTAGTACGAGAGGACCCGCAGGACCGGATAAACGG-TGAAGCATTTGGCTG----------------------------------------------------------------------------------------------------------------------------------------------------------------------------------------------------------------------------------------------------------------------------------------------------------------------------------------------------------------

Enteromyxum_leei GGACCTCAACTCAGGCACGATCACCCGCCGAACTTAAGCATATCAGTAAGCGGAGGAAAATAAAACAACAGTGATTTCCCTAGTAACGGCGAGTGAAGCGGAAAAAGCCCAGCATTTAATCTCTCTC--------------TTGTAGAGCGAATTGTAAT-GTACGAACTAGTACTTATGTAAACAGGGCAACTACCAAAGTTTTCAGGAATGAAGCGCCACAGAGGGTAATAGCCCCGTATCTGGTAGTTGTC-----TATGCCCCTAATGCTAGATTCTCGAGTCGGACTGTTTGGGATTGCAGTCTAAA-CGGGTGGTAGACTCCATCCAAGGCTAAATATAAGCACAAAACCGATAGCGAACAAGTACCGTGAGGGAAAGTTGAAAAGAATTCTGAAAAGAGAGTTAAAAGTACGTGAAACCGTTAACAGGGAAGCGGAAGGCTGTGTCGA-GACGGTACAAGGCGTGGTGACTAATGTCTTTT----------------------------TTAAAGATTCATTAGGTAGCTTTGTCTTGTACGTGCG----------TCAGACATGTCGTT---------------------------------TCCTGCAGTCAA---AGTAGTCATGGTTGTAACTTCCTTC-------GGGTTGTTGCTGCGGTCTGC----------ATTGGATTGTCGGAGATGGCATATGAT------TGCCTATGTTAATCATATGTAAGTATTTCTTAC-------------TAGTGTTGCGT-CTAATTGTCGTAGGGTTA-GTAAAAAGTGCTT-----------------CTGATTAGCGTAGGTCTGGCG-AGATACCTAGTCTTCCGACCCGTCTTGAAACACGGACCAAGGAGGCAGAAGCTAGAGCGAGTTGGAGGG--------------------TGCA-AAACCCGACGGCAAAGTGAAAGCAATAAATGAGAC-----------------------TGCGTTTAGGCGTGGGCATCATTGACCAGTACTT-----------------TAGTACTGAGTTAGAGCTCTAGCTGCTGAACCCGAAAGATGGTGAACTATGCATGAGCAGGACAAAGTCAGGGGAAACTCTGATGAAAGTCCGTAGCGATTCTGACGTGCAAATCGATCGTTAGACTTGTGTATAGGGGCGATAGACTAATCGAACCATCTAGTAGCTGGTTCCCTTCGAAATATCCCTCAGGATCGCTGAGACAAG-----TTAGACAGTTTC-ATCGGGTAGAGCTAATGATTAGAGGCATTG-GGGCCGTGA-TGGCCTCAACCTATTCTCAAACTCCCAACTGGTGATTGCTTTT----------------TTTGCTTAATTG-----AATTAAAG---------CTTTGAATGTCTTTGTCTCAAGTGGGCCATTTTTGGTAAGCAGAACTGGCGATGCGGGATGAACCGAGAGCCAAGCTAAGGTGCCTAATAGCACGCTAATA-GATACCACAAAAGGTGTCGGTTGATCAAGACAGCAGGACGGTGTCCCTAGTAGTAGGAATCCGCTAAGGAT-TGTGTTACAACTCACCTGCCAAATTAACCGGCCCTGAAAATGGATGGCGCTTAAGCGTGCAACCGATGCTTGGCCTTTACT--ACTTGAAATGT------TAGTAAAGGGTAGAAGGGCGCAGCGGTAGTGATAAAGTGTAAAGCGTGAGCTTGCATGAAACTGCCGTTGGTGTAGATCTTGGTGGTAGTAGCAAC-TATAAAAGTGAGAAATAACCTTTTAGGCCGAAGTGGAGAAGGGTTCCACGTGAACAGCAGTTGGACGTGGGTTAGTCGGTCCTAAGCAGTTGCCTAACGGCTATTTCAACGGTGATTT------------------------------------------------------------------AATCACAGTTCTGCGAAAGGGAATCAGGTTAATATTCCTGAACCAA--GTGTCGGAGAT-------------------------------------------------GTGTGTACTTGTTATGCACTAGACGGTAACGTAAACAATCTCAGAGACGCCAACAGTTACTGTGGGGAGAATTATCTCTTCTCTATAACAAGCCAA--AGCCTTGGAATTGAGTCATTCAGAGATGAGGCCATAAGCTTGGAAGAACACTGCAATTAA-TGCAGTGTCCGCAACGTAACTGTTGGACCTTGAAAATCTGAGGGCGTG-----GTTAATTTTCGCACTTGGCCGTACCAA-ATCCGCATCAGGTCTCCAAGGTGTGTAGCCTCTGGCT-CTGGAATAATGTAGGTAAGGGAAGTCGGCAAAATAGATCCGTAACTTCGGAATAAGGATTGGCTCTAAGGATTGGGTTAGTCGAGCTAGTATTACTCGGCTATGTGGGCAGTGTCTACGGG-CTACCT---------------------------------------GGTGGTTCTGTGGATGCTGAATGCTGGCTTGAGAGTGCCTGTGTGAGTGAGTTTCGGCT----------------------------------------------------------------------TGCTCTTTACGACTAGCAATTAACAATCAACTTAGAACTGTGACTATCTAGGGGAATCCGACTGTTTAATTAAAACATAGCATTACGATAGCCGGAAA-CGGTGTTGACGCAATGTGATTTCTGCCCAGTGCTCTGAATGTCAAAGTGCAGAAATGCAACCAAGCACGGGTAAACGGCGGAAGTAACTATGACTTTCTAAAGGTAGCCAAATGCCTCGTCATCTAATTAGTGACGCGCATGAATGGAGCAACGAGATTCCCACTGTCCCTACCTACCATCCAGCGAACCCACAGTCAAGGGAACGGGCTTGACTGGGTTAGCGGGGAAAGAAGACCCTGTTGAGCTTGACTCTAGTTTGATTTTGTGAGAAGACATGAGGGGTGTAG-AATAAGTGGGAGAT--------------GTTTCGGCATCGACAATGAAATACCACTACTTTCATCGTTTTCACACTAAATTGGTTAAAAGAGAATGACTTATTA------------------------------------------------------------------------------------ACGTAAGTTGATT----------------------------CGTAGATATAAGACA----GTTAG------------------------------------------------------------------------CACTGTTGACT----------------------------------------CTCGTCCAATGACATAATCAGATGGGGAGTTTGACTGGGGCGGTACATCCTTAAAATTGCAACGAGGATGTCCAAAGGCAAGCTCATTGTGGACAGAAACCACAAGTAGAGCAAAAGGGCAAAAGCTTGCTTGACTCTGATTTTCAGTACGAATACGAGACGTGAAATCGTGGCCTATTGATCCCTTA----CCTTCTTGAGTTTAGAATTAGGGGTGTCAGAAAAGTTACCACAGGGATAACTGGCTTGTGGCGGCCAAGCGAACATAGCGACGTCGCTTTTTGATCCTTCGATGTCGGCTCTTCCTATCATTGGGTAGCATCATGCCCAAAGTGTTGGATTGTTCACCCACCAACAGGGAACGTGAGCTGGGTTTAGACCGTCGTGAGACAGGTTAGTTTTACCCTACTAACCCATCA---GCGCTGTGACAGTAGTTCTGTTCAGTACGAGAGGAACCGCAGAACCGGATAAACGG-TGAAGCATTTGGCTGATCAGCCAGTGATGCAAAGCTGTCATCCGATGGATAATGACTGAAAGCCTC-TAAGTCAGAAGCCAGTCTG----AAGCAGTGACATACTCCGGAAA---AGCAGGGAATAAATACATTT------------------TAAATGTCATTCCGTGTGGGCGTACAGTCGT-------------------ACGTTGACAA---------------AAACCGCAATGATATTGCAGC---TCCGGGTTT-ACAGTATCGCGAAGACGACGC--GAGGTAGTCGGGGTACTGTAAGGGTGCAAGTGGACATGTAGCCACGAGACTCTGAGGTTTATCCCTCGACTGAT-TACTGTT

Henneguya_salminicola CAACCTCAGCTCAGGCAAGGCAACGCCGTGAACTAAAGCATTTCAGTAACGGCAGGAAGAGAAAATAACAATGATTCCCTTAGTAACTGCGAGTGAAGTGGGAAAAGTCCAACGTTGAAAGCTACATT--------TTTAACGAAATGTTGCATTGTAAC-GTATAGACTCGTCATCTACATGATTGCCA--ATGTTAAAGTCGCATAGAATGGCGCACCAAAGAGGGTTATAGTCCCGTAAATGGCATTGGTAACAATTGTGGCTGTATGACGATGTCAAAGAGTCGGATTGTTTGGGAATGCAGTCTGAAGTGGGTGGTAAACTCCATCCAAGACTAAATATGACTTTGAGACCGATAGCGAACAAGTACCGTGAGGGAAAGTTGAAAAGTACTCTGAAAAGAGAGTGAAAAGTGCGTGAAACCGTTAATGGGGAAGCGTACGGTAGAATCAA-AGTGTCGGATTGTGTTTTGTAATGTGTGGTGCATAACAA-------------------------------------TAATGATACGATACGCGC--------------TGGACTCATTGTTGAATGTGACTGGATGCGTATTTAGT--------TGGCAACAATGT---GTGTATCAGTAGATATTTGCATGT--------TGTTAATGAATATGAGTTGTCTGTAGAGTTG------ACAATTGGCAATGACGGATGTGTGAGAAGATGTAATGCA-TTGTGTGTGGTTGCAATGTATTGATTTTAT--AAATTGATTGCGTTGTAAAGAACATAATGT-ATGATATTTTCCA-----ATTGACTATGGTCGCAC--ACGTATTTCCAGCG-AGATATACTACTGTTCGACCCGTCTTGAAACACGGACCAAGGAGGCTAACTTGTATGCGAGTTGAGGAG--------------------TGGAGAAACTCTGAGGCGTAACGAAAGTGAAATGTGAGATTCATTGG----------------TGTTGAACTAATGACGTATCACAGACCGATTGGA---------AGAGAGACTACAATCGAGTTGAAGCATACTGGCTAGAACCCGAAAGATGGTGAACTATTCCTGAGGAGAGCGAATTCATTGGAAACAATGATGGAGGCTCGTAGCGATTCTGACGTGCAAATCGATCGTCAAACTTGGGTATAGGGGCGAAAGACTAATCGAACCATCTAGTAGCTGGTTCCCTCCGAAATATCCCTCAGGATCGCCGGAGTTGAAATATCTACACAGTTTC-ATCGGGTAGAGCGAATGATTAGAGGAATTG-GGGCTATTTATAGCCTCGACCTATTCTCAAACTGCAAACCGGTGAGAGATGTGAC-----------TGTATTACTTTTGTG-----AATATGTCACAGACC-GTCGTGAATGTGAGAGCTCCGAGTGGACCATTTTTGGTAAGCAGAACTGGTGATGCGGGATGAACCGAGAGTTGAGTTAAAGTGCCAAAGTGGGGGCCAATG-GACACCACGAAAGGAGTTGGTAAATGAAGACAATGGGGCGGTGGTCCTGAAAGTAGACATCCGCTAAGGAG-TGTGCAACAACTCACCCATCGAATTTACTGGCCCTGAAAATGGATGGCGCT-TAGCCCCCCACTGATACTCAACCGTAGAC--GCTAGAAGTG------TCGTTTACGGGTAGGAGGGTGCTATGGTTGCAGAGAAGG-CTATATGTGAATGTAGCTGGAGCGGCCATAGGTACAGATCTTGGTTTTAGTAGCAGCGAATAGAAGTG----AGAACCTTCTAGGCCGAAGTGGAGAAGGGTTCCTCGTGAACAGCAGTTGGTCGAGGGTTAGTCGGTCCTAAGCAGAAGCTCACACGCTGAGTAATAGAAAACGTA------ATGTGACATTTGTATTGTTTCGATGATATAAATAGCATT----------------------GTTTTAGTCTGCGAAAGGGAATCAGGTTAATATTCCTGAACCAGGTGTGTCGGATAGTTGATAGTGTTTAGT------TTGATGTGGTGACGCAAGTGAT-GATATCAAGTTGAATACTGTTGGCAAAGCGGTAACGCAAACAAACTCGGAGACATTGTTGACAGTTCTGGAAAGAGTTATCTTTTCTGTTTAACCAATTGTATGAGCCTGGAATAGAGTCATTCGGAGATAGGGTTATGAATTGGTAAGAGCGTCACA-TTTT-GGTGGCGTCCAGAACACTGTGAATGATCCATGAAAATCCGGGGGCGTG-----ATTGATTATCGTACCTGGCCGTACCGAGATCCGCATCAGGTCTCCAAGGTGAATAGCCTCTGGCT-CTAGAATAATGTGAGTAAGGGAAGTCGGCAAAATCGATTCGTAACTTTGGGAAAAGGATTGGCTCTAAGGATTGAGCTGATTGGACTATTTGTTCGTTGATGTGTGTTGTGTCGGCACATG------------------------------TATAGAGTTGTTTGTAACAGGATGGCTTTAGTCATGTTGTTGA--------------TGTGATATGGCAACTTCCAGCAAATAGTTTGAAATGATATAATGTATTAACCTTGTGTTGGTATATTTATA-------------TTA--TTTCCATGCAATCAGTTAAGATCAATCAACTTAGAACTGTCACTGACTAGGGGAATCCGACTGTTTAATTAAAACAAAGCATTGCGATGGCTGGAAA-CGGTGTTGACGCAATGTGAATTCTGCCCAGTGCTCTGAATGTCAAAGTGTGGAGACGCAACCAAGCGCGGGTAAACGGCGGGAGTAACTATGACTCTCTTAAGATAGCCAAATGCCTCGTCATCTAATTAGTGACGCGCATGAATGGATCAACGAGATTCCCACTGTCCCTACTTACCATCTAGCGAACAAACAGTCAAGGGAACGGGCTTGACTAAGACAGCGGGGAAAGAAGACCCTGTTGAGCTTGACTCTAGTTTGATATTGTGAGGAAACATGAGAGGTGTAGTTATAGGTGGGAGAAAGTATGCTGTTAAAGGTGTATTTTCAACAATGAAATACCACCACTCTTATCGTTTCTTCACTAATTCGGTGATGAAGAAAGTGCCCAGTTGTATGGTGTGATCA-ATATATCACTTGTGTTGTATGATTAGTTA-------------------------TGCTAATTGTATATACCAAGGATAGATTATAACCTGGCTGGTGTCATGAATT----GTACGAATTTAAAAGGGATGGCTATGTTTGGTTTGTGTGTGTGTTGATTATATTAACACATGTATGAGACGGATATGGCTGGCTCTT-GAGAGTGTGTCCTTTGATCTGTTCTG--------------ATTCCACCGTGAATCAGGTATTGACCGATGACAGTATCAAATGGGGAGTTTGACTGGGGCGGTACATTCTTTAAAATGCAACGAGAGTGTCCTAAGGTAGGCTCATCGTGGACAGAAACCACGAGTAGAGCATAAGGGCAAAAGCCTGCTTGATTTTGATTTTCAGTACTAATACAAACCACGAAAGTGTGGCCTAACGATCCTTTATGTTGACCTGAGAGTTTCAGGATAGAGGTGTCAGAAAAGTTACCACAGGGATAACTGGCTTGTGGCGGCCAAGCGTACATAGTGACGTCGCTTTTTGATCCTTCGATGTCGGCTCTTCCTATCATTGAGCTGCAGAAGACTCAAAGTGTCGGATTGTTCACCCTC--AAAGGGAACGTGAGCTGGGTTTAGACCGTCGTGAGACAGGTTAGTTTTACCCTACTGTCTTGTGAAATGCCTAGCAACAGTAATCATATTGTGTACGCGAGGACTAGTATGATCGGATAAACGG-TGACGCATTTGGTTGATAAGCCAATGATGCAAAGCTGTCATCCGCTGGATAATGACTGAACGCCTC-TAAGTCAGAAGCCACTCTG----AAGCTATGCAAAAATCCCAACCAATATTTGTGAACTATTATGTA-------------TAGTGATATACGTTGATCCATCGAGTCAATTATTTACATGTGAATAATTGATAACAGTATGTATTA--------------CTGTGAGCGATGTAAATTATG----GTTGGGTTA-GTGGTTGCGTGAAGACGACTC--GCAACAGTCATGGTGCTGTAAGGATTCGAGTGGACATTGCGCCATGAGGTCCTGAGGCTCATCCATTGACTTGG-TACCAAT

Myxobolus_squamalis CAACCTCAACTCAGGCAAGACTACGCCGTGAACTAAAGCATTTCAGTAACGGCAGGAAAAGAAAATAACCATGATTCCCTTAGTAACTGCGAGTGAAGTGGGAAGAGCCCAACGTTGAAAGCTACACC--------TTTAAACGGGTGTCGCGTTGTAAC-GTATAGATACATCATCGAGTTTTGAACCA--CATTTGAAGTTGCTTAGAATAGCACACCAGAGAGGGTTTTAGTCCCGTACATAAATGTGTGCGTCATTCAA---GTATGTTGTCTTCTAAGAGTCGGATTGTTTGGGAATACAGTCTGAAGTGGGTGGTAAACTCCATCTAAGGCTAAATATAACCTCGAGACCGATAGCGAACAAGTACCGTGAGGGAAAGTTGAAAAGCACTCTGAAAAGAACGTGAAAAGGGCGTGAAACCGTTAATGGGGAAGCGTACGGTAGAGTCGA-AGTGCAGTAACGTGGATGCGCGTATTTTGTGCAAA-------------------------CAGAGTGCGTGGCATGTAGCGATACTATACGCGC--------------CGGACTTGTTGTTGA--GTGACTGGCTTTGCAAGTGGT--------TGGTAGTAACAA---ATTATCCATGAGGATGGTTTGTGC--------TATTAGCATTTGTTAGTTAGCTATAGAGT---------CGTTTGACAATAATGTGTA-GAGCGTAGTAGTAATGTGTAT-------TGCTTTTCGGTCAAGTGCAAGTGTATGCAAACATTTGGTTGACAAGTAATAAT-AT-ATTGCTGCTA-------CAAGACAAGTCGCGT--ACGATTTTCCGGCG-AGACATGCTACTGTTCGACCCGTCTTGAAACACGGACCAAGGAGGCTAACATGTGCGCGAGTCAAGGAG--------------------TGGAGAAACTCTTAGGCGCAACGAAAGTGAAATGTGAGATCCCACGG----------------C-TAACGCTGGGGGCGCATCACAGACCAGTTGGA---------AAATTGACTACGATTGAGTTGCAGCGTACATGCTAGAACCCGAAAGATGGTGACCTATTCCTGAGCAGAGCGAATTCATTGGAAACAATGATGGAGGCTCGTAGCGATTCTGACGTGCAAATCGATCGTCAAACTTGGGTATAGGGGCGAAAGACTAATCGAACCATCTAGTAGCTGGTTCCCTCCGAAATATCCCTCAGGATCGCCGAGATTCGTA---GTACACAGTTTC-ATCGGGTAGAGCGAATGATTAGAGGAATTG-GGGATATTTATGTCCTCGACCTATTCTCAAACTGCAAACCGGTGAGTGAATGGGA-----------ACGGTTGCTTGATTG-----AACCGTATGC--CTGTATCGTGAATGTGAGAGTCTCGAGTGGGCCATTTTTGGTAAGCAGAACTGGCGATGCGGGATGAACCGAGAGTTGGGTTAAGGTGCCAAAGTGGGGGCAAATG-GACACCACAAAAGGAGTTGGTAAATGAAGACAGTAGGACTGTGGCTCTGAAAGTAAGCATCGGCTAAGGAG-TGTGCAACAACTCACCTACCGAATTTACCGGCCCTGAAAATGGATGGCGCT-TAGCCCCCCACCGATACCCGACCGTAGAC--GCGAGAAAAGTA----TCGTCTATGGGTAGGAGGGTGCTATGGTTGCTGCAAAGG-CTAGTCGTGAGACTTGCTGGAGCGTCCATAGGTACAGATCTTGGTTTTAGTAGCAGCGAATAGAAGTG----AGAACCTTCTAGGCCGAAGTGGAGAAGGGTTCCTCGTGAACAGCAGTTGGACGAGGGTTAGTCGGTCCTAAGCAGCAGCTCACACGCTGAGTAAAAGGGTACGTT------TTGTA---------ATTGTGTGCTTGCATACATTACTTC----------------------GTACCAGTCTGCGAAAGGGAATCAGGTTAATATTCCTGAACCTG--GCGTCGGATAGCTAATATTGTTGTGGCAGTTGTTCGTATTGGTTTACCAATTGC-GTCAATTGTTGTGAAAGTGTTGGTGAAGCGGTAACGCAAACGAACTCGGAGACGTTGTTGGTAGTTCCAGGAAGAGTTATCTTTTCTGTTTAACCGATTGGACGAGCCTGGAATAGAGTCGTTCAGAGATAGGCTCGACAATGGGGAAGAGCGTCGCA-TTTT-GGTGGCGTCTGGAGTACTACCAACGATCCATGAAAATCCGAGGAAGTG-----ATTGATTTTCGCGCCTGGCCGTACCGAGATCCGCATCAGGTCTCCAAGGTGAATAGCCTCTGGCT-CTAGAATAATGTGGGTAAGGGAAGTCGGCAAAATAGATTCGTAACTTCGGGAAAAGGATTGGCTCTAAGGATTGGGCTGTTGAAGCTATATACTTGTTGGTGCGCGACGTGTAAATGGGTGTCTGTCG-----------------------TAGAGTTTGGTTTATTCCGGGCTTGAAAACAGT-ATCTGTATA--------------TATGACGTGGTGTCTTCCAGCGTATAGTTTGTAGAGTGTAC--GTGTTGGACTCGTCCAGCATACTACACT-------------TTA-----CCTCGTCAATGGCTAAGATCAATCAACTTAGAACTGTCACTGACTAGGGGAATCCGACTGTTTAATTAAAACAAAGCATTGCGATGGCCGGAAA-CGGTGTTGACGCAATGTGAATTCTGCCCAGTGCTCTGAATGTCAAAGTGTGGAGACACAACCAAGCGCGGGTAAACGGCGGGAGTAACTATGACTCTCTTAAGGTAGCCAAATGCCTCGTCATCTAATTAGTGACGCGCATGAATGGATCAACGAGATTCCCACTGTCCCTACCTACCATCTAGCGAACAAACAGTCAAGGGAACGGGCTTGACTGAGACAGCGGGGAAAGAAGACCCTGTTGAGCTTGACTCTAGTTTGATACGGTGAAAAGACATGAGGGGTGTAG-AATAGGTGGGAGATGTTGTACTAGTAATAGTGCTTCATCAACGGTGAAATACCACTACCCTCATCGTTTCTTTACTCAATCGGTGATAAGGGAAGTGCCCGGTTTA-TGAATCATTCATTAATGCTACTT------CATGGCTATTGT----TGGCACTTGTGTAACAGCAAATGTTGACAGTGGTTGAGAAGAGCAGGATGTATTCAGGCTGGTGTCACGACTT----GTTAGAACTAAGATG----GGCTGCCTTGTGTTTTGCTGTGACTTTGTGGCAACACTGAGAAATAGTAAGGTACTTGGCTAGCTCTT-GAGAGTGTGTCCATCGACCCATACCGTAGTGGTGA-----AAGCTACTGCGGGTT----CGTGACCGGTGACAATATCAGATGGGGAGTTTGACTGGGGCGGTACATTCTTTAAAATGCAACGAGAGTGTCCTAAGGTGGGCTCACCGTGGACAGAAACCACGGGTAGAGCATAAGGGCAAAAGCCCGCTTGATTTTGATTTTCAGTACGAATACAAACCACGAAAGTGTGGCCTAACGATCCTTTAAGTTGACCTGAGAGTTTCAGGATAGAGGTGTCAGAAAAGTTACCACAGGGATAACTGGCTTGTGGCGGCCAAGCGTTCATAGCGACGTCGCTTTTTGATCCTTCGATGTCGGCTCTTCCTATCATTGAGAAGCAGTATTCTCAAAGTGTCGGATTGTTCACCCGTT-CAAGGGAACGTGAGCTGGGTTTAGACCGTCGTGAGACAGGTTAGTTTTACCCTACTGTCTCGAGAAATGCGTAGCTACAGTAATCCTATTTAGTACGCGAGGACCAGTAGGTTCGGATGAACGG-TGACGCGTTTGGTTGATAAGCCAGTGACGCAAAGCTGCCATCCGTTGGATAATGACTGAACGCCTC-TAAGTCAGAAGCCACTCTG----GAGCTATGCAAAAGTCCCAATTGTAATTTGAGAAAGAATACGCGTCAGTAGTT----TACTACTGCGTGTAGATCCATCGAAGCTATCAATCAG-AATGGTTGGTGGTTCGCAATGTAAA-----------------TTGCAAACGATGTAGATTGTG----ATTGGGTTG-ATGGTTGCGTGAAGACGACTC--GCAACAGTCGCGGTGCTGTAAGGATTCGAGTGGATTTTTCGCCATAAGGTCCTGAGGCTCATCCGTCGACTTGC-TACCAAA

Thelohanellus_kitauei CAACCTCAACTCAGGCAAGGAAACGCCGTGAACTAAAGCATTTCAGTAACGGCAGGAAAAGAAAATAACCATGATTCCCACAGTAACGGCGAGTGAAGTGGGAAGAGTCCAACGTTGAAAGCGGCATC--------TGTAACAAGGTGTGGCATTGTAAC-GTATAGACTTGTCATCGAGGCGTCCGACC--GGACTAAAGTCGTTAAGAATGGCGCACCATAGAGGGTTGTAGTCCCGTACATGGTTCGGTATCGGATGTCG--TGAATGGCAAAGTCAAAGAGTCGGATTGTTTGGGAATGCAGTCCAAAGTGGGTGGTAAACTCCATCTAAGGCTAAATATTACTCCGAGACCGATAGCAAACAAGTACCGTGAGGGAAAGGTGAAAAGAATTCTGAGAAGAGAGTGAGAAGAACGTGAAACCGTTAGTGGGGAAGCGGACAATGTAATCGT-AGTTGTTTGGTGTTTGTGTTTTTCAA----------------------------------CTTGTTGTTAAATTCTTAACGCCGAATAACGCGC--------------CAGGCTCTATGCTAA-TGCAGCTGGCAGTGTATTGGGT--------GGTTCGACAAAG---AGTGTTTCTTGAGAAATGCTCTGT--------CGGCTGACAGTACAAACTGT-TGTAGAGT--------TGGCGCGGCATGGATGTGTG----TGCTTGTGCGTTGAAGGTGCGTTGGCGTGTAGTCGGTGCGATCCCACAAAGTGGGACACATTGGTGTAGCGTTGGCGT-AGTTTTGACGTCT-------CGACGAATGTCGCAC--ACAAACTCTTGGCG-AGATATGACATTGTCCGACCCGTCTTGAAACACGGACCAAGGAGGCTAACATATGCGCGAGTCGAGGAG--------------------TGGAGAAACTCTATGGCGTAACGAAAGTGAAATGTGAGATTGTGCGA----------------CGTCGAGTCGTGCACGCATCACAGACCAGTCGGA---------AGTTTGACTACGATTGAGTTGGAGCGTATATGCTAGAACCCGATAGATGGTGACCTATTCCTGAGCAGAGCGAATTCATTGGAAACAATGATGGAGGCTCGTAGCGGTTCTGACGTGCAAATCGATCGTCAAACTTGGGTATAGGGGCGAAAGACTTATCGAACCATCTAGTAGCTGGTTCCCTCCGAAATATCCCTCAGGATCGCCGAAGCTCG-------CAGCAGTTTC-ATCGGGTAGAGCGAATGATTAGAGGCATCG-GGGCTATTTTTAGCTTCAACCTATTCTCAAACTGCAAACCGGTGACATGTGGTCA-----------GCGATTTCTTGATTG-----AATCGTTGATGCCGTAAGGCTGAATGCATGAGTTTCGAGTGGGCCGTTTTTGGTAAGCAGAACTGGCGATGCGGGATGAACCGAGAGTCGGGTTAAAGTGCCTAAGTCGCGGCCAACG-GATACCATAAAAGGAGTTGGTAAATTATGACAGTGAGGCTGTGGTCCTATAAGTAGATATCAGCTAAGAAG-TGTGCAACAACTTACTTACCGAATTTACTGGCCCTGAAAATGGATGGCGCT-TAGCCGCGCACTGATACCCGACCGTAGAT--GCGAGAAATGTG----TCATCTATGGGTAGGAGGGTGCTATGGTTGCGTAAAAGC-GTCCACGTGAGTGGGTGTGGAGCAGCCATAGGTACAGATCTTGGTTTTAGTAGCAGCGTATAGAAGTG----AGAACCTTCTAGGCCGAAGTGGAGAAGGGTTCCTCGTGAACAGCAGTTGGACGAGGGTTAGTCGGTCCTAAGCAGAAGCTCACACGCTGAGTAATAGGAGACGTT------TTGTA--------TGTAATGTGCTCGCACATAATGCTTC----------------------GTCTCAGTCTGCGAAAGGGAATCAGGTTAATATTCCTGAACCAG--GCGTCGGATAACCAGTATTGTCTGGC--GATACCGGGAGGCGTGAGTCGCTT----GGAATTGCCGTGGTAATGTTGGTAAAGCGGTAACGCAAACGAACTCGGAGACGTTGTTGGTAGTTCTGGAAAGAGTTGTCTTTTCTAGTTAACTGGTTGAACGAGCCTGGAATGGAGTCATTCAGAGATAGGCTTGACAGCCGGGAAGAGCGTCGCA-TCTG-AGTGGCGTCCGGAATACTACCAATGATCCTTGAAAATCCGAGGGAGTG-----AATGATTGTCGTGCCTGGCCGTACCGAGATCCGCATCAGGTCTCCAAGGTGAAAAGCCTCTGGCT-CTAGAATAATGTGGGTAAGGGAAGTCGGCAAAATAGATTCGTAACTTCGGGATAAGGATTGGCTCTAAGGATTGGACTATTCGGATTGGCGTCTCGTAAAAGAGACGATCGTTGACGCTGT-TTGTCG-----------------------TGGCCAGATGGGT-TACCGTGTGGTCGCGTATTCAGATGTTGG--------------CGTATTGTTACTGTTACCAGGTGTCAATTTACAGTGGCGTCGGCAGGCAATTTAATTGTTGTCAGCCGATGCC-----------CTG-----TCTCGCGAGTAGTTTAAATCAATCAACTTAGAACTGTCACTAACTAGGGGAATCCGACTGTTTAATTAAAACAAAGCATTGCGATGGCTGGAAA-CGGTGTTGACGCAATGTGAATTCTGCCCAGTGCTCTGAATGTCAAAGTGTGGAGACACAACCAAGCGCGGGTAAACGGCGGGAGTAACTATGACTCTCTTAAGATAGCCAAATGCCTCGTCATCTAATTAGTGACGCGCATGAATGGATCAACGAGATTCCCACTGTCCCTACCTACCATCTAGCGAACAAACAGTCAAGGGAACGGGCTTGACTGAGACAGCGGGGAAAGAAGACCCTGTTGAGCTTGACTCTAGTTTGATATGGTGAGGAGACATGAGAGGCGTAG-AATAGGTGGGAGAGATT----TGGGGCAACCCAGGTTTCGCCGGTGAAATACCACCACTCTTATCGTTTCTTTACTCAATCGGTGATGTAGGAGGCGCCCAGTTGC-TGTGTTGGTCA-TTAAGCTACTT------CATGGCTATCGATGGCAGACTCTGGGTAAACCAGGTTTGTCGTCTTTAGTTGAGGAGAGCAGACT-CGACCGGACTGGATACGCCGCTT----GTAAGATTTAAGATG----GGCTGTCTGCTATG---GTTGTGCCAACTGGGCAACCATTTGGTACGGC-CGTGGTTGACTGGCTTTTCGAGAGTGTGTCCATCGACCTAGCCGTGGTTGGCGAGCAATCGTCAATTGCAGGT-----TACGACCGATGACAGTATCAAATGGGGAGTTTGACTGGGGCGGTACATTCTTTAAAATGCAACGAGAATGTCCTAAGGTGGGCTCACCGTGGACAGAAACCACGGGTAGAGTATAAGGGCAAAAGCCTGCTTGATTTTGATTTTCAGTACGAATACAAACCACGAAAGTGTGGCCTAACGATCCTTTATGTTTGCCTGAGAGTTTCAGGATAGAGGTGTCAGAAAAGTTACCACAGGGATAACTGGCTTGTGGCGGCCAAGCGCCCATAGCGACGTCGCTTTTTGATCCTTCGATGTCGGCTCTTCCTATCATTGAGCAGCAGCGTACTCAAAGTGTCGGATTGTTCACCCGCT-TCAGGGAACGTGAGCTGGGTTTAGACCGTCGTGAGACAGGTTAGTTTTACCCTACTGTCTCTTGGAATGCGTAGCAACAGTAATCCTATATAGTACGTAAGGACCTGTAGGTTCGGATGAACGG-TGGCGCGTTTGGTTGATCAGCCAATGATGCAAAGCTGTCATCCGTAGGATAATGACTGAACGCCTC-TAAGTCAGAAGCCAGTCTG----GAGCTATGCAAAAATCCCGACTTTTATTTGTGAAACAATACACG--AGCAAT-------------CGTGTGAATCCATCGTGGTGGTCGACATG--ATTGTAGGCCACTTGGAGAGTGTATCT--------------CTCTGAGCGATGTAAATTGAA----GTTGGGTCATGTGGTTGCGAGAAGACGACTC--GCAACAGTCGCGGTGCTGTAAGGATTCAAGTGGACTTTGCGCCATGAGATCTTGAGGCTCATCCGTCGACTTTA-AACCAAA

Myxobolus_shantungensis CAACCTCAACTCAGGCAAGACTACGCCGTGAACTAAAGCATTTCAGTAACGGCAGGAAAAGAAAATAACCATGATTCCTTCAGTAACGGCGAGTGAAGTGGGATCAGTCCAACGTTGAAAGCAGCACC--------CATAACAAGGTGTGGCATTGTAAC-GTATAGACTTGCCATCGAGACATTTGGCT--GGACCTAAGTCGTTAAGAGTGACGCACCACAGAGGGTTGTAGTCCCGTGTATGGTTCGGTTTCAGATGTCA--AGCATGGCAAAGTCAAAGAGTCGGATTGTTTGGGAATGCAGTCCTAAGCAGGTGGTAAACTCCATCTAAGACTAAATATAACTCCGAGACCGATAGCAAACAAGTACCGTGAGGGAAAGGTGAAAAGAACTCTGAGAAGAGATTGAAAAGAACGTGAAACCGTTAATGGGGAAGCATACAATGTAATCGT-AGGGTTGTAATATTTGTGTCGAGCGGTGGTCGTC--------------------------TCCACTGATCGGTGCTTAATTTTACGACGCGCGC--------------TAGGCTCTATGCTAA-GGTCATTGTCAGTGTGTTTAGT--------GGTTCATCAACG---GCCCTTCCTTGAGGAATGGTTAGG--------TGGCTGTGGGCACGTGCTGT-TGTAGAAT---------GACGCGGCATAGATGTATG----TGCTTGTGTGCCCTA-TCGTATGGACGTAAAGTTGATGTTGT-TCACTATGTGGGTAGCGTGGATGTAGCGTTTTTGC--GGTATGGTACCA-------CGATGAAGGTCGCAC--ATAAACTCTTAGCG-AGATATGACATTGTCTGACCCGTCTTGAAACACGGACCAAGGAGGCTAACATGTACGCAAGTCGAGGAG--------------------TGGAGAAACTCTATGGCGCAACGAAAGTAAAATGTGAGATCACGCAA----------------TGTGGAGTTGTGTGCGCATCACAGACCAGTCGGA---------AAATTGACTACGATTGAGTTGGAGCGTACATGCTAGGACCCGATAGATGGTGACCTATTCCTGAGTAGAGCGAATTCATTGGAAACAATGATGGAGGCTCGTAGCGGTTCTGACGTGCAAATCGATCGTCAAACTTGGGTATAGGGGCGAAAGACTAATCGAACCATCTAGTAGCTGGTTCCCTCCGAAATATCCCTCAGGATCGCCGAGACTCG------TTAGCAGTTTC-ATCGGGTAGAGCTAATGATTAGAGGACTCG-GGGCTATTTATAGCCTCGACCTATTCTCAAACTGCAAACCGGTGACCTTGTCA-------------GTGATTACTTGATTG-----AATCGCTGATACCGTAAGGGCGAATGTA-GAGTCTCGAGTGGGCCGTTTTTGGTAAGCAGAACTGGCGATGCGGGATGAACCGAGAGTCGAGTTAAAGTGCCTAAATGGCGGCAAACG-GACACCATAAAAGGAGTTGGTAAATTATGACAGTGAGGCTGTGGTCCTGTAAGTAGATATCAGCTAAGAAG-TGTGCAACAACTTACTTACCGAATTTACTGGCCCTGAAAATGGATGGCGCTGTAGCCGTCTACTGATACTCGACCGTAGAC--GCGAGAAAAGTT----ACGTCTATGGGTAGGAGGGTGCTATGGTTGCGTAGAAGC-GTACTTGTGAGAGTGCGTGGAGCAGCCATAGGTACAGATCTTGGTTTTAGTAGCAGCGTATAGAAGTG----AGAACCTTCTAGGCCGAAGTGGAGAAGGGTTCCTCGTGAACAGCAGTTGGACGAGGGTTAGTCGGTCCTAAGCAGAAGCTTACATGCTGAGCAATAGGGGACGTT------TTGTA--------GCTAGTGTGTTTACACACGATACATC----------------------GTCTCAGTCTGCGAAAGGGAATCAGGTTAATATTCCTGAACCAG--GCGTCGGATAACCAGTGTCGTACGAC--GATGTTG--------GAGCAATTC----AGCGTTGTT-GAGTGGCACTGGTGAAGCGGTAACGCAAATGAACTCGGAAACGTTGTTGGTAGTTCTGGGAAGAGTTGTCTTTTCTGTTTAACCAGTTGTTTGAGCCTGGAAAAGAGTCATTCAGAGATAGGGTTAGCAACTGGGAAGAGCGTCGCA-TTTT-TGTGGCGTCTGGAATACTGCCAATGATCCTTGAAAATCCGAGGGAGTG-----ATTGATTGTCGTGCCTGTCCGTACCGAGATCCGCATCAGGTCTCCTAGGTGAAAAGCCTCTGGCT-CTAGAATAATGTGGGTAAGGGAAGTCGGCAAAATAGATTCGTAACTTCGGGATAAGGATTGGCTCTAAGGGTTGGACTATTCGGGCTGTTGTCTCGTGCCGGTAGCGATCGTTGACGCTGA-AGATT------------------------TGGCTTGGCGGGC-AACCGTTAGGTCGGTCTTTCTGTTGTTGA--------------TGTGTTGCAACTGGTACCAGATGTCAGTTTACAGTTGCTGA--TGGGTGAATTTAT--TTGCCTGTCGGTG-------------CTG-----TCTCGCGAGTAGCCTAAATCAATCAACTTAGAACTGTCACTAACTAGGGGAATCCGACTGTTTAATTAAAACAAAGCATTGCGATGGCTGGAAA-CGGTGTTGACGCAATGTGAATTCTGCCCAGTGCTCTGAATGTCAAAGTGTGGAGACACAACCAAGCGCGGGTAAACGGCGGGAGTAACTATGACTCTCTTAAGATAGCCAAATGCCTCGTCATCTAATTAGTGACGCGCATGAATGGATCAACGAGATTCCCACTGTCCCTACCTACCATCTAGCGAACAAACAGTCAAGGGAACGGGCTTGACTGAGACAGCGGGGAAAGAAGACCCTGTTGAGCTTGACTCTAGTTTGATATGGTGAGGAGACATGAGAGGTGTAG-CATAGGTGGGAGAACGG---CAGCTTCGGCTGTTTTTTCGACGTTGAAATACCACCACTCTTATCGTTTCTTCACTCAATCGGTGATGAAGGAAGCGCCCAGTATC-TGTTCAAGTCG-TATAGCTACTT------CATGGCTGTCGACTATAGGCAGGAGTAATATCTTGTTTGTGGAGGGCAGTTGAGGAGAGCTGACT--TGACGGGCTGGTTACGCCAATT----GTAAGAATTAAGATG----GGCTGTCCTCAGTT-----GGCTGCATGTGGGCAACCCCGTGTGTAGTT-GGCTGTTGACTGGCTTTTCGAGAGTGTGTCCATCGACCTAGCCGTGGTTGTCGAGCGATCGGCAATTGCAGGT-----AGAGACCGATGACAGTATCAAATGGGGAGTTTGACTGGGGCGGTACATTCTTTAAAATGCAACGAGAATGTCCTAAGGTGGGCTCACCGTGGACAGAAACCACGGGTAGAGTATAAGGGCAAAAGCCTGCTTGATTTTGATTTTCAGTACGAATACAAACCACGAAAGTGTGGCCTAACGATCCTTTATGTTTGCCTGAGAGTTTCAGGATAGAGGTGTCAGAAAAGTTACCACAGGGATAACTGGCTTGTGGCGGCCAAGCGAAGACAGCGACGTCGCTTTTTGATCCTTCGATGTCGGCTCTTCCTATCATTGAGCAGCAGCATACTCAAAGTGTCGGATTGTTCACCCGTT-TTAGGGAACGTGAGCTGGGTTTAGACCGTCGTGAGACAGGTTAGTTTTACCCTACTGTCTCTTGAAATGCGTAGCGACAGTAATCCTATTTAGTACGTGAGGACCAGTAGGTTCGGACGAACGG-TGGCGCGTTTGGTTGATAAGCCAGTGATGCAAAGCTGTCGTCCGTAGGATAATGACTGAACGCCTC-TAAGTCAGAAGCCAGTCTG----GAGCTATGCAAAAATCCCGACTTATATTTGTGAAAGTATACATG--AGCAAT-------------CATGTGAATCCATCGTGGCGGTTGTCTGA--ATGGTCAACTGTTAGGGGTGTGTAACA--------------CCTCGAGCGATGTAAATTGAA----GTTGGGTCAACTGGTTGCGAGAAGACGACTC--GCAACAGTCGCGGTGCTGTAAGGATTCGAGTGGACTTTGCGCCATGAGATCTTGAGGCTCATCCGTCGACTTGT-AACCAAA

Myxobolus_wulii CAACCTCAAATTAGGCAAGAAAACGCCGTGAACTAAAGCATTTCAGTAACGGCAGGAAAAGAAAATAACAATGATTCCCTCAGTAACTGCGAGTGAAGTGGGAAAAGTCCAACGTTGAAAGCAGCATC--------CGTAAAAAGGTGTGGCATTGTAAC-GTATAGACCGATCATCGAGGTGTTTGGCC--GTACTAAAGTCGTTAAGAATGGCGCACCATAGAGGGTTATAGTCCCGTATGTGGTATGGTTTG-AACATTG--TGAATGGTCGAGTCATAGAGTCGGGCTGTTTGGGAATGCAGTCTAAAGCGGGTGGTAAATTCCATCAAAGACTAAATATAGCTTCGAGACCGATAGCGAACAAGTACCGTGAGGGAAAGTTGAAAAGCACTCTGAAAAGAAAGTGAAAAGTACGTGAAACCGTCAATAGGGAAGCAGACAGTGTAATCGA-AGCGTCCGTTTGTTTGTGGTAAG-------------------------------------TTTACAACTTATCAATTAGCAGATGGATGCGCGC--------------TAGACTAATTGAAGA--GCTACTGTCATGGTTGATAGT--------TGTAAAGATGAA---TGTTTCCATGAGGATTTGTTCGCT--------TTGCAGTATCAACTAATTGT-CTTAGAGT----------AGTTTTTTAATTTTTATGTGTGAGTGATTATTTTGTTTGCGTATACGGGTGCTATAGTTTGATTGTAATACAGTTAATTGTAGTATTCGTGTGCGGAAAT-AGGGTGGTTGCCA-------AAACGAAAGTTGCAC--ATGTA-TTCTAGCG-AGATATGACACTTTCTGACCCGTCTTGAAACACGGACCAAGGAGGATAACTTGTACGCGAGTCGAGAAG--------------------TGGAGAAACTTTCTGGCGAAACGAAAGTGAAATGTGAGATCTTGTG-----------------TGTAAAAGCATGAGCGTATCACAGGCCAGTTGAA---------GGGTTGCCTTTGATTGAGTTGGAGCGTACAGGCTATGACCCGAAAGGTGGTGACCTATTCCTGGGGAGAGCGAATTCATTGGAAACAATGATGGAGGCTCGTAGCGATTCTGACGTGCAAATCGATCGTCAAACCTGGGTATAGGGGCGAAAGACTAATCGAACCACCTAGTAGCTGGTTCCCTCCGAAATATCCCTCAGGATCGCCGAAACTCA-------AGGCAGTTTC-ATCGGGTAGAGCTAATGATTAGAGGATTTGTGGGCTATTTATAGCCTCAACCTATTCTCAAACTGCAAACCGGTGATTAGATTG-------------ACGTTTTCTTAGTTG-----AATTGTCAGT-------GACTGAATGTGTGAGTTTCGAGTGGGCCATTTTTGGTAAGCAGAACTGGCGATGCGGGATGAACCGAGAGTCGGGTTAAAGTGCCTAAATGGAGGCAGATG-GATACCACAAAAGGAGTTGATGAATTATGACAGTAAGGCTGTGGTCCTGAAAGTAGACATCAGCTAAGAAG-TGTGCAACAACTTACTTACCGAATTCATCGGCCCTGAAAATGGATGGCGCT-TAGCCTTCTACTGATACCCGACCGTGGAC--GCGAGAAATGTA----TCGTTCATGGGTAGGAGGGTGCTATGGTTGCGTAGAAGC-TTGTACGTGAGTATAAGTGGAGCTGCCATAGGTACAGATCTTGGTTTTAGTAGCAGCGTATAGAAGTG----AGAACCTTCTAGACCGAAGTGGAGAAGGGTTCCTCGTGAACAGCAGTTGGACGAGGGTTAGTCGGTCCTAAGCAGCAGCTCACACGCTGAGTCATAGGAGACGTT------TTGTA--------GATAGCATGCTTGCGTGTGATACATC----------------------GTCTTAGTCTGCGAAAGGGAATCAGGTTAATATTCCTGAACCAA--GTGCCGGATAGTTGGTGCTGTTTCTGGTAGTGTCCGAGCGGGACTTGTGCTTGCGAGGTGCTATCGGGGCAGTATTAGCAAAGCGGTAACGCAAACGAACTTGGAGACGTTGTTGATAGATCTGGAAAGAGTTATCTTTTCTGTTTAACCGATTGAGTGAGCCTGGAATAGAGTCATTCGGAGATAGGCTTAACAATTGGGAAGAGCGTCGCA-TTTT-AGTGGCGTCCGGAATTCTGTCAATGATCCGTGAAAATCCAAGGGAGTG-----GTTAATTTTCGCACTTGGCCGTACCGAGATCCGCATCAGGTCTCCTAGGTGAATAGCCTCTGGTT-CTAGAATAATGTGGGTAAGGGAAGTCGGCAAAATAGATTCGTAACTTCGGGATAAGGATTGGCTCTAAGGATTGGACTGTTTGGACCAGTGGATGCTGAGTGCGGTTTTCGTCTACGGTTG-GTGAGA-----------------------TGGGGGGTAGTTT-CGGCTATTCTGCGTCTTGTCAACTGTTAA--------------CGAGCTACTGCGTTCTTCATCTGCTGGTTTGTATCGATGCA--TTGTTAGCCGTGTGTTGGCAGTGTGTTG-------------GTG-----CCTCGCAGGCAGTTGAAATCAATCAACTTAGAACTGTCACTAACTAGGGGAATCCGACTGTTTAATTAAAACAAAGCATTGCGATGGCTGGAAA-CGGTGTTGACGCAATGTGAATTCTGCCCAGTGCTCTGAATGTCAAAGTGTGGAGACACAATCAAGCGCGGGTAAACGGCGGGAGTAACTATGACTCTCTTAAGATAGCCAAATGCCTCGTCATCTAATTAGTGACGCGCATGAATGGATCAACGAGATTCCCACTGTCCCTACCTACCATCTAGCGAACAAACAGTCAAGGGAACGGGCTTGACTGAGACAGCGGGGAAAGAAGACCCTGTTGAGCTTGACTCTAGTTTGATATGGTGAGGAGACATGAGAGGTGTAG-TATAGGTGGGAGATGCG----GGTGTTCGCATTCGTGTCGACAGTGAAATACCACTACTTTTATCGTTTCTTCACTAAATCGGTGATGAAGGAAGCGCCCGCTTGC-TGGTGTAGTCG-TAATGCTGCTT------TTCGGCTGGCGG----AGGCTGTGGGTTCACTTG--CAGTTTTTGTCAGTTGAGGAGAGCTGATTATTACCGAAGTGGATACGCGAATTATCCGTAAGCATTAAGATG----GGCTGCTTCTGATG-----GGCAAGTGTTGGTGCAAATTGATGCTTGTT-TGTCAGTTGCTGGCTTTTCGAGAGTGTGTCCATCGACCTTTCCGTGGGCAGGCAGCAATGTTTGTCCATGGGTTCG--AATGACCGATGACAGTATCAAATGGGGAGTTTGACTGGGGCGGTACATTCTTTAAAATGCAACGAGAATGTCCTAAGGTGGGCTCACCGTGGACAGAAACCACGGGTAGAGTATAAGGGCAAAAGCCTGCTTGATTTTGATTTTCAGTACGAATACAAACCACGAAAGTGTGGCCTAATGATCCTTTATATTGGCCTGAGAATTTCAGGATAGAGGTGTCAGAAAAGTTACCACAGGGATAACTGGCTTGTGGCGGCCAAGCGAACACAGCGACGTCGCTTTTTGATCCTTCGATGTCGGCTCTTCCTATCATTGAGCAGCAGAGTACTCAAAGTGTCGGATTGTTCACCCGCT-TCAGGGAACGTGAGCTGGGTTTAGACCGTCGTGAGACAGGTTAGTTTTACCCTACTGTCTCTTGTAATGCGTAGCAACAGTAATCCTATATAGTACGTGAGGACCTGTAGGTTCGGATGAACGG-TGACGCGTTTGGTTGATAAGCCAATGATGCAAAGCTGTCATCCGTAGGATAATGACTGAACGCCTC-TAAGTCAGAAGCCAGTCTG----GAGCTATGCAAAAA-CCTGACTTGAATTAGTGAAAAAATACACG-------------------CAAGTGTGATTCCATCGTGGTGGCTGATTTA-TATAATTGGTCATTAGAAGTGTGTAACA--------------TTTCGAGCGATGTAGATTGAA----GTTGGGTAATATGGTTGCGTGAAGACGACTC--GCAACAGTCGCGGTGCTGTAAGGATTCGAGTGGACTTGTTGCCATAAGATCTTGAGGCTCATCCGTCGACTTGT-AACCAAA

Myxobolus_honghuensis CAACCTCAAATTAGGCAAGACAACGCCGTGAACTAAAGCATTTCAGTAACGGCAGGAAAAGAAAATAACAATGATTCCCTCAGTAACTGCGAGTGAAGTGGGAAAAGTCCAATGTTGAAAGCAGCATC--------TGTAAAAGGGTGTGGCATTGTAAC-ATATAGACCAATCATCGAGGTGTTTAGTT--GTGCTAAAGTCGTTAAGAATGGCGCACCATAGAGGGTGATAGTCCCGTATATGGTACAGCATA-AGCATTG--TGAATGATTGTGTCATAGAGTCGGGCTGTTTGGGAATGCAGTCTAAAGTGGGTGGTAAACTCTACCAAAGACTAAATATAGCTTCGAGACCGATAGCGAACAAGTACCGTGAGGGAAAGTTGAAAAGCACTCTGAAAAGAAAGTGAAAAGTACGTGAAACCGTCAATGGGGAAGCGGACAGTGTAATCGA-AGCGTCTAGCTGTTTGCGATGAGT------------------------------------TTTATTACTTGTCGATTAGCAGAGAGACGCGCGC--------------TAGACTGTTTAGCGG--TTTACTGGTAGCGTGACGAGT--------TATAAAGATGAA---TGTTTCCATGAGGATTTGTTCGCT--------TTGTAGTCGTTGTGGTCTAT-TTTAGAGT---------AAATTGTTAAACATGTATGTGTGAGTAATGGTTATGCTGGTATGTGCGGGTATTGTAGTTTGGTTGTAATACAGTTGAATGCAGTGTTCGTGCATGTTAGT-ATGGTTGTTGCCA-------AAACGATAGTTGCAC--ATGTA-TTCTAGCG-AGATATGACACTTTCCGACCCGTCTTGAAACACGGACCAAGGAGGCTAACTTGTGCGCGAGTCGAGAAG--------------------TGGAGAAACTTTATGGCGAAACGAAAGTGAAATGTGAGATCTTGTG-----------------TTTAACAGCATAAGCGTATCACAGGCCAGTTGAA---------GGGTTGCCTTCGATTGAGTTGGAGCGTACAGGCTAGGACCCGAAAGGTGGTGACCTATTCCTGAGGAGAGCGAATTCATTGGAAACAATGATGGAGGCTCGTAGCGATTCTGACGTGCAAATCGATCGTCAAACTTGGGTATAGGGGCGAAAGACTAATCGAACCACCTAGTAGCTGGTTCCCTCCGAAATATCCCTCAGGATCGCCGAAACTCT-------AGGCAGTTTC-ATCGGGTAGAGCTAATGATTAGAGGATTCGTGGGCTATTTATAGCCTCGACCTATTCTCAAACTGCAAACCGGTGATTACATTG-------------ACGTTTTCTTTGTTG-----AATTGTTAAT-------GGCTGAATGTGTGAGTTTCGAGTGGGCCATTTTTGGTAAGCAGAACTGGCGATGCGGGATGAACCGAGAGTCGGGTTAAAGTGCCTAAATGGAGGCAGACG-GATACCACAAAAGGAGTTGATGAATTATGACAGTAAGGCTGTGGTCCTGAAAGTAGACATCAGCTAAGAAG-TGTGCAACAACTTACTTACCGAATTCATCGGCCCTGAAAATGGATGGCGCT-TAGCCTTCTACTGATACCCGACCGTGGAC--GCGAGAAATGAT----TCGTTCATGGGTAGGAGGGTGTTATGGTTGCGAAGAAGC-TTGCATGTGAGTGTAAGTGGAGCAGCTATAAGTACAGATCTTGGTTTTAGTAGCAGCGTATAGAAGTG----AGAACCTTCTAGACCGAAGTGGAGAAGGGTTCCTCGTGAACAGCAGTTGGACGAGGGTTAGTCGGTCCTAAGCAGCAGCCTACAAGCTGAACCATAGAAGACGTT------TTGTA--------GTTAGTATGCTTGCATGCGATACATC----------------------GTCTTAGTCTGCGAAAGGGAATCAGGTTAATATTCCTGAACCAA--GCGTCGGATAGTTGGTATTGTTTCTGGTAGTATCCGAGCGGGACTCGTGCTTGCGAGGTGCTATCAGGGCAGTATCAGCAAAGCGGTAACGCAAACGAACTCGGAGATGTTGTTGATAGTTCTGGAAAGAGTTATCTTTTCTGTTTAACCGATTGAGCGAGCCTGGAGTAGAGTCATTCGGAGATAGGCTTGACAATTGGGAAGAGCGCCGCA-TTTT-AGTGACGTCCGGAATACTGTCAATGATCCGTGAAAATCCGAGGGAGTG-----GTTAATTTTCGCGCTTGGCCGTACCGAGATCCGCATCAGGTCTCCAAGGTGAATAGCCTCTGGTT-CTAGAATAATGTGGGTAAGGGAAGTCGGCAAAATAGATTCGTAACTTCGGGAAAAGGATTGGCTCTAAGGATTGGACTGTTTGGACCAGTAGATACTGATTGTGGTTCTTGTATACGGCTT-TTGAGA-----------------------TGGGGGTAGGTTT-CGGCTTACTTGCGTCTTGGAAGCTGTTAA--------------CGAGATACCGTGGTCTTTGTCTGCTGGTTTGTATCGATGCA--TTGTTGGCCGCGTGTTGGCAGTGTGTTG-------------ATG-----CCTCGCAGGCAGTTGATATCAATCAACTTAGAACTGTCACTGACTAGGGGAATCCGACTGTTTAATTAAAACAAAGCATTGCGATGGCTGGAAA-CGGTGTTGACGCAATGTGAATTCTGCCCAGTGCTCTGAATGTCAAAGTGTGGAGACACAACCAAGCGCGGGTAAACGGCGGGAGTAACTATGACTCTCTTAAGATAGCCAAATGCCTCGTCATCTAATTAGTGACGCGCATGAATGGATCAACGAGATTCCCACTGTCCCTACCTACCATCTAGCGAACAAACAGTCAAGGGAACGGGCTTGACTGAGACAGCGGGGAAAGAAGACCCTGTTGAGCTTGACTCTAGTTTGATGTTGTGAGGAGACATGAGAGGTGTAG-AATAGGTGGGAGGCGTA----GGTGCTTGCATCTATGTCGACAGTGAAATACCACTACTCTTATCGTTTCTTCACTAAATCGGTGATGAAGGAAGCGCCCTCTTGC-AGGTGTAATCG-TAACGCTGCTT------CACGACTAGTGG----AGGCTGGGGGTTTACTCT--CAGCTTTAGCTAGTTGAGGAGAGCTGATTATTACCGAAGAGGATACGCCAATT-CCAGTAAGCATTAAGATG----GGCTGCCTTTGATG-----GGCGCATCTTGGCGCAAGTTAGGGTGCGTT-TGTTAAAGGCTGGCTTTTCGAGAGTGTGTCCATCGACCTTTCCGTGGTCGTGCAGTAATGTGCGGTCATGGGTTCG--TATGACCAATGACAGCATCAAATGGGGAGTTTGACTGGGGCGGTACATTCTTTAAAATGCAACGAGAATGTCCTAAGGTGGGCTCATCGTGGACAGAAACCACGAGTAGAGTATAAGGGCAAAAGCCTGCTTGATTTTGATTTTCAGTACGAATACAAACCTCGAAAGAGTGGCCTAATGATCCTTTATATTTAGCTGAGAGTTTCAGCATAGAGGTGTCAGAAAAGTTACCACAGGGATAACTGGCTTGTGGCGGCCAAGCGAACACAGCGACGTCGCTTTTTGATCCTTCGATGTCGGCTCTTCCTATCATTGAGCAGCAGAGTACTCAAAGTGTCGGATTGTTCACCCGCT-TCAGGGAACGTGAGCTGGGTTTAGACCGTCGTGAGACAGGTTAGTTTTACCCTACTGTCTCTTGTAATGCGTAGCAACAGTAATCCTATATAGTACGTGAGGACCTGTAGGTTCGGATGAACGG-TGACGCGTTTGGTTGATAAGCCAGTGATGCAAAGCTGTCATCCGTAGGATAATGACTGAACGCCTC-TAAGTCAGAAGCCAGTCTG----GAGCTATGCAAAAA-CCTGACTTTTATTAGTGAAAAAATACACG-------------------CAAGTGTGAATCCATCGTGGTGGCTGGTTTA-TTTAACTAGTCATTAGAAGTGTGTAACA--------------TTTCGAGCGATGTATATATAA----GTTGGGTAATATGGTTGCGTGAAGACGACTC--GCAACAGTCGCGGTGCTGTAAGGATTCGAGTGGACTTGTTGCCATGAGATCTTGAGGCTCATCCGTCGACTTGT-AACCAAT

Sphaeromyxa_zaharoni GGACCTCAATTCAGGCAAGAGCACGCGCTGAACTTAAGCATATTGCTAAGCGCAGGAGCAGAAAATAACAATGATTCTCATAGTAACTGCGAGTGAAGTGAGAAGAGCCCAACGTTAAAAGCTGCGTT----------TTGACAGACGTTGCATTGTAAC-GTATAGATGTGTTCTCAAAGTAAAAACAT--ATGTCAAAGTTACATAGAGTGGTACGCCATAGAGGGTTATAGCCCCGTGTATGACATGTGTAGTAATTTTACTAAGTAAACACAATCTAAGAGTCGTGTTGTTTGGGATTGCAACACTAAGTAGCTGGTAAACTTCAGCTAAGGCTAAATACCTACACGAGACCGATAGCAAACAAGTACCGTGAGGGAAAGTTGAAAAGTACTCTGAAAAGACAGTGAAAAGCACGTGAAACTCTCTATAGGGAAGCAAACAGGCGAATCGA-AGCGATATGAAAAGAGAGAGCCAA------------------------------------TTAACGCTATTGTTTCTCCCTACATGTTGCGCGC--------------CGGGCTTGTCGTG---TTAGTATGAATTTACGATAAAT--------GTACGGCATTAA---CATTAGTATGATTATAAGACGGTT--------AAATTATTAACTGAATTAAATCGTATAAT--------TTTACTTGCTATAAAGTACA-TAACATGACAAAGGCTAATATAAGTAAGCAGTTAGTGGAAGCGAA------TATGTGACGAAAAGGACTATAAGCTGTTAA-GTTTGTAGTACTT---TGAGTTTTTATAGCCAAATTAACTTATTCTCGGCG-AGATATGCGTCAGTTTGACCCGTCTTGAAACACGGACCAAGGAGGCTAACGTATACGCGAGTCAAGGGGAAACATGTATGTGTGTTATTCGATAAAACCCTATGGCGTAATGAAAGTAAAAAACGATATCTTTTGAT---------------TGCGTTATGAAGAGAGCATCGTTGCCCATTTGAA------TTCATTGGAATTTTAAATGAGGTGGAGCGTATGCGATAGGACCCGAAAGATGGTGAACTATGCCTGAGTAGAATGAAGCCAGAGGAAACTCTGGTGGAGGTTCGAAGCGATTCTGACGTGCAATTCGATCGTCAAACTTGGGTATAGGGGCGATAGACTAATCGAACCATCTAGTAGCTGGTTCCCTCCGAAATATCCCTCAGGATAGCTGGAGCCTGAGC-CAAAAGCAGTTTC-ATCGGGTAGAGCTAATGATTAGAGGAATTG-GGGTCATAACAGATCTTAACCTATTCTCAAACTGCGAACCGGTGAGAAAAATAATATTGCTTATGAATATAACTTCGGTTA-----TATGTTGAATGTTAT--TTTTGAATGCTAGGGCTCCAAGTGGGCCGTTTCTGGTAAGCAGAACTGGCGATGCGGGATGAACCGAGAGCCAAGTTAAGGTGCCTAAATAAACGCCAACG-GACACCACTAAAGGAGTTGGTCGATGAAGACAGCAGGACGGTGGCCCTGAAAGTAGGAATCCGCTAAGGAG-TGTCTAACAACTCACCTGCCGAATCGACTGGCCCTGAAGATGGATGGCGCTTAAGCGTTTTACCGATACTTGGCCATCAAC--ATTAGTGTAATAATATATGTTGATGGGTAGGAGGGCGCAAAGATAGTGACGAAGATTTTGGCGTAAGCCTAATTGGAGCTGTCTTTGGTGCGGATCTTGGTGGTAGTAGCAGTTTATTCAAGTG----AAACGCTTGAAGGCCGAAGAGGAGAAGGGTTCCGCGTAAACATCATTTGAACGCGGGTTAGTCGGTCCTAAGTGGCAGCTTACGTGCTGATAAACAAAGGGGAAT------ATCTG--------TATAAGTTTGTGATATTGATTAAACCCTTTATTACGGCTTATATTGGACTCCCAGTCCACGAAAGGGAATCAGGTTAATATTCCTGAACCAG--GTGACGGATAGTTTATATAGTGAATG----------TATGTTGAGGCAACT-----TGATGTGCATTTGTTATGTAGATGCAGCGGTAACGCAAACGAACTCAGAGACATCTTAGGCAGTCCTAGGAAGAGTTATCTTCTCTGTTTAACGAAATGA--CGCCCTGGAATTGAGTCATTCAGAGATAGGGCCGAGATTTCGGAAGAGCATCGCACTTTT-TGTGATGTCTGGTGCGCTGTCTAAGATCCTTGAAAATCTGAGGGAGTG-----AATGATTATCGTACCTGGCCGTACCGTAATCCGCAACAGGTCTCCAAGGTGAACAGCCTCTGGCA-CTAGAATAATGTGGGTAAGGGAAGTCGGCAAAATAGATTCGTAACTTCGGGATAAGGATTGGCTCTATGGATTGGGCTAATCGGGTTAACTTGCTTAATTTAAGTGCGT-------------ATGTCG-----------------------TTGAGTTTAATTT-CGACACGCAGTAATGTGGATAGTTATTGAATT-----------TGGCTACTTATGTGCTTTATTAGGTAGTTAATTTGGCAAGT--ATATAGGTGTTGTTTACTCAATGTCTAT-------------TTGCTTGTCGTTACGGTTAGCTAAGATCAATCAACATAGAACTGTCACTAACAAGGGGAATCCGACTGTTTAATTAAAACAAAGCATTGCGATGGCTAGAAA-TAGTGTTGACGCAATGTGATTTCTGCCCAGTGCTCTGAATGTCAAAGTGTGGAAACACAACCAAGCGCGGGTAAACGGCGGGTGTAACTATGACTCTCTCAAGATAGCCAAATGCCTCGTCATCTAATTAGTGACGCGCATGAATGGATCAACGAGATTCCCACTGTCCCTACCTTCCATCTAGCGAAACCACAGTCAAGGGAACGGGCTTGACTGAGACAGCGGGGAAAGAAGACCCTGTTGAGCTTGACTCTAGTTTGATATGGTTAGGAGACATGAGAGGTGTAG-CGTAGGTGGGAGACAAG----GTAATTTATTATTTTGTCGATCTTGAAACACCACCACTTTCATTGTTTCCTCACTTAATCGGTGATGAGGGAAGTGCCCAGTGAA-TGCCATAATC--TGTAGTTTCTA------CATGATCAAAAGTC----------------------ATGCTAGATCTACGCGTAGGTTGTGGTTG------AAGTTGGTGTCACGTCTA--CTAAGTATTGTAAAACA--ATTGGTGCTGCTAGTGCTGCCATGTATACATGAAT-----------------GTAGTATTAGTGGTACTTTAACT----AATTGTTGACCCCAACTA--------------CGTTTATCGTGTG------TGTGACCGATGACAGTGTCAGATGGGGAGTTTGGCTGGGGCGGTACACCCTCTAAAATGCAACGAGGGTGTCCAAAGGTGAGCTCACGGTGGACAGAAACCACCGGTAGAGTATAAGGGCAAAAGCTCGCTTGATTTTGATTTTCAGTACGAATACAAACCACGAAAGTGTGGCCTAGCGATCCTTTATAATGACCTGAAAGTTTCAGGATAGAGGTGTCAGAAAAGTTACCACAGGGATAACTGGCTTGTGGCTGCCAAGCGAACATAGCGACGTAGCTTTTTGATCCTTCGATGTCGGCTCTTCCTATCATTGCGCAGCAGCATACGCAAAGTGTTGGATTGTTCACCCGATAACAGGGAACGTGAGCTGGGTTTAGACCGTCGTGAGACAGGTTAGTTTTACCCTACTGTCTCTTTTAAGGTATCGCAATAGTAATTCTACTTAGTACGCGAGGACCTGTAGAATCAGATGAACGG-TGGCGCGTTTGGTTGATAAACCAGTGACGCAATGCTGACATCTGTAGGATAACAGCTGAACGCCTC-TAAGCTGGAATCCTTGCTGTA-ATAGCGATGCCATGTATCTCAACGCAATTAGTGAAAGAATACATA--GCTATTTATAGTTATGATGCTCCATTGCCTAGTATATTGAAGGAATAGTTCGGCTTGCCCGATAAATATACATAGTATGTGTCAATTACGGCATAACGCAATGATGATTGAC----GTTGGGTTG-CTGGTTACGTGAATACGACGC--GTAGCGGTCATGGTACTGTAAGGATTCGAGTGGACTTTGCGCCATGAGGTCCTGAGGTTCATCCAATGACCTGT-TACCACG

Polypodium_hydriforme_USA CGACCTCGAGTCAGGCGAGAGCACCCGCCGAACTTAAGCATATCGCTAAGCGGAGGAGAAGAAACCAACCGGGATTCCCGTAGTAACGGCGAGCGAAGCGGGAGGAGCCCAACTTTGAAATCTCCGCCGGGCCCCCCGGGCCCCGGCGGCGAATTGTAGT-CTGGAGAGACGCGCCCACGGCCGACGGTCGCGCGCCCAAGTCGCTCGGACGGGCGCGCCGCGGAGGGTGAGAGCCCCGTATGTGCAGGCGGCGACCGGGCCG--ACGAGCGCGTTCTCGGAGAGTCGGGTTGCTTGGGAGTGCAGCCCAAAGCGGGTGGTAAGCTCCACCTAAGGCTAAATACCGGCACGACACCGATAGCGAACAAGTACCGCGAGGGAAAGATGAAAAGCACTCTGGAAAGAGAGTCAAAAGTGCGTGAAACCGTCGGGAGGGAAGCGGATGGAGCTGTCCACGGTCCCGTCGCACCCGTCCCGTGCTGCCGCGCGC--------------------------GCGCGCGCGGGTCCCCTGGCGGGCCCGGGCGCGT-CGCCGCGGCGGCGCGGGCTCACG------TGCGTCGCGGATCGCGTCGGCCCCGATCGCGGGCCGTCGCGG---GGCGCGTGCCGGAAGGTGGCCGGCGCCCTGGTGCGCCGGCGTTACAGCCGGC-CGCGGTGGTCTTCCCTTAGCCCCGCCGGTCCCCGCG----CGATCGACGGGCGAGCCTCTCGTGGCTGCCGGTCGCGTG---------CTGTCCTCTCCAGGCGGGCGGCGCTCCCCC-CCGCGGGGTCGCC-------GCTCCGCCGGCACGC--GGGCGCGGCCGACGTCGATACGTCTCCATGCGACCCGTCTTGAAACACGGACCGAGGAGTCGGACGGTGGCGCGAGTGGAAGGG--------------------TGAGGAAACCCGGAAGCGCAACGAAAGTGAGAGAGGGGCAACCCGAGACACGGGACGCGCGCGCCTCGCGCTCGCGCCGCACCGTGGGCCGGCCCGAGGCCCGCTCGCGCGGGCCGGGCACGAGCCGGAGCGCCCCCGTCCGGACCCGAAAGATGGTGATCTATGCCTGGACAGAACGAGGCCGGAGGAAACTCCGGTGGAAGTTCGTAGCGATTCTGACGTGCAAATCGATCGTCCGATCTGGGTATGGGGGCGATAGACTAATCGAACCGTCTAGTAGCTGGTTCCCTCCGAAGTTTCCCCCAGGATAGCCGAGGCCGGTGA-CGTGCGCAGTTTCGACCGGGTAAAGCGAATGATTAGAGGCCTCG-GGGACGCGA-CGTCCTCGACCTATTCTCAAACTTTGAATCGGTGAGAGGCGCGG---------------CTCGCTTGCGTG-----GAGCCGCG---------CGCGGAATGCG-CAGCCTCGAGTGGGCCGTTTTTGGTAAGCAGAACTGGCGATGCGGGATGAACCGAACGCCGGGTTAAGGTGCCCAAGTCGACGCTCACGAGACCCCACGAAAGGTGTCGGTCGATCCTGACAGCAGGACGGTGGCCATGGAAGTCGGAACCCGCCAAGGAG-TGTCTACCAACTCACCTGCCGAATCGACCGGCCCTGAAAATGGATGGCGCTGAAGCGTCGCACCGATACCCGGCCGTCGCGCGGCGAGCGCTGTC----GCCCCGACGAGTAGGAGGGCGTCGCGGTCGCGTCGAAGTCCACGGCGCGAGCCCGGATGGAGCGTCCGCGAGTGCAGATCTCGGTGGTAGTAGCAAC-TATTCGAACG----GGATCTTTGAAGGCCGAAGTGGAGAAGGGTTCCACGTGAACAGCGGTTGGACGTGGGTCAGTCGATCCTGAGACGCCGGCCGCGGCCGCGTGGGAAGCGCCCGA--------------------GGTGGAGCGTCCGCGCAGCTCGCGCT----GCGCGGGCGCGCGTC---GGGCCCTGCGTCGAAAGGGGATCGGGTTAACATTCCCGAACCGG--GCAGCGGGCGAGCTCC----------------------------------------GGCGGCGGCAACGCCGCCGGGGCGCGGCGGCGACGCGGACGAGCCCGGAGACGGCGGCGGAGGTCCCCGCCGGGGTTCTCTTTCCCGGCTAACGGTCCGA--CACCCTGGAATCAGGTTGGCTGGCGATAGGGTCGAAGGATCGGTAAGGCACCGCGCTTCC-GGCGGTGTCGGGTGCGCCTCCGACGCCCCTTGAAAATCCGGGGTGGCGTGCGTGCGAAGCCTCGCTCCCGATCGTACCGACAACCGCATCAGGTCTCCAAGGTGAGCAGCCTCTGGTCGATGGCGGAACGTAGGTAAGGGAAGTCGGCAAGATGGATCCGTAACCTCGGGACAAGGATTGGCTCTAGGGGTCGAGCCGGTCGGGCCGGTGC---------GCGGAGGTCCGCGGCTCGCC-TCGCCCGCGCGCTCCCCTCCGCCCCG-----------------CGGCGCATCATCGCGCCGCGGGGCGGAGGCGCGCGCGGTCTTGCGCGT--------------------------------------CCGCGGTGCCGAGGCGCCCGGCTGCGTC-------------TCG--CGACGCGTCGGCCGGCGTCGAACGACCGACCTAGAACTGGTGCGGCCAAGGGGAATCCGACTGTTTAATTAAAACATAGCATCGCGATGGCCGGAGACCGGTGTTGACGCGATGTGATTTCTGCCCAGTGCTCTGAATGTCAAAGTGAAGAGATTCGTCCAAGCGCGAGTAAACGGCGGGAGTAACTATGACTCTCTCAAGGTAGCCAAATGCCTCGTCATCTAATTAGTGACGCGCATGAATGGATCAACGAGATTCCCACTGTCCCTATCTACCGTCCAGCGAAACCACAGCCAAGGGAACGGGCTTGGCGGAATCAGCGGGGAAAGAAGACCCTGTTGAGCTTGACTCTAGCTTGACTCCGTGAAGCGACATGGGAGGTGTAG-CATAGGTGGGAGCGCCG----CGAGGC----------GCGTCCTTGAAATACCACCACTCCGATCGTTTCTTCACTCACCCGGTGGTGCGGCGCG---------------------------------------------------------------------------------------------------------------------------------------------------------GGACGCGATGAGCGTCCCCCTTCTGGTACGAAG-----------------GGGCAAGGGCCCGCGCGCGGGGG----TTTCGCACCCCGGCGCGC--------------GGGTCGCCCCGAG------TCGCGTCGGGGACGCAGTCAGGTGGGGAGTTCGACTGGGGCGGTACATCTGTCAAACGATAACGCAGGTGTCCCAAGGCGAGCTCGGCGAGAACGGAAATCTCGCGTGGAGCAGAAGGGCAAAAGCTCGCTTGATTCGGACTTTCAGTACGAGTACGAACCGCGAAAGCGTGGCCTAGCGATCCTTTAGTCTTGCTTACGAGCTTTAAGGTAGAGGTGTCAGAAAAGTTACCACAGGGATAACTGGCTTGTGGCGGCCAAGCGTTCATAGCGACGTCGCTTTTTGATCCTTCGATGTCGGCTCTTCCTATCATTGCGAAGCAGAATTCGCCAAGCGTTGGATTGTTCACCCACTAACAGGGAACGTGAGCTGGGTTTAGACCGTCGTGAGACAGGTTAGTTTTACCCTACTGATGGTTCG---TCGTTGCGATAGTAACCCTGCATAGTACGAGAGGAACCGCAGGTTCGGACACTTGGCCAGTGCGCTCGGCCGAACGGCCGGTGGTGCGAAGCTACCATCCGTTGGATTACGACTGAACGCCTCTTAAGTCGGAATCCGGGCTAGATGCAACGACCGCACGTCTCCGAG----ACGCGCAGGCGGGCTTGAGT--------ACGGCGTGCGTGTCCGCGCGTGCGTTGCAGTGCCGTCGCGC-CCCGGTGCGCCGAGCAGGGCGTGCCGTATG-----------GGGCCGATCGATCGAAATCGCAGGCTTCTCGGAGG-GGAATCCTCTGCAGACGACTTAAGTATGGAACGGGGTACTGTAAAGGGTAGAGTAGCGACTCAGCTACGATCCTCTGAGGTTAGGCCTTCGTTCTCCGGATTCGT

;

end;

BEGIN PAUP;

Exclude [Positions with over 0.5 missing data]

129-136 161 191-192 456 490-516 551-564 580 603-610 623-625 650-657 678 687 689-694 715-718 761-766 797 811-817 833-834 849 919-938 986-1001 1036-1044 1262-1264 1277 1308 1363-1374 1390-1394 1405-1410 1513 1585 1667-1668 1681-1684 1793-1796 1902-1907 1913-1920 1947-1968 2013-2014 2041-2050 2067-2070 2171 2233 2282-2286 2352 2474 2481-2503 2517 2550-2560 2602-2603 2632-2644 2648-2649 2747 3036 3054-3057 3143 3154-3155 3167-3172 3185-3209 3238-3243 3262-3265 3281-3284 3299-3303 3318-3334 3353 3358-3361 3380-3393 3407-3412 3767 3892 3959 3979-3982 4027-4045 4077 4106-4119 4141-4144 4154 4176-4177 4252;

Exclude [ambiguous positions excluded by Guidance]

137 138 139 140 141 142 143 162 172 173 174 175 176 177 178 179 180 181 182 183 184 185 186 187 188 189 190 191 251 252 253 254 255 256 257 258 259 260 261 262 263 264 265 266 267 457 458 459 460 461 462 463 464 465 466 467 468 469 470 471 472 473 474 475 476 477 478 479 480 481 482 483 484 485 486 487 488 489 490 491 492 493 494 495 496 497 498 499 500 501 502 503 504 505 506 507 508 509 510 511 512 513 514 515 517 518 519 520 521 522 523 524 525 526 527 528 529 530 531 532 533 534 535 536 537 538 539 540 541 542 543 544 545 546 547 548 549 550 552 553 564 565 566 567 568 569 570 571 572 573 574 575 576 577 578 579 581 582 583 584 585 586 587 588 589 590 591 592 593 594 595 596 597 598 599 600 601 602 603 608 609 610 611 612 613 614 615 616 617 618 619 620 621 622 626 627 628 629 630 631 632 633 634 635 636 637 638 639 640 641 642 643 644 645 646 647 648 649 650 651 652 653 654 655 656 657 658 659 660 661 662 663 664 665 666 667 668 669 670 671 672 673 674 675 676 677 678 679 680 681 682 683 684 685 686 687 688 689 690 691 692 693 694 695 696 697 698 699 700 701 702 703 704 705 706 707 708 709 710 711 712 713 714 715 716 717 718 719 720 721 722 723 724 725 726 727 728 729 730 731 732 733 734 735 736 737 738 739 740 741 742 743 744 745 746 747 748 749 750 751 752 753 754 755 756 757 758 759 760 761 762 763 764 765 766 767 768 769 770 771 772 773 774 775 776 777 778 779 780 781 782 783 784 785 786 787 788 789 790 791 792 793 794 795 796 798 799 800 801 802 803 804 805 806 807 808 809 810 814 815 816 817 818 819 820 821 822 823 824 825 826 827 828 829 830 831 832 833 834 835 836 837 838 839 840 841 842 843 844 939 940 941 942 986 1002 1003 1004 1005 1006 1007 1008 1009 1010 1011 1012 1032 1033 1034 1035 1042 1043 1044 1045 1046 1047 1048 1049 1050 1051 1052 1053 1259 1262 1264 1265 1266 1267 1268 1315 1316 1317 1356 1357 1358 1359 1360 1361 1362 1363 1375 1376 1377 1378 1379 1380 1381 1382 1383 1384 1385 1386 1387 1388 1389 1395 1396 1397 1398 1399 1400 1401 1402 1403 1404 1405 1406 1407 1408 1409 1410 1411 1412 1413 1421 1422 1586 1638 1639 1669 1670 1671 1672 1673 1674 1675 1676 1677 1678 1679 1680 1685 1723 1725 1726 1727 1728 1729 1783 1797 1798 1901 1908 1909 1910 1911 1912 1921 1922 1923 1924 1925 1926 1927 1928 1929 1930 1931 1932 1933 1934 1935 1936 1937 1938 1939 1940 1941 1942 1943 1944 1945 1946 1947 1948 1949 1950 1951 1952 1953 1954 1955 1956 1957 1958 1959 1960 1961 1962 1963 1964 1965 1966 1967 1968 1969 1970 1971 1972 2015 2016 2017 2018 2035 2036 2037 2038 2039 2040 2043 2044 2045 2046 2047 2048 2049 2050 2051 2052 2053 2054 2055 2056 2057 2058 2059 2060 2061 2062 2063 2064 2065 2066 2067 2068 2070 2071 2072 2073 2074 2075 2076 2077 2078 2079 2080 2081 2082 2083 2172 2173 2229 2230 2231 2232 2350 2351 2444 2445 2446 2447 2448 2449 2450 2451 2452 2453 2454 2455 2456 2457 2458 2459 2460 2461 2462 2463 2464 2465 2466 2467 2468 2469 2470 2471 2472 2473 2475 2476 2477 2478 2479 2480 2481 2482 2483 2484 2485 2486 2487 2488 2489 2490 2491 2492 2493 2494 2495 2496 2497 2498 2499 2500 2502 2503 2504 2505 2506 2507 2508 2509 2510 2511 2512 2513 2514 2515 2516 2517 2518 2519 2520 2521 2522 2523 2524 2525 2526 2527 2528 2529 2530 2531 2532 2533 2534 2535 2536 2537 2538 2539 2540 2541 2542 2543 2544 2545 2546 2547 2548 2549 2550 2551 2552 2553 2554 2555 2556 2557 2558 2559 2560 2561 2562 2563 2564 2565 2566 2567 2568 2569 2570 2571 2572 2573 2574 2575 2576 2577 2578 2579 2580 2581 2582 2583 2584 2585 2586 2587 2588 2589 2590 2591 2592 2593 2594 2595 2596 2597 2598 2599 2600 2601 2602 2603 2604 2605 2606 2607 2608 2609 2610 2611 2612 2613 2614 2615 2616 2617 2618 2619 2620 2621 2622 2623 2624 2625 2626 2627 2628 2629 2630 2631 2632 2633 2642 2643 2644 2645 2646 2647 2648 2649 2650 2651 2652 2653 2654 2655 2656 2748 3037 3051 3052 3053 3057 3058 3059 3060 3061 3062 3063 3064 3065 3066 3067 3068 3069 3070 3074 3140 3141 3142 3144 3145 3146 3147 3148 3149 3150 3151 3152 3153 3154 3156 3157 3158 3159 3160 3161 3162 3163 3164 3165 3166 3167 3171 3172 3173 3174 3175 3176 3177 3178 3179 3180 3181 3182 3183 3184 3185 3186 3189 3190 3191 3192 3193 3194 3195 3196 3197 3198 3199 3200 3201 3202 3203 3204 3205 3206 3207 3208 3209 3210 3211 3212 3213 3214 3215 3216 3217 3218 3219 3220 3221 3222 3223 3224 3225 3226 3227 3228 3229 3230 3231 3232 3233 3234 3235 3236 3237 3240 3241 3242 3244 3263 3264 3265 3266 3267 3268 3269 3270 3271 3272 3273 3274 3275 3276 3277 3278 3279 3280 3283 3284 3285 3286 3287 3288 3289 3290 3291 3292 3293 3294 3295 3296 3297 3298 3299 3300 3301 3302 3303 3304 3305 3306 3307 3308 3309 3310 3311 3312 3313 3314 3315 3316 3317 3318 3319 3320 3321 3322 3323 3324 3325 3326 3327 3328 3329 3330 3331 3332 3333 3334 3335 3336 3337 3338 3339 3340 3341 3342 3343 3344 3345 3346 3347 3348 3349 3350 3351 3352 3353 3354 3355 3356 3357 3362 3363 3364 3365 3366 3367 3368 3369 3370 3371 3372 3373 3374 3375 3376 3377 3378 3379 3380 3381 3382 3383 3384 3385 3386 3387 3388 3394 3395 3396 3397 3398 3399 3400 3401 3402 3403 3404 3405 3406 3407 3408 3409 3410 3413 3414 3605 3606 3607 3608 3609 3610 3764 3765 3768 3769 3830 3832 3893 3960 3979 3980 3982 3983 3984 3985 3986 3987 3988 3989 3990 3991 3992 3993 3994 3995 3996 3997 3998 3999 4000 4001 4002 4003 4004 4008 4009 4010 4011 4012 4013 4014 4015 4016 4017 4018 4019 4020 4021 4022 4023 4024 4025 4026 4027 4029 4030 4031 4032 4033 4034 4035 4036 4037 4038 4039 4040 4041 4042 4043 4044 4045 4046 4047 4048 4049 4050 4051 4052 4053 4054 4055 4056 4057 4058 4059 4060 4061 4062 4063 4064 4065 4066 4067 4068 4069 4070 4071 4072 4073 4074 4075 4076 4077 4078 4079 4080 4081 4082 4083 4084 4085 4086 4087 4088 4089 4090 4091 4092 4093 4094 4095 4096 4097 4098 4099 4100 4101 4102 4103 4104 4105 4106 4107 4119 4120 4121 4122 4123 4124 4125 4126 4127 4128 4129 4130 4131 4132 4133 4134 4135 4136 4137 4138 4139 4140 4141 4144 4145 4146 4147 4148 4149 4150 4151 4152 4153 4155 4156 4157 4158 4159 4160 4161 4162 4163 4164 4165 4166 4167 4168 4169 4170 4171 4172 4173 4174 4175 4178 4179 4180 4181 4182 4253;

end;
