# Supplementary material for: Evolution of myxozoan mitochondrial genomes: insights from myxobolids
Source: BMC Genomics. 2024 Apr 22;25:388. doi: 10.1186/s12864-024-10254-w (PMC11034133; doi:10.1186/s12864-024-10254-w)
Supplement: Supplementary file 8 — Supplementary Material 8 [file 12864_2024_10254_MOESM8_ESM.docx]

#NEXUS

[Additional file 8 – Sequence alignment of the mitochondrial protein coding genes cox1, cox2, cob, nad1 and nad5 used for phylogenetic reconstruction. Protein sequence alignment, in Nexus format.]

begin data;

dimensions nchar=2395 ntax=14;

format datatype=protein missing=? gap=-;

matrix

AB731753_Kudoa_septempunctata ---------------------MFSCGSSSWWTWNLGFMSGLALSIQVVSGVGLALGYEDV---SANVRIEYAERHGDYWSWRRAHSIGATIYFIAMYSHMARSILSSSLAS-SSSW-SGCVLYFLSLTVALLGYSLTEGNMARWALTVVSSVVLSLP--AGESVYPVLVGNY--GISSAILPRIYAVHFLLGLLSPVVVVWHALEVHHNESSTDLSTRCGPSGKDFISGGGVKDSLVT--VFTAFLMLEGFDETSWIWSI-IEDMNANDWPQENKTPEPIGPEWYVLTLFAGLKLAS--VASILATVAITTWAYIRP-----REGSPWRMLALWITISC--------FLLGKIALKAHESLEEASLIAFM-LILASWS--ILAELRES------M--------------------IP------YTSQNLLSAKIVAIGYWRIAFLASILASACSCFLRVEISFLHGQSSESVYF-SLLTAHGLLFVFLVLVP-IGQGYLNWWFPGQTGTWDF---IWPRVNMGALLGVEWSTAILLLFWLPG-GWNPGWTMYPPLAS--FQQGFSIDCIIAGIHALGVASGAGSSNAVVSFR--LLILPFSRKELSIFVWSQFVAAVLFIGTIPSLALGLLGILLDRTTSSSWFDPSGGGDPVLYQLLFWFFGHPEVYVVVLPSFGVLSASL-E--ANSGLYGKEGLISSVSCLGVLGYLVYAHHMFTVDLELEVKLLFSSGTMAIAVPTGVKVYSWMVSVRSGSLD---TLQTFLPLLCFLATFIGGGLTGIMLSSSTADVLLHDTYFVVAHFHQVMAIAAFLAFACGVHWLGMMSH-----------------GGAIILFTIATMTIFLPLYGAGLCGIPRRVPSMLYE-----ASGFLVPGYLGCVLAIMAIILYLIFL------------------------------------------------------------------MLPFLFW-----SVLSSSLYAVALIVLWLTCVLL--AVA----FLKNNIL------AIGFFS------------------------TFFAKADALVICPITSVV---------------TAYVIP----------------GVEPFFHFFCHE----GATEVWHIRGQQWYWQYSKASLIWEEF------------------LLFRQQTDG------------------QFLQQS------TFPCFISAHRPVMVMGDALDVIHSWSVPDLGVKMDVIPGH---ITLDTFLPCLSG--FFTGACYEICGAYHALMAINVVVI--------------------------------M----------------------ISLVVVLALL--------------------------------------------ALSSGLGSLVDRRFLGLIGHRTGPLI---GYGFLTIVSDGFKLCAKNPWGSYFNL-LLGFLALGLETIPLAFMPYCLEP--------------GLGFLALLGMSSSV----ILLSSSL-FRGYSPLGRSRVRILLFLGEG-------------IFSFILF----SLALIMAED------SLFMMEQDSLVFFTFYAFPG-LGCSYFLLCLGERHPWDIPESESDLGGGFGVIYGGINFLWFAVLEYRWLIHWISFCFLM------KWAVLSHTINL----------FILRGLLPRYSFQALFKMLWKNLPWFILSWTFFVLFELNREDCFPAFIRVF-LFFPFGWRAFPVAFQGSWLFQMLFPWQSFFL---------------------------------MFSLLCLLLAMWVLSTQCAL-VRKTSLGRW-------WFILLSGSAFVFYFSDHLVWMCLSWSMMGFSSQAIVGR--RPTFPTRKSIASMVVHSTLSTFLCFSALF---STCADLDQEWKLALLFPRKTMSMIQSFICFLSLSVKSLLFPFSTWLISAMKGVALMSSLVHSLTIVASGLFLTTFL-LVEGWFWL-----GLVEWTSILFLPSSLWHLFYLLRENHGKRLLAWSTAFSVNLAHVLLPWDLR-----FSLLYGIFHGLSKSFLFA------------------CSQGKKDLSWSLLA--------G-LLGGFPLSFVWKVKTSFCHDS-LVASTFL------------------LWGLLAYLCVFLKLSF--K-----------TLLSVNKASLWVRFGQSWFTSAILLSLV---FL---------------------------------------------------------------------------------------------------------------------------------------------------------------

LC009436_Kudoa_septempunctata ---------------------MFSCGSSSWWTWNLGFMSGLALSIQVVSGVGLALGYEDV---SANVRIEYAERHGDYWSWRRAHSVGATIYFIAMYSHMARSILSSSLAS-SSSW-SGCVLYFLSLTVALLGYSLTEGNMARWALTVVSSVVLSLP--AGESVYPVLVGNY--GISSAILPRIYAVHFLLGLLSPVVVVWHALEVHHNESSTDLSTRCGPSGKDFISGGGVKDSLVT--VFTAFLMLEGFDETSWIWSI-IEDINANDWPQENKTPEPIGPEWYVLTLFAGLKLAS--VASILATVAITTWAYIRP-----REGSPWRMLALWITISC--------FLLGKIALKAHESLEEASLTAFL-LILASWS--ILAELRES------M--------------------IP------YTSQNLLSAKIVAIGYWRIAFLASILASACSCFLRVEISFLHGQSSESVYF-SLLTAHGLLFVFLVLVP-IGQGYLNWWFPGQTGTWDF---IWPRVNMGALLGVEWSTAILLLFWLPG-GWNPGWTMYPPLAS--FQQGFSIDCIIAGIHALGVASGAGSSNAVVSFR--LLILPFSRKELSIFVWSQFVAAVLFIGTIPSLALGLLGILLDRTTSSSWFDPSGGGDPVLYQLLFWFFGHPEVYVVVLPSFGVLSASL-E--ANSGLYGKEGLISSVSCLGVLGYLVYAHHMFTVDLELEVKLLFSSGTMAIAVPTGVKVYSWMVSVRSGSLD---TLQTFLPLLCFLATFIGGGLTGIMLSSSTADVLLHDTYFVVAHFHQVMAIAAFLAFTCGVHWLGMMSH-----------------GGAIILFTIATMTIFLPLYGAGLCGIPRRVPSMLYE-----ASGFLVPGYLGCVLAIMAIILYLIFL------------------------------------------------------------------MLPFLFW-----SVLSSSLYAVALIVLWLTCVLL--AVA----FLKNNIL------AIGFFS------------------------TFFAKADALVICPITSVV---------------TAYVIP----------------GVEPFFHFFCHE----GATEVWHIRGQQWYWQYSKASLVWEEF------------------LLFRQQTDG------------------QFLQQS------TFPCFISAHRPVMVMGDALDVIHSWSVPDLGVKMDVIPGH---ITLDTFLPCLSG--FFTGACYEICGAYHALMAINVVVI--------------------------------M----------------------ISFVVVLGLL--------------------------------------------ALSSGLGSLVDRRFLGLIGHRTGPLI---GYGFLTIVSDGFKLCAKNPWGSYFNL-LLGFLALGLETIPLAFMPYCLEP--------------GLGFLALLGMSSSV----ILLSSSL-FRGYSPLGRSRVRILLFLGEG-------------IFSFILF----SLALIMAED------SLFMMEQDSLVFFTFYAFPG-LGCSYFLLCLGERHPWDIPESESDLGGGFGVIYGGINFLWFAVLEYRWLIHWISFCFLM------KWAVLGHTINL----------FILRGLLPRYSFQALFKMLWKNLPWFILSWAFFVLFELNREDCFPAFIRAF-LFFPFGWRAFPVAFQGSWLFQMLFPWQSFFL---------------------------------MFSLLCLLLAMWVLSTQCAL-VRKTSLGRW-------WFILLSGSAFVFYFSDHLVWMCLSWSMMGFSSQAIVGR--RPTFPTRKSIASMVVHSTLSTFLCFSALF---STCADLDQEWKLALLFPRKTMSMIQSFICFLSLSVKSLLFPFSTWLISAMKGVALMSSLVHSLTIVASGLFLTTFL-LVEGWFWL-----GLVEWTSILFLPSSLWHLFYLLRENHGKRLLAWSTAFSVNLAHVLLPWDLR-----FSLLYGIFHGLSKSFLFA------------------CSQGKKDLSWSLLA--------G-LLGGFPLSFVWKVKTSFCHDS-LVASTFL------------------LWGLLAYLCVFLKLGF--K-----------TLLSVNKASLWVRFGQSWFTSAILLSLV---FL---------------------------------------------------------------------------------------------------------------------------------------------------------------

LC009437_Kudoa_hexapuctata ---------------------MFSCGSSSWWSWNLGFMSGLALCIQVFSGVGLALGYQDV---SAHFHIEDAERHIGIWSWRRAHSTGATIYFLAMINHIARSILSSSLAS-STSW-SGIVIYFLSLVVALLGYSLTEGNMAQWGMTVVTSAFLSLP--AGEFILPVIVGDY--GISSLILPRVYALHFLLGLISPIAVMWHSIEVHSNESSTELGTRIGPSGKDFASGGGTKDLLVT--ALTTILLLKGFDDTSILWRL-IEDMNANDWPQQNKTPEPIGPEWYVLPYFASLKLTN--IYAIMAALVIIIGAYL-------LQGAPWTFLGIILGWSC--------FILGKLALTAHESLEEVYVISFI-LLWTSWT--CWSYRRES------M--------------------LS------ASYSSSLSAKIVAIGYWRISFLASILASSCSCFIRAEISFPHGQTTEEIYC-SLLTAHGLVFVFLVLVP-IGQGFLNWWFPGQTGTWDF---IWPRVNIGGLLGVEWATAMLVIFWLPG-GWSPGWTMYPPLAS--IQHGTSIDVIIAGIHALGIASGAGSSNAVVSFR--LLLLPFSHKELSLFVWSQFVAAVLFIGTIPALALGLLGILLDRTTSSSWFDPSGGGDPILYQLLFWFFGHPEVYVVILPSFGVLSASL-E--ASSGLYGKEGLISSVSCLGVLGYLVYAHHMFTVDLELEVKLLFSSGTMAIAVPTGIKVYSWLVSVRSGSLE---TRPILLPLLCFLSTFVGGGLTGIMLSSSTADILLHDTYFVVAHFHQVMAISAFLAFACGVHWLGMVSH-----------------NGSLLLFTVGTMALFLPLYGAGLLGIPRRVPSMLYE-----SNSFLIPGYLGCIIATIAIITYLVFL------------------------------------------------------------------MLPLMFW-----NVLHSSLYAVALLVLWLSSIML--AMA----FMRNNIL------AIGMHS------------------------PFLAKLDSLVICPITSIV---------------LVYVIP----------------GVEPFFHFFCHE----GSTEVWHIRGQQWFWQYSKSSLVWEEI------------------LLSRQQSDG------------------QFLQQS------CSPCFLSAHRPVLVMGDSLDVIHSWSLPDLGVKMDVIPGH---ITLDTILPCVSG--SFTGACYEICGAYHALMAINIVII--------------------------------M----------------------ISFSLVLALL--------------------------------------------ALCSGLGSFVDRRFLGLVVGRTGPLI---GYGFLTLTSDGFKLCAKNPWISYGSL-VMAFLALGLETIPLSFLPHCLEP--------------GLAILALLGTSGSL----MLLSSSR-FRGYSPLGRSRIRTLMFLGEG-------------IFSFVLF----CLAFLIILD------GLFMMEQDSLVLLSWIAFPG-LGMSFLMMCIGERHPWDIPESESDLGGGFGVIYGGFSFLWFAVLEYRWLIHFISFCALM------KWAILSHTITL----------FILRGLLPRYSFQALANMLWWQLPWAIFSWSLLASFNLAREDCFLCWLEVFQAWFSYCFQGAPDAVYLGFYWLIQFPICQHYLD--------------------------------MLSLLCFLLALWAISTLCVL-VRKISLVVW-------WYILLSGSAILFYLADHFVWLCLSWSMMGFSSQAIVGR--RPTVPTGNALASMIVHSTLSTFFCFSALF---CICADADQEWKLSLVFPRKTMSPVHSLICFLSLSVKSLMFPFSSWLLSAMKGVATMSSLVHSLTIVASGLYLTTAL-LLEGVFWL-----RFVEGTIILFLPSSLWHLYFLLCESHGKRILAWSTAFSVNICHVLLPWDPR-----FSLLYGIFHGLSKSFLFA------------------CSQGQKSLSWSLMT--------G-LLGGFPFSFVWKVKTSL-EDS-IVSSTFL------------------LWAFLAYLFIFVKIGY--K-----------YIFSVNKSFLWEAYGQSWFSLAVLLSLV---FL---------------------------------------------------------------------------------------------------------------------------------------------------------------

LC009438_Kudoa_iwatai_Japan MKKKVHNPTRRKHTQTKKKTNLFSCNNSLLWTWNLGFLCGITLTIQIITGIALSMKYETT---TAFQNINTQEKYSNLWIPRRIHSIGTTTYFIGLYLHMIRSHIHQNMKN-SSGW-SGIIIFILSLGIALLGYSLSNGNMAFWALTVMTSLFTNLP--EGDFILSIITGNY--EINNTILPRILTVHFLLGITLPIMVYWHTSEVHKNESNTDLGTRGILAGKDFINTGIQKDLLFI--PIIGYFIIWGFQEESKLHKL-IEDNNTNDWIQKNKTPEPIGPEWYVLPFFIGLKTTN--LIGLITGIIFILLNMN-------KNIKRWNIITYWLCF----------FTLGDLALEAHESLLEPKVILFT-VMMLTLSGTLLTYS---------MNLLFFLPSLVYASDAFPFKLIS------FSKQNTISAKQVAIGYWRIALLSSIIATTCSGFIRIEISFPQVQSSEQTYF-SCITAHGILFIFLVIVP-IGQGYLNWWFPGQCGKWDF---SLPRINIGSMIGTEISLFLIIGSWLPG-GWSCGWTMYPPLSS--IQEGNSIDMIISGIHLIGVFGGLSSTNTLITFK--QIEYNQTYKELSLFIWSQCVTAILFIGTIPSLALALLGILCDRSLSGSWFDPSGGGDPVSYQILFWFFGHPEVYVVILPSFGVISTLL-E--SQSSLYGREGMIASISCLGILGYLVYGHHMFTVDLEMEVKLLFSSGSMAIAIPTGIKVYSWILSIITKKEN---LKSFSVPIICFIFTFVGGGLTGIMLSSATADILFHDTYFVIAHFHQVMAISSFLAFSCGIQLLGYKSH-----------------SGYLILFTIGTLILFFPLYYAGIIGTPRRIPSMTIE-----SQPFMFLGLIGYFLSISGTIGIMIHL------------------------------------------------------------------MISSLSW-----FLLVSSSHLVLYFILFLMLLVI--IVG---IFLCYS--------CISFFS------------------------LFFPFLDSLVVWPITFLI---------------VSYVIP----------------GFDPFVCFSCYD----PSFSVLHIRGQQWFWSYH-SSFSWIDS------------------VLCRNLSDG------------------SYLTFS------QFPCFLSVCFPVLVVGDSSDVIHSWGVSDLGIKMDVIPGH---VTLDLIYPLISG--LFSGFCYEICGSYHSLMTICLVIT----------------------------------------------------------------------------------------------------------------------MDRRFLGLVVFRTGIML---GFGFLSVLSDGFKLVSKNPFCSFFVI-VFSFFVFGLELFSFSFLFCVYEP--------------GSVFYSFLGVSSAF----IVFGLSFYFRGYSSLGRFRSQLQIYIGEG-------------VFSFIML----FFFCFLTFG------FVYCLFFDYVFVFSFYSISF-CLFSYLFLFHGERHPCDLPESESELGGGLGVTLGGFFFMIFVVMEYRWLVYFLLFCYLF------FWGFLFYAFFY----------FLFRGIFPRYSLYLMLRYIWLVFPIILLFWFLFFVF------------------FVYV-------------------------------------------------------MKELISFICFYVSLWVMVCHNLF-VRKGSPSKV-------WLWLLCLASLLFYSSTGVFYLIVLWSFMGIISSVIVFR--KPTYYNMVSSFSLVLVSFLSTFLGLSFLI---FVFLDKDQWLDLNWSYPSFPLSFHFSFFLFFFVSMKGLMFPFSFWILEAMKGISVMSGLVHSISVVFSGLYLFILL-VFEGWCLF-----SFLECYSVLFLFSSWFHSFLLFYDIYVKRILAISTSFSVNLCHLSFLWDFR-----FSIWYGFVHAFSKSFLFL------------------CVFSRVNFLWRLIS--------G-LLSGFPLTAVWYMKRYYLHHM-FPSFVFY------------------VWVMVVYLVLYVKLSF--K-----------FYLSCYKNIMVICYGRIWVTLSFLFGML---FIY--------------------------------------------------------------------------------------------------------------------------------------------------------------

LT671462_Kudoa_iwatai_Israel MKRKIHNPTRRKHTQTKKKTNLFSCNNSLLWTWNLGFLCGTILTIQIITGIALSMKYETT---TAFQNINIQEKYSNLWIPRRIHSIGTTTYFIGLYLHIIRSHIHQNMKN-SSGW-SGIIIFILSLVIALLGYSLSNGNMAFWALTVMTSLFTNLP--EGDFILSIITGNY--EINNTILPRILTVHFLLGITLPIIVYWHTSEVHKNESNTDLGTRGVLTGKDFINTGIQKDILFI--PIIGYFIIWGFQEESKLHKL-IEDNNTNDWIQKNRTPEPIGPEWYVLPFFIGLKTTN--LIGLIIGIIFILLNIN-------KNIKRWNIITYWLCF----------FILGDLALEAHESLLEPRVILFT-VMMLTLSGTLLTYS---------MNLLSFLPSLVYASDAFPSKLIS------FSKQNIISAKQVAIGYWRIAMLSSIIATTCSGFIRIEISFPQVQSSEQTYF-SCITAHGILFIFLVIVP-IGQGYLNWWFPGQCGKWDF---SLPRINIGSMIGTEISLFLIIGSWLPG-GWSCGWTMYPPLSS--IQEGNSIDMIISGIHLIGIFGGLSSTNTLITFK--QIEYNQTYKELSLFIWSQCVTAILFIGTIPSLALALLGILCDRSLSGSWFDPSGGGDPVSYQILFWFFGHPEVYVVILPAFGVISTLL-E--SQSSLYGREGMIASISCLGILGYLVYGHHMFTVDLEMEVKLLFSSGSMAIAIPTGIKVYSWILSIITKKEN---LKSFSVPIICFIFTFVGGGLTGIMLSSATADILFHDTYFVVAHFHQVMAISSFLAFSCGIQLIGYKSH-----------------SGYLILFTIGTLILFFPLYYAGIIGTPRRIPSMTIE-----SQPFMFLGLIGYFLSILGTIGIMIHL------------------------------------------------------------------MISSLSW-----FLLVSSSHLVLYFILFLMLLVI--TIG---VFLCYS--------CLSFSS------------------------LFFPFLDSLVVWPITFLI---------------VSYVIP----------------GFDPFVCFSCYD----PSFSVLHVRGQQWFWSYH-SSFSWIDS------------------VLCRSLSDG------------------SYLTFS------QFPCFLSVCFPVLVVGDSSDVIHSWGVSDLGIKMDVIPGH---VTLDFIYPLVSG--LFSGFCYEICGSYHSLMTICLVIT----------------------------------------------------------------------------------------------------------------------MDRRFLGLVVFRTGIML---GFGFLSVLSDGFKLVSKNPFCSFFVI-VFSFFVFGLELFSFSFLFYVYEP--------------GSVFYSFLGVSSAF----IVFSLSFYFRGYSSLGRFRSQLQIYIGEG-------------VFSFIML----FFFCFLTFG------FVYCLFFDYVFVFSFYSISF-CLFSYLFLFHGERHPCDLPESESELGGGLGVTLGGFFFMLFVVMEYRWLVYFLLFCYLF------FWGFLFYAFFY----------FLFRGIFPRYSLYLMLRHIWLVFPIILLFWFLFFVF------------------FVYV---------------------------------------------------------MLISFICFYVSLWVMVCHNLF-VRKGSPSKV-------WLWLLCLASLLFYSSTGVFYLIISWSFMGIISSIIVFR--KPTYYNMVSSFSLVLVSFLSTFLGLSFLI---FVFLDKDQWLDLSWSYPSFPLSFHFSFFLFFFVSMKGLMFPFSFWILEAMKGVSVMSGLVHSISVVFSGLYLFILL-VFEGWCLF-----SFLECYSVLFLFSSWFHSFLLFYDIYVKRILAISTSFSVNLCHLSFLWDFR-----FSIWYGFVHAFSKSFLFL------------------CVFSRVNFLWKLIS--------G-LLSGFPLTAVWYMKRYYLHHI-FPSFVFY------------------VWVMIVYLVLYVKLSF--K-----------FHLSCYKNIMVICYGRIWVTLSFLFGML---FIY--------------------------------------------------------------------------------------------------------------------------------------------------------------

LN86820X__Enteromyxum_leei ---------------------MFTCFPGTSWMWNLGFASGLSLMIQVSTGVVVSMNYNPG---NPYNDLDVVERDRGGWFWRGIHSAGASTYFVIMYLHTLRSLVINS-TN-FVAWSTGLLVLLWSLLTALLGYALTYGNMAHWALTVVSSVTLVLP--YGDYIQPNVVGGF--SVNMSMIPRVYSTHYLTGLCLLAVVSVHVHFVHEEGNNSELGVNSNLCSIDFLSAQLVKDSLTAWGVITLLCILQ--VKCHKLDVI-TFDPQVFRPVNTEAAPEPIGPEWYVLPPFGGLKITE--VPKLILMSVFVCMAST-------TYIRSRTPMILMIGVLL--------MILGIQAMTAHVNINMAEAVGLS-VSIIALS--VFIE----------M----------------------------FVRFGCLSAKVVAYIYWFTSFLFAILGVSLSILIRLEWSFPLFVSSGSLYY-STVTLHGFVMVFMVLVP-ATQGFMNWLLPGLLGCYDF---LWPRINLGSALGLGYSGFVMMFIWIFSSGWFSGWTAYPPLS---VTDGCLIYYEIGCLHAIGIFSLLATCNVLASFY--CSCSLGCTVEISLFVWSSVVSSMLFVMSSPCLVLALLMLVLDVMCSTMFYDCISGGDPVLYQTLFWFFGHPEVYVVILPVFGCVSLYL-E--SGGLIHGREGVVSSTISIGVLGFGVYAHHMYTLDLEVEVKMMFTAGTMAISLPTGVKVYCWSTSLLMRGSR---LLVSDVCVATFVVTFVMGGCTGIVLSACAVDEVLHDTTYVVAHFHQVMGVSAIVGLSCGLGFVRPVHS---------------LMCCCVIMFALGSSMVFIPLYLTGVSGVPRRCAVVSPD-----SSSLLSITSVGMVLSIASSIAGLCIL--------------MF----LVYACYVCCFCSCVLDDG------------------------LIFMFVDSGTWHCLNVFLTLSSMHLMSLMIVMFLILLF--TSSW--LF------------CCHFCNIES------------------------RSIEGFVLGVPQFLI---------------LSCFIP----------------GV--MGSSGCRL----SSGPVLYVEGSQWYWTYGFCGEVYESR------------------VLALSTSDW------------------GFPLFA------DTCIELPIGISIWVVCTSSDVIHSWSVTGLGVKIDCVPGH---SSLGSLLVMVPG--LYTGACSELCGVLHPFMPINLMFC--------------------------------MYQCLCWGLVKVSIVLLPLLCELVVLHLLISLYLYMLDWLSVSPSLSTVDLDCYGELKSYIAGSVLWLIGLSYIGSCMLFVGLASLLDRRFLASYVYRSGPLS---VFGIGTVASDGYKLFSK--FYGLINV-----------DFAYSGVCFVFEVYNGVFDVKESVIVGGAVFI-YMSISVSY----WLFAVRR-FRGYSSLALLRGLVCLLVVDP-------------LYVLCSF----MFTVVGSIGSGDDW-------------YSLYTFGDCLVFCILTVLVFERSPGDVVESESELSGGLSCDVGFLDMLIVSLMEYRWLFVVLHVCALT--------GYWSPLVFV----------ILLRSYCCRFGFH------WYTLS-TIRIWSSLVMLNCA------------VIYLSCS------------VWGVLI--------MDSVPF-AEAPLKVS----LYFMI---MRMRLMFGLVIVVLYMWVSLSSLSY-SRSLMLSSG-------LITLLMVYSLLFYGVTSTAGVLLGWSGMGFVTGLSMFV--RCSSVGRVSMLWVMCVSVMSTYMLLVFVS---RCSFDIDD-LGLFLDVPYCVSS---LMYLLVPVLIKSLCFPFSMWLIMAMRGSSYISGLVHSCTVVFGGYYLIALL-RLYAVLSS-----SLFPLLSILMVSSLSLHSCLLLYSSSRKLVLGYGTCISVNASFSLLVWDPS-----YGLLYGIFQGFLKGYLFSVYG---------------CSSSPIVLAVSMF------------SLSIPGSLLYRLEAPFSLG--FTGHSWL------------------LWLSTLY--IYVCMSFTLS----------KSYDSVNRSVVMNEVSTFILGLGLLLMFVTW---------------------SRVEGGALGFA----------------------------------------------------------------------------------------------------------------------------------

MN714000_Polypodium_hydriforme_Russia MVNFM--------------LFNFLAPSRLSIWWTLGFSLGMMIVVQVLSGFCLACYYVPEVDVALNSVIYIIKEVKGGWFIHHIHSCGASVIFFLLYFHMLKSLFYYSYVSRRRVWWSGILIYIFLSLEAFLGYILPWGQMAYWAGTVITNLIQVIP-IMGGFLYCLILGES--NLGSATLRRFYVFHVLVPFFIFGLVLLHLYFLHEKGSSAPYFWVSSFRFARFYPTLFFFDLFSF--CLIFLVFLV---FVFQFPYI-LGDVQNWVPVNSLQTPVSIVPEWYFLWDYAVLRCIPSKVLGVLILVFFYLLLILFPLLKG-VKDIMQGAINPVYCIFS--------LALFIGFFSLSCLGATPPEWPYIGVTRFYLFYTIVLFFGGLFFLRFWM--------------------FEVLICYRSYWFYTRNHKRVGVLYIGLAGFSSFIALSLSLVIRWELLSPGILNINPHYYNVVVTAHGVIMLFFVAMPALFGGFGNILVPLMLGCKET---YYPRLNNLSFYFNIPGFFLFLLSLVWG-GCGTGWTFYPPLSSVPF-HLTCVDLAILALHFAGASSILSSFNFIATVH-MLKPRGVSWFDLPLFVWSIYLTSWLTMFAIIVLSCAFFSLLSDRWGYTCLY--IQGGDPTLFAHIFWFFGHPEVYILVLPVFGLVSHILQGSLNREDLYGRRGMILAMMCIAGIGFFVWGHHMVTMNMSLYIIQYFSIMTAFISIPTGIKIFSWGMMLRGGDFSRLYQNPALLFILGFLFFFTIGGLSGLLLSNSKLDYYYHDTYFVVAHFHSILSLSVVYGIFSGLYFWDREIF------GGSINCV--LGVSHFVITFVSSLFTFVPMHVLGVYGMPRRYFDYDYNPGLQVWHVLTTVGSLFALLSVLYGVSILIGPIKIFGR-LEVR-KFMGISR--------------------------------------------------------------------FKEYGVGRVLMLRVGSFGWELRFL-----------NISFEGREESRVIFQLRLKLPFFLIR-------------IIYREEVVYEVFWSVF-------------PLMLLMYFTSFS-----SIFHFFD-RCYLR---GDLNYLQIWGKQWDWSSQLCRSYGEDN------------------FCFPLISESY-----------------------K-SD-IYNLIICSSGKVQGISFKSFDVLHSFFMSS-GFKVDVLPHS----RLNVLDTGDME--MAEFNCSELCGRYHSYMGGSIFFVSGDSLDIMVM---------FGYVIKCL-----M----------------------VLVFILLVFC-F---------------------------GFFNFLVALGYIVGVLVSTAYLTLLERKLMAGVQRRQGPNVVGKFYGLLQPLVDGVKLILKRS-PKLIKV-NVYYYISPLASLSFSLLPWGVVP-LGGGNFVENTERYSLLWV--LLLSSVQVFPLLCAGVSS-GSTYALLGGLRAATQFISYEVILGLSYIGFIMVLQFFFCRNP-FSLYSFLGVSG-----------LLLVLLPFSF-------ILLIVVFAEAHRAPFDLVECESELVSGYHVEYSSTLFASFFLAEYINILLHSILIGIV------LFGVSNVVIIL----FVCILFILVRCAVPRLRFDQLIDLCWNCLLPLLLIFLVVEGVGLA------------IVYYV----------------------------MKY-----GGYLGEGVVYCLYLSEKVEVGIRLGLIQVSMVVLVFVITGAVLWYSKGYMVMYPERKFFFILMFFFSFSMCLLILSPNIVQFYVCWELIGVASSMLVGFWRYRPSSFRSSMLAAVVNRLGDICLGFVTLC-----CLKSGN-LDFPVVVSFTD---FNLCLILVAGMVKSALFPFSFWLVAAMEGPTPVSALLHSATLVVAGVFLFLSFSPVITDFSSSQSAGWWHYSMLIIGLFNALYGAICASLQWDLKKLVAYSTISQLGFAVVSLGLTAKVHGEVLCCYYLFLHGVFKASLFMLVGIWAIVIRGQDMRAAPRISSKVSRFLLLYV--------GLCLMGAPFTPGHGVKLVLFEVC-YNSYYIV------------------FWGLMLASSFTS--FYCIK-------LFYWGLRVSS----WGEFGRGFMHGAVLILLSLG-LLHR--ELPLYF---------------LFFDNFCCCTWFVEVVFWSALCFGGVLYQLMRLSRIPVIIRNLFLKGNPLLWLEGLLFLVFLMAKGVLHFEKFLRLFFDI--TGSRFKVSFW---------------RRVRLNPISFTVFLVFVFFLFCLLLWW

MN794187_Polypodium_hydriforme_USA MVNYM--------------LFNFLAPSRLSIWWTLGFSLGMMIVAQVLSGFCLACYYVPEVDVALNSVVSIVKEVNGGWFIHHIHSCGASAIFFLLYFHMLKSLFYYSYTSRRRVWWSGILIYIFLSLEAFLGYILPWGQMAYWAGTVITNLIQVVP-VLGGSLYCLILGGS--NLGSSTLRRFYILHVLIPFFIFGLILLHLFFLHEKGSSTPYFWASSFRFVRFYPTLFFFDLFSF--CLIFLIFLV---FVFQFPYL-LGDVQNWVPVDSLQTPVSIVPEWYFLWDYAVLRCIPNKVLGVLVLVFFYLLLVLFPLLKG-KGRVIQGVINPIYCLFA--------LAVFIGFFSLSCLGATPPEWPYVRVTRFYLFYTIVLFLIALFFLRF-M--------------------LEVIICYRSYWFYTRNHKRVGVLYIWLAGFSSLIALSLSLLIRWELVSPGVLNLDPHYYNVVVTAHGVIMLFFVAMPALFGGFGNILVPLMLGCKET---YYPRLNNLSFYFNIPGIFLFLLSLVWG-GCGTGWTFYPPLSSIPF-HLTCVDLAILSLHFAGASSILSSFNFIATVH-MLKPRGISWFDLPLFVWSIYLTSWLTMFAIIVLSCAFFSLLSDRWGYTCLY--IQGGDPTLFAHIFWFFGHPEVYILVLPVFGLVSHILQGSLNREDLYGRRGMILAMMCIAGIGFFVWGHHMVTMNMSLYIIQYFSIMTAFISIPTGIKIFSWGLMLKGGDFSRLYQNPALLFILGFLFFFTLGGLSGLLLSNSKLDYYYHDTYFVVAHFHSILSLSVVYGIFSGLYFWDQEIF------GGSISCV--LGVSHFVITFISSLFTFVPMHILGVYGMPRRYFDYDYNPGLQIWHVLTTIGSIFALLSVLYGISILIGPIRVFKR--EVRLKLMGVPR--------------------------------------------------------------------FREYGVSRVLVLKIRSIGWEMRFL-----------DTSSVGREESRVLFQLRLRLPYSLIR-------------VIYKEEVVFEVFWSVF-------------PLMMLVYFTNFS-----SISHFFE-RCYLR---ADLNYLQVWGKQWDWSSQLCRSYGEDS------------------FYFPLIGESY-----------------------K-GD-IYNLMIGSSGKVQGVSFKSLDVLHSFFMSS-GFKVDVLPHS----RLNMLDTGSMG--VVEFNCSELCGRYHSYMGGSVFFVLDDSLDVMVM---------SGYILKYI----WM----------------------VLVFILLVFY-F---------------------------GFLKFLVALGYVIGVLVSTAYLTLLERKLMAGVQRRQGPNIVGKLYGLLQPLVDGLKLIFKRS-PRLIKV-NIYYYISPLASLSFSLLPWGVIP-LGGGNLVENTERYSLLWV--LLLSSVQVFPLLCAGVSS-GSIYALLGGIRAATQFISYEIVLGLSYVGFIMILQFFFCRNP-FSLYSFLGVFG-----------LLLVLLPFSF-------ILLVVMFAEAHRAPFDLVECESELVSGYHVEYSSTLFASFFLAEYVNILFHSVMVGIV------VFGVSSVMIVM----FVCVIFILVRCSVPRLRFDQLIHLCWNNLLPLLLFFLVIEGIILF------------IVCYV----------------------------MKY-----GGYLGEGVVYFLYLSERVGVGVRLGVLQVSMFILVFVITGAVLWYSKGYMFMYPERKFFYILMCFFSFSMCLLVLSPNIIQFYICWELIGVASSMLVGFWRYRPSSFRSSMLAAVVNRLGDICLGFVTLC-----CLKSGN-LDFPAIVSFTD---FQLGLILIAGMVKSALFPFSFWLIAAMEGPTPVSALLHSATLVVAGVYLFLSFSPVASDLSLSQGAGWWHYFMLIIGLFNALYGAMCASFQWDLKKLVAYSTISQLGFAVVSLGLTAEVHGEILCCYYLFLHGIFKASLFMLVGIWAIIIKGQDMRASPKVSSKVSRLLLLYV--------GLCLMGAPFTPGHGVKLVLFEVC-YNSYSFI------------------FWGLMLASSFTS--FYCIK-------LFYWGLRVSS----WGEFGRGFMHSAVLVLLSLG-VLHY--ELPLYF---------------LFFDNFCGYSWFVEIVFWCALCFGGFFYNLMRTSKVSVIFKNLFLKGNPLLWLEWFLFLGFLMAKGVLHFEKFLRMFFGL--LGSRFKMFFW---------------GKVRLNPISFTIFLVLIFFLFCLLLW-

Myxobolus_honguensis MLLLF-----FK-------IINFHSFYNFSFFWNFGFIIFFIFIIQVFSGIILSFYINFDLKYILISKIFLLNNISYGWFFLKIHCIFPNFIFLLIYLHIFKSLISFMFYP-LKLFISGIFLYLFFIIVVFTGYSIAGGSMGYWAIIVVFNILKYI---LGYEISSCIFFFDNIGVSSSTIFKLFSIHYLFSFFIFIFFFIHIFFLHDCGSSNNLQPG-YYDGFKFSCFIFFKDIFLL--FLFLFFIIF---LTFLNPSIFFILGDNFVEIQEFKS-VEIVVEWYLRFFFIVLRAINSKFFGIFFTFILFLFLFFYPLNKD-FFRFNYLISTFILFLFL--------FFYNLILFSWYFFSSYLYTIPFFILLFFTIFNFIIFNFYKFYYKF--M--------------------IN-----------MFNSRFISYLYFSISLFSGLLGFSLSVYIRIELNNPFSVFGDFYYY-VFLTGHGIVMVFYFLTV-LSSGFFCYLIFSFCFKNEDILLWFIKFSWVSIFFIFLSFIFFLISFFLGIGIDCGWTLYVPNSSKRFNLGFSLDFFIFSLVLFFLYLSFNSISVIFFFYEYCCKFVINFLNIPIFIWSNVLSSFLIYFSNPFFIVLAIMLFYDRNLFTYYFECEFGGSLILYQNLFWFWGHPEVYVLILPVFGLISQII-NCLC-GDLYNKVGIIFSMIFLFFLSFIVWGHHMFVVGWGSDIKGYFMQATIFISIPTSIKVFSWLRSLFFCYYR---FNIVIGFLLVFILVFIFGGMTGIFLSNYSLDIIFHDSYFVVGHFHTILASASIFGYLSVFFYYFKFNCFYS---FNNFYF---FSLFFFLFFTIFLLQLLINFHFLGFLNFPRRIHIYFIN-----YYFFFHLGSLG-LVGVWVSIYFFIILFLFF----------MF----IMFFNFLFNLY-FFF-NFFEMSNILFFYFSIFEFNYSSEFFYIYSSLLCFNLS--------------ICIFLIFFSLPFFKYVLG----FYNYNLFYDSC-FFFKFFDFNNFFMIGSFNYCINYFYFYKFVFKYFLYNGLFFKCNNYFFLDVISFIFYDF-CSNEFFLFIP-GIGFF--EFS-----WVNFFLDSFDFML-VKDYSFILIIFGYQWSWDFVFFIFDLVIF------------------DYFLCFKDGFTFFCYKYSLGCY-FSIKDFGF--------NKIWVVPFLENILFKGYSFDVNHCFYLSNCGIKYDVFEERSFSFFFNFNILYVKF--YYYFVCYEYCGKKHYDMYGFFFVLDGFWFNIIFKRECLVFG---KFYKL------FM----------------------IIIFFIIIVY--------------------------------------------FFILGFISILERKVLAIIQVRVSILRFN-VFSFYQFYLDFLKVLFKGFLSNFYFY-ICLYFFILLFLFLIIILFFLILF-LNLFYFIYSINFFNVFFI--LLLDVFIFFYKIFFILFF-KYKFFDYFVFKIKFIFIFLEG-------------LFFYFIF---FIFLLIYFF-------TNFINCFFILFFYFI-------YLYIFFLIKIFKGPFDYFEIESELVGGGVLNMFSFSFLLWSLIEYGLILNNIIFLFYLFNFFFYIFNIFIFLLII---LIFLFLTFFIRGVLLRLNFNSLIYF-------FLYFYYIFNFFIF-----------------------------------------------------------------MELFN----YFIFNIFILSFIIIFLIFCKIFFSNVNVFNKKFFLLLFLFIIMVFLSVICVL---SSDLKFSLFCLVFLSFYSMFLILF----NYGVGFKIKVVILFKVFADLLVLVVFG--FFIFFHINN-LNFFDFLDFKFC-RFIFIVLLIIFLIYSLFGVFGFWIYWAMEANYFLSSYIHSCGFIAIGLVFFCKL-SFFDFFNSNYLFEYFLIFINNFSILSYCVFISFFLLVKDVKKSFAYFSSSSICLLFFIIINYPV-----YGIIYFFNSSIIKILLFEIMSR---------------------------------------FKKFNLFISFIFICFFILN--FINFYLF-------FIYVKYSVLCNLFNFILFLKFFSIIFFLIK-------LYFFNLFDLS----YNFCLKFKYFLFFLIYFII--FLIFYIENFLYYFFFILDFDLNS----FFYSSF---CFFIIFLIKLYFYFFGYYDFIFIKKFFNFFFYNDIR-----------------------FLIKYIKSFIFFFSKYLKFPIFFY--NNYFFNF-LNK--LCLSFYIISFFIFLILIFLIV------

Myxobolus_shantungensis MFILF-----RTVNLLKNINLGFSVNFNLNLWWNLGFILIFLIIIQIITGIILSFFVELGFNFT-NSKFLLFKENKFFWIFIILHSIIPNFFFIILYLHILKNIIFPIYNT-LVMWISGVFIYLTYIIISYTGYSLI-TTIGFWALTVGKEVLEEV---LGGVIASLILFSDNIGICNFTFYKIFTIHYLISFFSIIIIIFHIYYLHFIGNSGGNVFI-HVNEVRFLIIGLSKDLLIQ--GIFSLILFY---NIFITPEN-FISGNFNLSFDKVFS-IEISVEWYLRIFFTLLKSIKIKRSGIFFTFIFIIIIFLIPYFKN-IWASSEIPLNFYLIIIF--------ITIFIILISNFYYSSFLSSISFILLIFFNIFFLFFLNYSYIKFNFIKM--------------------LL----------FTKNIKSLGIYYLIGSFFGGVIGFSFSICLRLELNNPFTVLGDYFYN-VCLTSHGLVMLFYFLTP-IIYSIYYIVIYNYNTYIKD---FLKLLSIFSYFLYLVSTFFVVFSLIISESVNGAWTLYTPLTNIDFNDSVGLDILLFGLNLFLISSIFNSIHIFWNSF-LIILKSESWLDAPIIVWSYFLTSTLSLISTPSLFSVLVLLILDRNFGTYFFNVDLGGSVILFQVMFWLKNHPEVYELLIPVFGLISKVL-EILCKGFLYNRIGIIYSKIVLFLISMFVFGHHMTTVGWGLDFKGFFMISTLIISLPTGVKVFSWVYSLMIYGFR---FSIITGFVSLFLIFFSIGGVTGVFLANYSLDLFFHDSYFVVGHFHMILVGSSIFGYLSGLFLVYSEILNFS----KDMWFIRIVWILFFIIFSLGLLQIIYFFHILGLIGNPRRVLLMSFN-----RNLNLHYSSIS-FLNIWFSFFLIIFLFLLRLVVIKI----MFYKSMVLFFFYIFNYL-FIFNNLFEFCNEIVFKKSLRIFNISLWDYYFG----------------------LVISTFIFFCLFFI--------TFY-----------IIKIFNKNNFNSL-NLDL------------IFFENKYHFNLISSINYLDYIAFSL--------CFLGIP-IFTIFTNSYS-----YGGLFVDIDKNL-----ISFILNVLTFQWGFDFYLLSFKPIYF-TRIFYPISKFIKNISISELFLNISNYNLLSEFNF--FIESNNFYNFEEVF------KINIIIPNRTPILIKTFSKDVNHCLGIIDLGLKLDSIGEK---ILVGYMNIFTTEGKQIDFLCFEYCGNKHSDMIGSITILNFLYLNFL-----------------------LM----------------------FTLILLFFVY--------------------------------------------IFIIGFLTILERKILGLMQNRLSVFKNS-FLSLYHFYLDFFKVFFKGNFGKEFKK-FSNYLFLLVLIFLFNLFEFYFLI----FGFKFDKFFFNILII-YLLIEGLIMILKILILNTI-KSTYISITIFRIKWLYILLEG-------------FFSYIIF----FNLIILNYI------KYYKLNLQLILLLIF-------IFYIIILLKIFRNPFDFFEVESELTGGIVLEFKGISFLLWSLFEYSSIFNSHLFLIYILSLYM-KSNIINYIFIF---IFLLTLTFIIRGVFIRFKYYSTICL-------ICFLYYLINLIFLF-------------IWFF------------------------------------------------MFYK----NFILIFLILNFLIILIIIFNLIFS--KEFSNKFYILGISILIMSWLNLYCVL---INTLEKTFCSLILLSFFSMFTLHI----GYGIGISVNIVLFFKIFGDLGLLVIYS--FFFIQDLETYIIYFNLLNYQL---FIKILVITVTSIYSLLGIFGFWIYWAMDASYFTSLYIHSCGFVAIGLVIFNKF-SFLDFFNENC---SFGFIIYYTSYYSCIFYLLIALYLKDLKKSFGYFSGSSICFLFWISLNNTT-----IFLTYFFISSLSKIIIFELLNF---------------------------------------LNR-NFHIKWFISYIITID--FISISVFLINPFNLYVYSK------GFYFIFFLKFYYLIVFLIK-------IIILSKDNHKEILHTNNHKTHTFIVLLIVLFYIFILILG---VKIFY----------SSIISFLYNNIILISLVFIITNILW---WGGFNIFFNIG---LLFKNYIKKI--------------------SNIIEYINKFSYNLNIGVKSKIYIFKCNNLNLNISFKNKLFKDSLFLILINILLFLFLTIIFL----

Myxobolus_wulii MFIFL-----FE-------LVNFPSFYNFGFFWNFGFIIFIIFFIQVFSGVILSFYINFELNYILLSKIFLLNNVSYGWILLKIHCILPNFLFFFIYLHIFKSLINFMYYP-FNLFISGIVLYLLFVIVVFTGYSIAGGSMGYWAIIVVFNILKYV---LGSEIASCVFFSDIIGVSSITITKLFSIHYLFSFFIFIFFFIHVLLLHRCGSSNNLHSI-YCDIFRFSSFIFFKDILFS--LLIFIFLIF---LTFVNPSIFFMLGDNFIEIQEFKS-IEIVVEWYLRIFFIILRAINNKFYGIFSTLIFISLFVLSIFSKD-LFRFNILPISFILFIFF--------FMYVLIRFSWYFFSSYIFSMPIFFLLIIYIF-FYFLVFFRIIYNV--M--------------------IN----------FMFNLRFISYFYFFISIFSGLFGFSLSIFVRVELNNPFSVFGDFYYY-IFLTSHGVVMVFYFLTI-LSSGFFCYLISNINLVENGGMLWFLKFSKLSLLSLIISLVFFLFSFFSGMGIDVGWTMYVPNSSRRFNTGFSLDYFIFSIVLFFLYLSFNSISVMFFFYNYCARFVFNFFNIPILIWSNTLASLLIYFSNPFFVVLAIMLFYDRNLFTYYFECEYGGSLVLYQNLFWFWGHPEVYVLILPVFGVLCQVV-ESLC-GDLYNKTGIIFSMIFLVFLSFIVWGHHMFVIGWANDIKLYFTQATVFISVPTSIKIFSWLRSMVFYKYR---FNIIIIFILIFILIFTFGGMTGIFLSNYNLDIILHDSYFVIGHFHTILASASIFGYLIAFFYYFRFNYFFN---NDDFYL---TFLLFFLLFSIFLLQLLINFHFLGFLNFPRRIHIYFIN-----YYFFFHLGSIG-LVGVWFGIYFFVILFLFFNS-----ENEMF----SIFYSFLCNFY-LLF-NFFNFVNIYFFYFSVFEFNYVGDFSYVYFNLLGFNFS--------------ICIFLFFFIIIFFNDIINS-RYFFHPNWPY-----------VGNFFMVGSNNYFVNFNFFY-VIFNFFEFN--LFSNYDYFFIDFIFFIFYFFNFESELFLLIP-NFGIL--GFS-----WVDLFLESFNYLQSLGRCQFILSVFGFQWSWDLHLYTFSHIYF------------------NFYYTSSHYFIRESFGFQSGIINWNLENFEE--------NRIWILPVFNNIFFRGYSLDVNHCFYIVGLGVKYDIFDERDSGFIFNFNIIFSKS--YYYFVCYEYCGRRHYEMYGFFYVLDGFWFSVIFMEEHDVFT---GRYFLNHNGGDF-----------------------MFFFIYILFY--------------------------------------------FLILGFMSILERKILALIQIRISIIRFS-LFSIYQFYLDFLKVLFKGLFDYFYIYNIFMFFFILIFIFLIIIIMFFMIV-LNLFYFFYRINFFEIFFL--ILLDIFIFFCKLIFIYFF-RHNFFEYFIFRIKFIFIFLEG-------------LFFYFIF---FFFLFIYLFD------LFFFFNLFILFFFLL-------FFYLIFLIKMLRGPFDYFEIESELVGGLVLNIFGLNFLLWSLVEYALILNNLIFLLYIFTFLIYYFSFIFFLILI---LFFLFLTFFIRGVLVRISLNSLIYF-------LIYFYLFFNLFLFFL----------ILIIFSY----------------------------------------------MYLLN----YLGLNVIILSCVIFFFILCKLFFNNINIFNKKFFILLFYFFIMIILSIICVL---SFDLKFSIFSLFFLSFYSMFLILY----NYGVGFKIKVVVLFKVFADLLILVIFG--IFIFFNIYD-LNFFNFLDFRFC-NLLLFLLVIIFLVYSLFGIFGFWIYWAMEANYFLSSYIHSCGFIALGLVFFCKL-NFFNFFNFNFLFNNFLIFINNFSILSYCFFVFLFLLVKDVKKAFGYFSASSICLLFFISINYPT-----FGVIYFINSSLIKVLLFEIMSR---------------------------------------VRKFNLFISWVFIFFFILN--FINFYLF-------YIYVKHLNLCNLLNFILVFKFFSIIFFLIR-------LYFFNIFSIK----VNLFLSYFFFNFLFIYFFVFLFLFFYIEYFLYYFFYYFYFIFFSNYICFFYFSI---SFFLIFLIRLYFYLFSYFSLFYLFSFFNLFFYNDLF-----------------------VLLIFIKSFIL----YLNFNLRFY--FNFSINLNFTG--LNLSFLMAGSFLIFFLFFIII------

Sphaeromyxa_zaharoni MGMKI--------SKNVKVVFNFPTWFHLGFSWNVGFVLFMFLILQILTGIIMGMFYDPSLKGALSSIIYVIKEVKWGFVIHHMHGVGASFIFAMLYFHMGKAFFYGLGVN-SSVWLSGFVIFLLLVVEGFLGYSIVLGSMSYWAIKVVVSMFSVMGGEMSEVLMQVLLGGD--FITDLTVRRFYVFHCIIPFVVVFFVFVHMAFLHKGGSNMLISCN---EAFKFYPYMFYKDVLLF--LVVFYVYIT---CVLLFPNF-FYNPVNFEIFNPNVTPEHIEPEWYLRPFFIILKGFYSKGLGIMVLIFIFVGLGLNTFSNNRIWKVSGVEAYFYFSFMIIGCFISYWLILGDAVVSWFHWFSALLVI----VCLVGEGGVVFMEMGLYGVS---M-------------------------FFYCTWMLVMDCRFVGVLYMFLSILGGCLGFGFSVQMRGELLYPFSVYGDEIFY-VCITAHAVLMVFFFLTT-LATGFSNFFIPGMFGVVDF---FFPRLNSLSFWMVLLSGLFLVFSLLVGNGVSTGWTAYPPLSNYGFSEGGSIEMFIMAVHLFTVSTVLSSINIICTVF-YSKLYYLDWLDVSVYVWSIVVTSFLNVASLPYFSSSVTMLFLDRNFNCVFFDPSYGGDPLLYQSLFWFWGHPEVYILIIPVFGLVALIMCNLLDVEYLFGSYCMVISIMSVSVLGFIVWGHHMVTVGWSLDIRVFFMVATVVISLPTGLKVFNWICSVYFYLVKDLLLEICYYYIFSFVVVFIFGGVTGVMLANSTLDVIFHDSYFVVGHFHYILSLAAVSGYFCLFYYSWGSII-----GGGDIDFT--FGIIHLGLFMSSANVCFFPFHLLGLAGLPRRVSCYPEV-----FFPSFYFSSCG-FLFVALSVFVFFSGILYSIN--------MLLRGSGLGG------I-CIHPDAF-------FTNISSSMSYKAGYPYLLFLMLGFSFVGCGTSLASFVTGVLLGKVVVMLTFVLM--LVG--------------CLSCLSFIEWEEVGVL--------------------ADIYSFIMGKKIDLFSYFCLSV-------------PIVSSVFVLSFTVNCISLTSSFLE----------ISFFIHLIGFQWGWEFYYPFFD--------------------IDFMYGLDEDGF----------------VEGLDIF------SPSFVFPVKTSCTVVLSSLDVIHSLGADVLGVKLDAVPGH---LNSVFFDSEVEG--FTGVVCSEYCGEGHSFMSLKMDIVS--YMDFFVLGYNVLGEEGEGFFVL------LM----------------------VVVILLIVIQ----------------------------------------LVLIFLSVGVVTVVERKVLSFVQGREGPCQ-G-LKGIVVFILDYFKLVFK--FGSFGGLFFSSMFCIFLLFCMVGLTPLSLLG--------FKGSFYNVLFV--LILDVILVGVKYVVSDSY-VSKMVEESSYRLRLSVLVVEL-------------MFFFLIFPVVFSFLYLGASGWLEASCKVSFFFIFLEFFLFL-------LGLSLSLFQVSRTPFDYFEGESEMVGGVLSHVGGGHFIIWSFVEYLEIYFKSLLLVSVFFPFIIKYSFVFSILVI----FLVLFFVLIRGLLPRLDYRSSIVY-WVGGGFVVVFVLLLDFMFVLL----------RLVF---------------------------------------MILGGG-------------SMLEDVVILSYCLSLMVLLGMKKVEGEGGGCQEI-LLFVHFLCVVLLLSCSV------LEWAVILYILGGSVGSVSILF-SMKDRGNGSFVKVGVLTLMLDVFLGVLVIA------------MSLDGGLEGSD---FYKLGILSVILFKSCFFLFGFWIYEAMDASYFISCFLHSSGVVFLGIVLLFKF-SI--YFNS-----GLFFWVKGMSLLGIVFNLILFFLDYDYKKQAGRLTCVSYNMMFLLFCIDVR-----LGVFYFIVHSFLKSIVFYLLANL-------------CVSGIVVKSSDAFLIVGGGVYLGILIGGL-LFVGYGVDLGLFVG---------------------------VLGLMEGMCYLSLLFKMLEVDY----------------LLYNLEGVRNLLNIMNLMFN---YLMS----VVVY------------------------KEMVEIIFVSFVSSFNKIISMFKFLSCDILFEVIVSK----------LFV-----------------------VGCSFKGIIK-MEGSKLIEACSLGRIVDQDFVVLFLLLLLMVLLI-------

Thelohanellus_kitauei MFLTF-----LERKLWWNLFFYFPTTISFSIWWNLGFLLLLFIFFQVVSGIFLSCFINIG-SFCLTSKFLIFKEIVGGWFLVIFHIITPWFIFFFLYLHILKSLLFPTYSV-LKLWSSGVIIFLLFIIIVFSGYSIVYGSMSYWAITVVSELIKFI---FGEKISSIIFYNTS-GISEITLFKIFTFHYFFSLLVFVFIFLHIYILHNLGSSFSIHS--YCSCFSFSLLLFIKDLCIG--LFLLIISIF---FVCFIPEI-FMSGEGNMTLDKVDY-VEISVEWYLRIFFILLRSFSSKFLGAYSTILLVLLLLLYLFFRN-IWGIQRC-VNFWLITFL--------TISVVVYISFYFFSSSLFLIPFLLVILFNLIFIQPGECYQSYYL---M--------------------INS---------FLLKDVHIKTVYLFLSLIGGILGFAFSIIIRIELNNPFLVFGEYFYY-ICVTSHGLVMVFYFLTV-LSVCFFHYFYYSYFNFKSYS--YFNSINKISLICLILSWIFIIISLFSGEGINTGWTLYVPLSLKEYNNLFCLDFLIVGLDLFLISSILNSTFIISSLY-FSFYISESFLDVSVLSWSYLLTSFLMLFSIPSLFCVLSMLLLDRNFNTFYFNCEFGGSLILFQSLFWIWGHPEVYILILPVFGVISQVL-EIINKGYIYNKNGIIFSMISLSILSFLVWGHHMATTGWGLSIKNFFMICTLIISLPTGVKVFSWIYSLILFGKS---FNLVSGFISLFIMFFSLGGISGVFLANYSLDIIFHDSYFVIGHFHTVLASASVFGLLSGFFLLNKKLLTISCVGGGSYKFLMFIWLGLFNSIWISIGQFLFYLHFLGFLGIPRRIVDLSFC-----FYFNSHILSVG-FLWIWFAIFILSGLILLSLNRRNVREEKMIKFFKILTFNYLTSF----LLESFD------FYFDIFCLSFKSATLFLK--GLYFNLS---------------VEFSVFVCIILI--IVG---FYF----------DILSFFSFENVLLL-SLNQSPS---------VKWKEGGNIILCKLKTLLGYFKLVF-PF-----LILIIP-VYCFFVSSHN-----SVVLCQEDTDY-----GATFYVFIYGFQWGWKVTITQVSSLSELYRL--------------TVQLLISDR---------GNIIGTDRHKFLDVYIKGDSKNLHIPLPSRSSIKVITTSEDVNHSVGSAELGLKIDLFQDR---LIIQDLCIYSDSGKEFKFDCFEYCGERHTTMWSIFTTLEEVELALSKK----------GF-------KKFM----------------------ITIILITFFY--------------------------------------------LVSVGFLTVLERKIIGIVQNRVSVFKFG-VLGFYYFFLDFLKVFLKNFFSKGSKK-ISSVVFLLFLLILLLIISFNLLIIGISVKNTLNVNFLGLFIIIYLTLDSFIFILKIFFICTF-KSKYVGISTQRLKLVYLFLEG-------------IFIFTSL----LFLVLGEFS------SNFCYIIF--------------VLFILGLLKSFRTPFDYMESESETVSGVLLELQGVGFLLGSLLEYGVIINNLFFIVFLLNL-----GFISGLFYL---IFFIFITSFLRALVVRVRIQTLFYI-------IYQFWVFFTIVVFII----------IVIFIF-------------------------------------MNIKKG----SMFLF----LLFLILFLLSFIVGVLLILKINFS--RSFILKFLNLGVFILFMIIVNLYCVI---HQDFRKLGMILIFLSLYSLFLILH----GFGVGFSVNIVVIFKALADLLLILFIG--LLYFYNYN--WDLTSHMTGGSS-ILLEVLLIVIFSIYSLLGLFGFWIYWGMEASYFVSSFMHSCGFISVGLILFIKL-TSYGYFCL----WYFKFFIIVISLFSYLFFISLFLKIKDIKKSFGYFSASSICFIFFLSIEHPL-----LSLFYFFISSLLKILIFELLCV---------------------------------------TLKVGLEF-WCFLVLSFLY--LSSFSILILVPFNNSFFIC------LYNYIFLFKLYCLFIFFLRVGNSLGGINVRSNKKYNKVYYFLNKGLGLLLMFLNIVFFLFCFL------KVIY----------KPSESFYLSSFLVNHFIFIFVIFLIWRCLGILKTFVSIEDWVFYFKKKLKH----------------------NIVSIFNTFFSISRSFIKNRFNIKWLVFLFLEKDLSNF---LGLDLFSFLFFIILIFFIL------

MK087050_Myxobolus_squamalis -----------------------------------------------------------------------------------------------------------------------------------------------------------------------------------------------------------------------------------------------------------------------------------------------------------------------------------------------------------------------------------------------------------------------------------------------------------------------------------------------------------------------------------------------------------------------------------------------------------------------------------------------------------------------------------------------------------------------------------------------------------------------------------------------------------------------------------------------------------------------------------------------------------------------------------------------------------------------------------------------------------------------------------------------------------------------------------------------------------------------------------------------------------------------------------------------------------------------------------------------------------------------------------------------------------------------------------------------------------------------------------------------------------MLYILTIILF--------------------------------------------FTITGYLTILERGILGLVHNRKGVSKIN-TFNIYHFYLDFLKMIIKGRCGKTISK-FNNLIILILGMLTLILFIFFLLP----FNAISKNITLSFIWL--IVLDNILLIYKIFLLERI-GGKIVKLTSKRIRKIYIILEGCMNL---------LYSPIIY----IYILFSSSSWGISQILLGKLSIIYLTPLYL-------TLLMVNLLKSLRCPFDYFEVESELSGGGTLEFGGINFVIWSLVEYSLIQNNIYILITIILI---IWGFINYSIIITLFIILSILIMLTRGVLTRVRFESIFMI-------LIKIQIILIVLSLGE----------IIIL------------------------------MFYNLISPNLIAKEG----IYLIIGSKLDYILIILPISILLIIYISSKSLSQYMKGFKHSDGVFSLLVFILLYISISKNL---------LIPELILSSFISIVLVLI----GFGINIKVLAIVIFKILADILLIIFYSGNMRVIIDIGEEGFLFP--------LINQNMLVLAVSIYSLLGIFSFWIFWGMDASYYISSYLHSCGFIIIGVIITYKI---FNYFSLKDI--GILIPIVNIGIASILFLSIIFINIKDYKKSFGIFSAISINYFYILIFISPN-----QAISYFIFSSIFKFLYFFILSN---------------------------------------INKFPTRI-YLIKIFVSLELLFYSFNLF-------LLGGK-GILGDLIEILILLKIIITPIYIIK-----------TINSLN-LGNINIIGYLKILILNIIFFLL--FLTN----------------SNTNSHHLIFS-------ILILIILIITWGTNLLITRIGIKNIEIIFRNIIYKF--------LIFIYLILQEKNYTHIRFIK-------------------KNLRITEKINRGGISLNM------IIFFLIVLII------

;

end;

BEGIN PAUP;

charpartition genes =

COB:1-394,

COI:395-926,

COII:927-1332,

ND1:1333-1779,

ND5:1780-2395;

Exclude [Positions with over 0.5 missing data]

6-19 61-63 112-112 158-159 173-174 239-240 250-252 261-261 298-299 318-322 340-347 371-371 389-394 396-415 418-423 475-475 493-493 513-515 543-543 558-559 593-594 683-683 685-686 758-760 829-843 882-886 913-926 929-978 986-998 1013-1014 1018-1020 1024-1034 1044-1064 1083-1097 1104-1119 1132-1135 1163-1180 1190-1207 1214-1219 1260-1262 1275-1276 1301-1332 1334-1355 1366-1409 1436-1436 1438-1438 1465-1465 1489-1492 1508-1508 1528-1528 1550-1562 1570-1573 1583-1588 1604-1610 1669-1674 1686-1689 1714-1714 1735-1746 1752-1799 1804-1807 1832-1832 1842-1842 1887-1888 1918-1920 1929-1929 1942-1942 1997-1997 2006-2010 2062-2066 2089-2102 2117-2124 2126-2126 2148-2148 2156-2173 2193-2203 2233-2234 2238-2261 2266-2272 2282-2284 2296-2298 2307-2370 2374-2379 2389-2395;

Exclude [ambiguous positions excluded by Guidance]

1 2 3 4 5 11 12 13 14 15 16 17 18 19 20 21 22 59 60 62 63 64 65 66 109 110 111 113 114 115 116 139 156 157 159 170 171 172 173 175 213 214 215 216 217 218 220 221 222 223 224 225 226 227 228 229 237 238 241 242 243 244 245 246 247 248 249 252 253 254 255 256 257 258 259 260 262 263 264 265 266 267 268 269 270 271 272 273 274 275 276 296 297 300 301 302 303 304 305 306 307 308 309 310 311 312 313 314 315 316 317 319 320 321 323 324 325 326 327 328 329 330 331 332 333 334 335 336 337 338 339 348 349 350 351 352 353 354 355 356 357 358 359 360 361 362 363 364 365 366 367 368 369 370 371 372 373 374 375 376 377 378 379 380 381 382 383 384 385 386 387 388 389 390 391 392 393 394 395 416 417 418 421 422 423 424 425 426 506 507 508 509 510 511 512 513 541 542 560 561 562 563 564 592 594 595 596 597 598 638 639 640 641 642 643 644 647 648 684 685 686 687 688 761 762 825 826 827 828 829 830 831 834 835 836 837 838 839 840 841 842 843 844 845 846 847 848 849 850 851 852 853 854 887 888 889 890 891 892 893 894 895 896 897 898 899 900 901 902 903 904 905 906 907 908 909 910 911 912 913 914 915 916 917 918 919 920 921 922 923 924 925 926 927 928 929 930 931 932 933 934 935 936 937 938 939 940 941 942 943 945 946 947 948 949 950 951 952 959 960 961 962 963 964 965 966 967 968 969 970 971 972 973 974 975 976 977 978 979 980 981 982 983 984 985 986 987 988 989 990 991 992 993 994 995 996 997 998 999 1000 1001 1002 1003 1004 1005 1006 1007 1008 1009 1010 1011 1012 1013 1014 1015 1016 1017 1018 1020 1021 1022 1023 1024 1025 1026 1027 1028 1029 1032 1034 1035 1036 1037 1038 1039 1040 1041 1042 1043 1044 1045 1046 1047 1048 1049 1050 1051 1052 1053 1054 1055 1056 1057 1058 1059 1060 1065 1066 1067 1068 1069 1070 1071 1072 1073 1074 1075 1076 1077 1078 1079 1080 1081 1082 1083 1084 1085 1086 1087 1088 1089 1091 1092 1094 1098 1099 1100 1101 1102 1103 1104 1105 1106 1107 1108 1109 1110 1111 1112 1113 1114 1120 1121 1122 1123 1124 1125 1126 1127 1128 1129 1130 1131 1132 1136 1137 1138 1139 1153 1154 1155 1156 1157 1158 1159 1160 1161 1162 1164 1165 1166 1179 1180 1181 1182 1183 1184 1185 1186 1187 1188 1189 1190 1191 1192 1193 1194 1195 1196 1197 1199 1200 1201 1202 1203 1204 1205 1206 1207 1208 1209 1210 1211 1212 1213 1214 1216 1217 1219 1220 1221 1222 1223 1224 1225 1226 1227 1228 1229 1230 1231 1232 1258 1259 1263 1264 1265 1266 1267 1268 1269 1270 1271 1272 1273 1274 1301 1304 1305 1306 1307 1308 1309 1310 1311 1312 1313 1314 1315 1316 1317 1320 1321 1322 1323 1324 1325 1326 1327 1330 1331 1332 1333 1356 1357 1358 1359 1360 1361 1362 1363 1364 1365 1367 1395 1396 1397 1398 1406 1407 1408 1410 1434 1435 1436 1437 1439 1456 1457 1458 1459 1460 1461 1462 1463 1464 1465 1466 1467 1468 1469 1470 1471 1472 1473 1474 1475 1476 1477 1478 1479 1480 1481 1482 1483 1484 1485 1486 1487 1488 1489 1490 1491 1492 1493 1494 1495 1496 1497 1498 1499 1500 1501 1502 1503 1504 1505 1506 1507 1508 1509 1510 1511 1512 1513 1514 1515 1516 1521 1522 1523 1524 1525 1526 1527 1529 1550 1551 1552 1553 1563 1564 1565 1566 1567 1568 1569 1570 1572 1573 1574 1575 1576 1577 1578 1579 1580 1581 1582 1583 1584 1585 1586 1587 1588 1589 1590 1591 1592 1593 1594 1595 1596 1597 1598 1599 1600 1601 1602 1603 1604 1605 1606 1607 1608 1610 1611 1612 1613 1614 1615 1616 1617 1618 1619 1659 1660 1661 1662 1663 1664 1665 1666 1667 1668 1669 1670 1671 1672 1673 1674 1675 1676 1677 1678 1679 1680 1681 1682 1683 1684 1685 1689 1690 1691 1692 1693 1694 1695 1696 1697 1698 1710 1711 1712 1713 1715 1716 1717 1718 1719 1720 1721 1722 1723 1724 1725 1726 1727 1728 1729 1730 1731 1732 1733 1734 1735 1746 1747 1748 1749 1750 1751 1752 1753 1754 1755 1756 1757 1758 1759 1760 1761 1762 1763 1764 1765 1766 1767 1768 1769 1770 1771 1781 1782 1783 1784 1785 1787 1788 1789 1790 1791 1792 1793 1794 1799 1800 1801 1802 1803 1804 1805 1806 1807 1808 1809 1810 1811 1812 1813 1814 1815 1816 1817 1818 1819 1820 1821 1822 1823 1824 1825 1826 1827 1828 1829 1830 1831 1832 1833 1834 1835 1836 1837 1838 1839 1840 1841 1842 1843 1844 1845 1846 1847 1848 1849 1850 1851 1852 1853 1854 1855 1856 1857 1858 1859 1860 1861 1862 1863 1864 1865 1866 1867 1868 1869 1870 1871 1872 1873 1874 1875 1876 1877 1878 1879 1880 1881 1882 1883 1884 1885 1886 1888 1889 1890 1891 1892 1893 1894 1895 1896 1897 1898 1899 1900 1901 1902 1903 1904 1905 1906 1907 1908 1909 1910 1911 1912 1913 1914 1915 1916 1917 1920 1921 1922 1923 1924 1925 1926 1927 1928 1929 1930 1931 1932 1933 1934 1935 1936 1937 1938 1939 1940 1941 1942 1943 1944 1945 1946 1947 1996 1998 1999 2000 2001 2002 2003 2004 2005 2006 2007 2008 2009 2010 2011 2012 2013 2014 2015 2016 2017 2018 2019 2020 2021 2022 2023 2024 2025 2057 2058 2059 2060 2061 2084 2087 2088 2089 2103 2104 2105 2106 2107 2108 2109 2110 2111 2112 2113 2114 2115 2116 2125 2126 2127 2128 2129 2130 2131 2132 2133 2134 2135 2136 2137 2138 2139 2140 2141 2142 2143 2144 2145 2146 2147 2149 2150 2151 2152 2153 2154 2155 2156 2157 2158 2159 2160 2161 2162 2163 2164 2165 2166 2167 2169 2170 2171 2174 2175 2176 2177 2178 2179 2180 2181 2182 2183 2184 2185 2186 2187 2188 2189 2190 2191 2192 2193 2194 2195 2200 2201 2202 2203 2204 2205 2206 2207 2208 2209 2210 2211 2212 2213 2214 2215 2216 2217 2218 2219 2220 2221 2222 2223 2224 2225 2226 2227 2228 2229 2230 2231 2232 2233 2234 2235 2236 2237 2238 2241 2242 2243 2244 2245 2246 2250 2251 2252 2253 2254 2255 2256 2257 2258 2259 2260 2261 2262 2263 2264 2265 2266 2267 2268 2269 2270 2271 2272 2273 2274 2275 2276 2277 2278 2279 2280 2281 2282 2283 2284 2285 2286 2287 2288 2289 2290 2291 2292 2293 2294 2295 2296 2297 2298 2299 2300 2301 2302 2303 2304 2305 2306 2307 2308 2317 2318 2319 2320 2321 2322 2323 2324 2325 2326 2327 2328 2329 2330 2331 2332 2333 2334 2335 2336 2337 2338 2339 2340 2341 2342 2343 2344 2345 2346 2347 2348 2349 2350 2351 2352 2353 2354 2355 2356 2357 2358 2359 2360 2361 2362 2363 2364 2365 2366 2367 2368 2369 2370 2371 2372 2373 2374 2375 2376 2377 2378 2379 2380 2381 2382 2383 2384 2385 2386 2387 2388 2389 2390 2391;

end;
